# Supplementary material for: Prevalence and determinants of caesarean section in private and public health facilities in underserved South Asian communities: cross-sectional analysis of data from Bangladesh, India and Nepal
Source: BMJ Open. 2014 Dec 30;4(12):e005982. doi: 10.1136/bmjopen-2014-005982 (PMC4283435; doi:10.1136/bmjopen-2014-005982)
Supplement: Author's manuscript [file bmjopen-2014-005982.draft_revisions.pdf]

Prevalence and determinants of caesarean section in private and public health facilities in underserved South Asian communities: cross-sectional analysis of data from Bangladesh, India, and Nepal

Journal:	*BMJ Open*
Manuscript ID:	bmjopen-2014-005982
Article Type:	Research
Date Submitted by the Author:	26-Jun-2014
Complete List of Authors:	Neuman, Melissa; UCL Institute for Global Health,<br>Alcock, Glyn; UCL Institute for Global Health,<br>Azad, Kishwar; Diabetic Association of Bangladesh, Perinatal Care Project<br>Kuddus, Abdul; Diabetic Association of Bangladesh, Perinatal Care Project<br>Osrin, David; UCL Institute for Global Health,<br>Shah More, Neena; Society for Nutrition, Education and Health Action (SNEHA),<br>Nair, Nirmala; Ekjut,<br>Tripathy, Prasanta; Ekjut,<br>Sikorski, Catherine; UCL Institute for Global Health,<br>Saville, Naomi; UCL Institute for Global Health,<br>Sen, Aman; Mother and Infant Research Activities (MIRA),<br>Colbourn, Timothy; UCL Institute for Global Health,<br>Houweling, Tanja; Erasmus MC University Medical Center, Department of Public Health<br>Seward, Nadine; UCL Institute for Global Health,<br>Manandhar, Dharma; Mother and Infant Research Activities (MIRA),<br>Shrestha, Bhim; Mother and Infant Research Activities (MIRA),<br>Costello, Anthony; UCL Institute for Global Health,<br>Prost, Audrey; UCL Institute for Global Health,
**Primary Subject Heading**:	Global health
Secondary Subject Heading:	Epidemiology, Obstetrics and gynaecology
Keywords:	Epidemiology < TROPICAL MEDICINE, OBSTETRICS, PUBLIC HEALTH

SCHOLARONE™ Manuscripts

Prevalence and determinants of caesarean section in private and public health facilities in underserved South Asian communities: cross-sectional analysis of data from Bangladesh, India, and Nepal

Melissa Neuman[1], Glyn Alcock[1], Kishwar Azad[4], Abdul Kuddus[4], David Osrin[1], Neena Shah More[3], Nirmala Nair[5], Prasanta Tripathy[5], Catherine Sikorski[1], Naomi Saville[1], Aman Sen[2], Tim Colbourn[1], Tanja AJ Houweling[6], Nadine Seward[1], Dharma S Manandhar[2], Bhim P Shrestha[2], Anthony Costello[1] and Audrey Prost[1]

Author affiliations:

[1] Institute for Global Health, University College London, 30 Guilford Street, London WC1N 1EH, UK

[2] Mother and Infant Research Activities (MIRA), YB Bhavan, Thapathali, GPO Box 921, Kathmandu, Nepal

[3] Society for Nutrition, Education and Health Action (SNEHA), Urban Health Centre, Shahunagar, Dharavi, Mumbai 400017, Maharashtra, India

[4] Perinatal Care Project, Diabetic Association of Bangladesh, 122 Kazi Nazrul Islam Avenue, Dhaka 1000, Bangladesh

[5] Ekjut, Plot 556B, Potka, Chakradharpur, West Singhbhum, Jharkhand, India

[6] Department of Public Health, Erasmus MC University Medical Center Rotterdam, P.O. Box 2040, 3000 CA Rotterdam, The Netherlands

Proposed running head: Caesarean sections in private vs. public health facilities in South Asia

Word count: 4393

Figures and tables: 0 figures and 4 tables

For peer review only

For peer review only - http://bmjopen.bmj.com/site/about/guidelines.xhtml

ABSTRACT (Max: 300 words; Current: 295)

Objectives: To describe the prevalence and determinants of births by caesarean section in private and public health facilities in underserved communities in South Asia.

Design: Cross-sectional study.

Setting: 81 community-based geographical clusters in four locations in Bangladesh, India and Nepal (three rural, one urban).

Participants: 45,327 births occurring in the study areas between 2005 and 2012.

Outcome measures: proportion of caesarean section delivery by location and type of facility; determinants of caesarean section delivery by location.

Results: Institutional delivery rates varied widely between settings, from 21% in rural India to 90% in urban India. The proportion of private and charitable facility births delivered by caesarean section was 73% in Bangladesh, 30% in rural Nepal, 18% in urban India, and 5% in rural India. The odds of caesarean section were greater in private and charitable health facilities than in public facilities in three of four study locations, even when adjusted for pregnancy and delivery characteristics, maternal characteristics, and year of delivery (Bangladesh AOR: 5.91, 95% CI: 5.15-6.78; Nepal AOR: 2.37, 95% CI: 1.62-3.44; urban India AOR: 1.22, 95% CI: 1.09-1.38). We found significant interactive associations between maternal education and private facility delivery in two of four locations (p=0.025 in rural Bangladesh and p<0.001 in urban India), with highly educated women particularly likely to deliver by caesarean in private facilities in both locations.

Conclusions:
Our results lend support to the hypothesis that increased caesarean section rates in these South Asian countries may be driven in part by the private sector. They also suggest that preferences for caesarean delivery may be higher among highly educated women, and that individual- and provider-level factors interact in driving caesarean rates higher. Rates of caesarean section in the private sector, and their maternal and neonatal health outcomes, require close monitoring.

For peer review only - http://bmjopen.bmj.com/site/about/guidelines.xhtml

For peer review only

Article summary

Article focus

- Caesarean sections are on the rise in South Asia. Research suggests that private health facilities may be contributing to this by conducting caesareans over and above clinical need.
- Literature from other settings indicates that increases in caesarean sections are shaped by both supply and demand pressures: providers often have financial incentives to intervene surgically, and women of higher socio-economic status are also more likely to want caesareans.
- This study examined the prevalence and determinants of caesarean sections in private and charitable facilities compared with public health facilities for over 45,000 births in underserved communities of rural Nepal, rural Bangladesh, rural and urban India.

Key messages

- Caesarean sections were more common in private than in public health facilities in three of four study locations (urban India, rural Nepal, and rural Bangladesh).
- We found significant interactive associations between maternal education and caesarean delivery in Bangladesh (both private and public facilities) and urban India (private facilities only).
- Future research should monitor caesarean section rates in the private sector in South Asia and subsequent maternal and neonatal health outcomes

Strengths and limitations

- This study had a large sample size and focused on underserved communities, which are a priority for public health interventions in South Asia.
- Our data were not nationally representative, which limits the generalisability of our findings. Other limitations included small denominators and missing data for some variables. We did not have data on some known predictors of caesarean section, which would have enhanced the completeness of our determinants analysis.

For peer review only - http://bmjopen.bmj.com/site/about/guidelines.xhtml

For peer review only

INTRODUCTION

Access to comprehensive emergency obstetric care, including caesarean section, is key to preventing the estimated 287,000 maternal and 2.9 million neonatal deaths that occur worldwide every year.[1 2] Although debate continues about how to quantify the need for life-saving obstetric surgery, a 1985 World Health Organization (WHO) report suggested that the optimal population range for caesarean section rates is between 5% and 15%, and this endures as a reference.[3 4] Caesarean section rates are increasing worldwide, albeit unequally: a recent analysis of Demographic and Health Survey (DHS) data in 26 South Asian and sub-Saharan African countries found that rates were highest among the 'urban rich' in all countries, and lowest among the 'rural poor' in 18.[5] In all countries, fewer than 5% of mothers in the poorest wealth quintile delivered by caesarean.[6]

Caesarean sections conducted without clinical need can have adverse consequences for mothers and children. A 2008 WHO survey of 373 facilities across 24 countries found that unnecessary caesareans were associated with an increased risk of maternal mortality and serious outcomes for mothers and newborn infants, compared with spontaneous vaginal delivery.[7] Recent ecological analyses also highlighted strong associations between caesarean delivery and increased neonatal mortality in countries with low and medium caesarean section rates.[8] Unnecessary caesareans lead to considerable costs for families and health systems: an estimated 6.2 million unnecessary procedures were performed in 2008, costing approximately US$ 2.32 billion. [9]

Several South Asian countries have recorded substantial increases in caesarean section rates over the past decade. In Bangladesh, rates rose from 2% (2000) to 17% (2011); in India, from 3% (1992) to 11 % (2006); and in Nepal, from 1% (2000) to 5% (2011).[10-5] Several studies in these countries have raised concerns about high caesarean rates in private facilities, and a recent DHS analysis speculated that national increases in caesarean section rates in South Asian countries could be driven in part by higher rates among deliveries in private sector facilities[.6 16-18] Literature from other settings indicates that increases in caesarean sections are shaped by both supply and demand pressures: providers often have financial incentives to intervene surgically, and women of higher socio-economic status are also more likely to desire caesareans.[19 20]

There are few large, recent community-based studies from South Asia quantifying differences in caesarean section rates between public and private facilities. Such studies are necessary in order to examine whether increases in caesareans in these settings are indeed likely to be driven by the private sector, demand from wealthier and more educated mothers, or a combination of the two. We conducted a cross-sectional analysis of data from Bangladesh, India, and Nepal to explore the prevalence and determinants of caesarean section delivery by type of facility and location, and to examine the

For peer review only - http://bmjopen.bmj.com/site/about/guidelines.xhtml

interactions between maternal education and caesarean section delivery in private and public facilities.

METHODS

Study populations

We used data collected through vital events surveillance systems established during four cluster-randomised controlled trials conducted between 2005 and 2011. The trials were done with communities that can be considered socio-economically disadvantaged: in Bangladesh and Nepal, they took place in four rural, underserved districts (Bogra, Maulvibazaar, and Faridpur in Bangladesh, and the Terai district of Dhanusha, Nepal); in rural India, most participants were from Scheduled Tribes in two states of eastern India (Jharkhand and Odisha); in urban India, data came from slum communities in Mumbai. **Table 1** describes the characteristics of each study and its population, including the background maternal mortality ratio and types of facilities present in the study areas. The original trials were designed to evaluate the impact of participatory women's groups on maternal and neonatal health outcomes.[21-25] We used data from the control areas of these trials only, because the women's group intervention led to changes in mortality and practices in several locations.[26]

Health system contexts

All three study countries have experienced substantial increases in institutional delivery rates over the last two decades: births in health facilities increased from 4% (1993) to 29% (2011) in Bangladesh; from 26% (1992-3) to 47% (2007-8) in India; and from 8% (1996) to 35% (2011) in Nepal.[10-15] All three countries have implemented incentive schemes to promote institutional delivery, though these have varying coverage. In 2010, Bangladesh's pilot maternity voucher scheme reached an estimated 10.4 million people across 31 sub-districts, around 7% of the country's population.[27 28] Mothers receive a cash incentive for antenatal care and delivery in a public or private facility, or at home with a skilled birth attendant. Both government and private facility staff also receive cash incentives, including 3000 Bangladeshi Taka (US $38.5) for a caesarean section and 300 Taka for a normal delivery.[28] This maternity incentive scheme was operational in two of the three districts (Faridpur and Maulvibazaar) covered by our study. All three districts had public facilities including District Hospitals, Maternal and Child Welfare Centres, and Upazilla Health Complexes. Private facilities included a number of small-to-medium-size clinics, BRAC (NGO) facilities, and larger private hospitals.

In India, the *Janani Suraksha Yojana* (JSY) maternity incentive scheme entitles women in rural areas of high focus states, including those in this study, to 1400 Indian Rupees ($22.4) after delivering in a government or accredited private health facility. Local community health volunteers called Accredited Social Health Activists (ASHAs) also receive 600 Indian Rupees ($9.6) for identifying pregnant women and helping them get to a health facility.[29] Although the rural Indian data included in this study were

For peer review only - http://bmjopen.bmj.com/site/about/guidelines.xhtml

collected between 2005 and 2008, the JSY was only operational in the study areas from 2008 onwards and its impact is unlikely to be reflected here. The Indian urban area included in our study spanned informal settlements (slums) with a wealth of public and private providers.[30] Mothers with Below Poverty Line cards in such areas are eligible for JSY and can receive Rs 500 towards the costs of delivering in a health facility.[31] Nepal began a safe delivery incentive scheme in 2005 and free deliveries have been available in government facilities since 2009 through the *Aama Surakshya* programme.[32] Dhanusha district, from which data for this study come, has one zonal tertiary hospital and a private medical college hospital equipped for c-sections, and a variety of small public and private health facilities without comprehensive obstetric care.

Data collection

All data were collected using surveillance systems to monitor births and deaths prospectively.[21-25] In all study locations, a female community-based key informant reported births and deaths in her area, which covered a population ranging from 250 to 350. A trained interviewer then verified these reports and paid the informant an incentive for each correct identification. In Bangladesh, rural and urban India, the interviewer administered a structured questionnaire to all eligible mothers around six weeks after delivery; in Nepal, all births in the study area were registered, and interviews were conducted on all births in small clusters and on a random sample of 10 births per month in the larger clusters. In each study location, mothers were interviewed using a questionnaire to collect information about events in the antenatal, delivery and postnatal periods. In each trial and in this subsequent study, participants were women of reproductive age (15-49) who delivered in the study area during the data collection period, and who consented to be interviewed six weeks after delivery, and their infants.

Study sample

The initial sample for this analysis included 46,393 births in Mumbai, rural India, rural Bangladesh, or rural Nepal, of whom 17,565 (38%) were delivered in health care facilities. We excluded the 2348 births occurring outside Mumbai, because we collected limited information on delivery location for these. Additionally, 223 deliveries had missing data on form of delivery (caesarean or vaginal), 602 had missing or implausible values for mother's age, and 98 were missing information on other maternal characteristics (educational attainment, household assets, number of prior pregnancies, number of antenatal visits, severe problems in pregnancy or delivery). 143 births had missing or incomplete information on place of delivery. The final analytic sample included 45,327 births (97.7% of the initial sample).

For peer review only - http://bmjopen.bmj.com/site/about/guidelines.xhtml

Measures used

The primary outcome in these analyses was caesarean section delivery, identified by self-report from the mother or another household member around six weeks after giving birth. The main covariate of interest was the type of delivery facility, coded as public (e.g. district hospitals), private (individual private clinics or hospitals), or NGO/charitable (e.g. Christian missionary hospitals in rural India; BRAC clinics in Bangladesh). We grouped private and NGO/charitable facilities together due to the small number of births in NGO/charitable facilities (6% of institutional deliveries in Bangladesh, 7% in rural India, and 0 in urban India and rural Nepal; data on request). Additional covariates in the models included measures of the characteristics of the pregnancy and delivery, maternal socio-demographic characteristics, and the location in which the delivery occurred. We created an indicator variable to identify women experiencing serious problems during pregnancy or delivery, with women reporting symptoms of pre-eclampsia (blurred vision or swelling of face and hands), symptoms of eclampsia (fits or seizures during pregnancy or delivery), hæmorrhage during delivery, or labour lasting more than 24 hours considered to have serious complications. Other characteristics of the pregnancy and delivery considered in the analysis included full utilisation of antenatal care, number of prior pregnancies, and multiple pregnancy. Full utilization of antenatal care was defined as four or more visits to antenatal care, with at least one visit to a skilled provider. Number of pregnancies was entered as an ordered categorical variable, with categories of one (first), two, three, or four or more pregnancies.

Maternal socio-demographic characteristics included in the models were: mother's age at delivery, her educational attainment, and household assets. Mother's age was entered into the model as a categorical measure in 10-year groups. Educational attainment was entered as a categorical variable using the following categories: no formal education, primary education, secondary education, or bachelor's degree or higher. To develop an asset index, we used polychoric factor analysis on data on common assets and amenities found in the mother's household, and grouped the resulting factor scores into quartiles.[33] Assets and amenities included electricity, radio or cassette player, electric fan (Bangladesh and India only), television, refrigerator (Bangladesh and India only), telephone (Bangladesh and Nepal only), generator (India only), and bicycle. All models were additionally adjusted for location (Bangladesh, India, Nepal), and year of interview in three-year groups. The data were collected in a stratified, cluster-sampled survey, and we accounted for survey design in the analysis using a fixed effect for stratum and a random effect for cluster.

Statistical analysis

We used frequencies to describe caesarean section rates by delivery location at each site. We used the Generalized Linear Latent And Mixed Models (GLLAMM) procedure in Stata 13.1, with adaptive quadrature for binary outcomes, to estimate the crude association between type of delivery facility and

For peer review only - http://bmjopen.bmj.com/site/about/guidelines.xhtml

caesarean section.[34 35] We identified other maternal, pregnancy, and delivery characteristics potentially associated with caesarean section using existing literature, especially studies resulting from the WHO multi-country surveys, and entered these in adjusted models to explore how they modified the association between type of delivery facility and caesarean section, and their individual association with caesarean section. Some South Asian and Latin American studies have detected a strong association between maternal education and caesarean delivery. [7 36 37] We fitted models including indicator variables for each group of mothers by education and type of delivery facility to explore differences in the strength of association between caesarean delivery and private facility by mother's education. To account for the sampling procedure used in rural Nepal, models were adjusted using pweights (probability of selection within cluster); these weights were rescaled to reflect the total number of institutional deliveries.

Ethical approval

The trials that provided data for this study received ethical approval from the following committees: the ethics committee of the Diabetic Association of Bangladesh (Perinatal Care Project, Bangladesh Diabetes Somity, BADAS); an independent ethics committee in Jamshedpur, Jharkhand, India; the Nepal Health Research Council; the Municipal Corporation of Greater Mumbai and the Independent Ethics Committee for Research on Human Subjects (Mumbai, India); the ethics committee of the Institute of Child Health, University College London.

RESULTS

Table 2 describes the characteristics of institutional births and caesarean deliveries by location. We analysed data for 45,327 births: 21,560 in rural Bangladesh, 8,541 in rural India, 10,236 in urban India, and 4,931 in rural Nepal. The proportion of women delivering in health facilities varied widely between locations, from 90% in urban India to 21% in rural India There were also large variations in the proportion of women delivering in private/charitable facilities rather than public facilities, with the highest (77%) in rural India and the lowest (10%) in rural Nepal. The proportion of women giving birth by caesarean section in private rather than public facilities also varied widely between settings. In Bangladesh, only 21% of women delivered in a health facility, around half of them in the private/charitable sector, but 73% of private facility births were by caesarean section. In rural Nepal, 30% of the 162 private facility births were by caesarean section. In informal settlements of Mumbai, 15% of public facility deliveries were caesareans, compared with 18% of private facility deliveries. In rural India, caesarean sections were more commonly performed in public health facilities than private or charitable facilities (15% vs. 5%). **Supplemental table 1** describes the characteristics of institutional deliveries (caesarean or non-caesarean) according to type of delivery facility (private or public), maternal

For peer review only - http://bmjopen.bmj.com/site/about/guidelines.xhtml

For peer review only

socio-economic and socio-demographic characteristics, pregnancy and delivery characteristics, and year of delivery.

Table 3 shows crude and adjusted measures of association between type of delivery facility (private or public) and caesarean section for each location. Delivering in a private health facility was associated with an increased odds of caesarean section in all but one location (rural India). The relative odds of a caesarean in a private facility were greatest in Bangladesh (OR: 6.82, 95% CI: 5.96, 7.81), followed by Nepal (OR: 2.42, 95% CI: 1.48, 3.94) and urban India (OR 1.36, 95% CI: 1.21, 1.52). These associations persisted and were only mildly attenuated when adjusted for maternal characteristics, pregnancy and delivery characteristics, and year of delivery (Bangladesh AOR: 5.91, 95% CI: 5.15, 6.78; Nepal AOR: 2.37, 95% CI: 1.62, 3.44; urban India AOR: 1.22, 95% CI: 1.09, 1.38).

Women who had four or more antenatal check-ups were significantly more likely to have a caesarean delivery in rural Bangladesh and Nepal, with positive but non-significant trends in all other locations. Parity was not associated with caesarean delivery in any location. Having a serious health complication during pregnancy and delivery was associated with caesarean delivery in all locations except rural Bangladesh, where we observed a negative association (AOR: 0.87, 95% CI: 0.76, 1.00). Multiple birth was associated with an increased odds of caesarean section only in urban India and in rural Nepal. Higher maternal age was only associated with an increased odds of caesarean section in urban India. Maternal education was only associated with an increased odds of caesarean delivery in rural Bangladesh and urban India, with mothers with secondary education or Bachelor's degrees having higher odds (AOR: 1.44, 95% CI: 1.13, 1.84 and AOR 2.44, 95% CI: 1.52, 3.92 for Bangladesh and AOR: 1.22, 95% CI: 1.04, 1.42 and 1.62, 95% CI: 1.30, 2.02 for urban India, respectively). There were significant positive associations only in rural Bangladesh, with small increases in odds of caesarean section for unit increase in wealth quartile (AOR comparing wealthiest to poorest group in Bangladesh: 1.36, 95% CI: 1.09,1.70), and in urban India, for the wealthiest quartile (AOR: 1.50, 95% CI: 1.27, 1.78).

We found interactive associations between maternal education and private facility delivery in two of four sites (p=0.021 in rural Bangladesh and p<0.001 in urban India; **table 4**). In Bangladesh, there was a pronounced positive educational gradient in caesarean delivery within those who delivered in private facilities, as well as a clear positive association between private facility delivery and caesarean delivery. Women with bachelor's degrees delivering in private facilities had 11 times greater odds of delivering by caesarean (AOR 11.09, 95% CI: 6.28, 19.57) than did women with no formal education delivering in public facilities. Women with no formal education delivering in private facilities had nearly seven times the odds of caesarean compared with the reference group (AOR 6.94, 95% CI: 4.64, 10.39). A similar positive gradient was seen among women delivering in public facilities, with women with bachelor's

For peer review only - http://bmjopen.bmj.com/site/about/guidelines.xhtml

education having four times greater odds of caesarean delivery in public facilities than women with no formal education (AOR 4.55, 95% CI: 2.22, 9.33). There was also an educational gradient among women delivering in private facilities in urban India. Educated mothers delivering in private facilities were more likely to deliver by caesarean than were mothers in other groups (AOR 2.10; 95% CI: 1.61, 2.75). However, there was no apparent gradient by educational attainment among women delivering in public facilities.

DISCUSSION

Our analysis of data from over 45,000 births confirms the findings of other studies identifying differences in caesarean rates between public and private facilities, and suggests that, even in underserved areas in South Asia, caesareans without medical indication are of concern. In three of four locations, rates of caesarean section were higher in private/charitable facilities than in public facilities. The findings from Bangladesh are particularly noteworthy as they show much greater odds of caesarean section in private facilities, concurring with previous analyses.[37] This was also the only location where serious complications in pregnancy and delivery were not associated with caesarean delivery, suggesting that obstetric surgery was performed over and above clinical need.

Our findings confirm the results of earlier studies of the prevalence of caesarean delivery in South Asia, and indicate that high rates can be found in underserved rural areas. In India, a recent analysis of 2010-11 Annual Health Survey (AHS) data from 284 districts in nine States, including Jharkhand, found that the median caesarean section rate in the private sector was 28%, compared with 5% in the public sector.[38] There appear to be strong financial incentives for surgical procedures in the private sector.[39] In a rural, largely indigenous part of eastern India where more mothers delivered in private/charitable facilities than in public facilities, more caesarean sections were performed in the public sector. In this particular setting, our field experience suggests that only women with serious complications would go to a facility and have a caesarean section, especially as JSY was not yet available in the study areas at the time of data collection. Such women are likely to have experienced multiple referrals from either ill-equipped public or private facilities not wanting to take the risk of admission. It is however possible that preference for the private sector has changed in Jharkhand since the advent of JSY, and further disaggregated analyses of Annual Health Survey data would allow a more contemporary exploration of State-level variations in caesarean section rates between public and private sectors.

In Mumbai slums with a high uptake of institutional births and a 60/40 split in favour of the public sector for delivery care, we found no difference in caesarean section rates between the two sectors, which is somewhat surprising given the high rates of caesareans observed in the private sector in nine other

For peer review only - http://bmjopen.bmj.com/site/about/guidelines.xhtml

states of India (excluding Maharashtra). At least two scenarios may be relevant in this setting. First, public sector hospitals are able to provide caesarean section, while smaller private maternity homes may not be able to. Second, the costs of caesarean section borne by private sector providers are higher in Mumbai, and families may be unwilling to bear these additional costs. This provides motivation for private providers not to provide caesareans, and makes it likely that women experiencing complications move into the public sector. The variety of facility options in an urban environment means that mothers are able to choose between different types of facilities, while in rural areas only one provider may be accessible.

Analyses using the most recent Bangladesh DHS found that, in 2011, three in five facility births were delivered by caesarean section. This reflects a historical trend: in 2001-2003, nearly half of deliveries in private facilities in Bangladesh were already by caesarean section.[18] Our data from rural, socio-economically disadvantaged communities in three districts of Bangladesh confirm these population-level findings, but also suggest that high caesarean section rates in private facilities are not merely an issue for wealthy urban mothers. Although some research from Bangladesh suggests that mothers may have a preference for caesarean delivery because of fear of labour pain or a desire to select an auspicious date for the birth, other studies also highlight women's fears of caesarean section and their distrust of health providers who recommend them, mainly because of the high costs associated with the procedure.[37 40 41] A qualitative study involving twenty women who had experienced obstetric complications in Matlab in 2008-9 found that most of the fourteen women who had undergone caesareans had spent over Tk 14,999 (US$ 217) on the procedure, which was approximately one-third of GDP per capita at the time.[41] The lack of association between caesareans and complications in pregnancy or delivery and multiple births, coupled with the high financial incentives given to providers for performing caesarean sections and the requirement for junior doctors to 'practise' their surgical skills, further suggest that obstetric surgery is being used over and above clinical need. A possible explanation for the significant interactive associations between maternal education and caesarean delivery in both public and private facilities in Bangladesh is that well-educated women may be delivering in more expensive or highly rated institutions, which may in turn be more likely to do caesarean sections for financial reasons and if they act as training centres for junior doctors.

In our sample of institutional deliveries from rural Nepal, 16% of facility births were by caesarean, which is higher than the national average: the 2011 Nepal DHS found an overall national caesarean section rate of 5%, with sections more commonly performed for births to highly educated mothers (13%) and mothers in the highest wealth quintile (14%).[26] It is possible that women with complications are more likely to deliver in facilities, and also that, as in Bangladesh, private providers are motivated by financial incentives to conduct caesareans more frequently than strictly necessary. Women who can afford to

For peer review only - http://bmjopen.bmj.com/site/about/guidelines.xhtml

seek care in private facilities may also be more willing or able to pay for caesarean sections, and providers may conduct more of them to increase their income.

A 2010 analysis of DHS data examining the role of the private sector in maternity care in 16 countries found evidence of a trend towards privatisation in delivery care between the 1990s and mid-2000s, but with strong differences between countries, which might reflect the heterogeneous nature of this sector both between and within countries. [42] This DHS study highlighted the need for more context-specific data on the nature of the private sector in low and middle-income countries, and its role in maternity care. Further research might focus on understanding the motivations and experiences of women undergoing caesarean sections in private facilities in South Asian settings, pathways for switching between public and private sectors in the event of obstetric complications, a more comprehensive tally of the financial incentives (official or non-official) that motivate private providers to carry out caesareans in each setting, and the consequences of increased caesarean sections in the private sector for maternal and neonatal health outcomes.

In our study, locations with higher prevalence of caesarean deliveries also showed a positive educational gradient even after adjusting for wealth measured by household assets. Moreover, there was an interactive association between education and type of facility, with highly educated women particularly likely to receive caesarean deliveries in private facilities in Bangladesh and urban India. Literature exploring the determinants of caesarean delivery emphasises that multiple influences drive a woman's decision to deliver by caesarean. Profit considerations at the facility level may prompt some providers to urge women to receive unnecessary procedures, while women themselves may prefer caesareans for cultural reasons, fear of painful deliveries, or because they believe it to be safer. [36 43-45] Within the client-provider interaction, providers may be more likely to acquiesce to a request for caesarean from a highly educated woman; and such women may be more likely to accept advice from a provider. [19 46] Our findings lend support to the hypothesis that, while provider-level factors are probably partially responsible for the rapid increase in caesarean deliveries, it is also necessary to consider women's own preferences and decision-making processes and how they are shaped by social and cultural factors.

Strengths and limitations

The strengths of the study were its large sample size and focus on underserved communities, which are a priority for public health interventions in South Asia. It had five main limitations. It was not a nationally representative study, and districts or clusters were sampled purposively from previous cRCTs. This limits the generalisability of our findings to geographical settings outside the study areas. The study was cross-sectional, and therefore only able to suggest associations rather than causal relationships. The data also did not include key predictors of caesarean section such as breech presentation, and whether the

For peer review only - http://bmjopen.bmj.com/site/about/guidelines.xhtml

previous delivery was by caesarean, which limits the completeness of our analysis of determinants. In addition, levels of serious complications in pregnancy and delivery varied considerably between locations and were often higher than expected, suggesting potential over-reporting and limited reliability as an indicator of complications. Finally, some variables had small denominators, and others had high levels of missing data (for example, maternal age in rural India).

Conclusions

Our study found that delivering in a private health facility was associated with an increased odds of caesarean section in three of four South Asian locations, and that the associations persisted after adjustments for maternal, pregnancy and delivery characteristics, and year of delivery. We also found significant interactive associations between maternal education and caesarean delivery in Bangladesh (both private and public facilities) and urban India (private facilities only). These results lend support to the hypothesis that increased caesarean section rates in these three South Asian countries may in part be driven by the private sector, but also suggest that, in some settings such as Bangladesh and urban India, demand from more educated mothers may play a part. These findings call for greater, local understanding of the role of private providers in maternity care, together with careful examination of the consequences of increased caesarean sections in the private sector for maternal and neonatal health outcomes.

For peer review only

For peer review only - http://bmjopen.bmj.com/site/about/guidelines.xhtml

Funding

MN and NS are funded through a Wellcome Trust Strategic Award (085417MA/Z/08/Z). In Bangladesh, the Perinatal Care Project trial (covering KA and AK) was registered with trial number ISRCTN54792066 and has received funding from the Big Lottery Fund and the Wellcome Trust. In Eastern India, the Ekjut trial (covering NN and PT) was registered with trial number ISRCTN21817853 and funded by the Health Foundation. The Dhanusha (Nepal) trial (covering DSM, NS, BPS, and CS) was registered with trial number ISRCTN87820538 and has received funding from: the UBS Optimus Foundation; USAID; DfID and the Wellcome Trust. The City Initiative for Newborn Health trial led by SNEHA (Mumbai, India) was registered with trial number ISRCTN96256793 and has received funding from the ICICI Foundation for Inclusive Growth and the Wellcome Trust. GA, NSM, and DO are currently funded by The Wellcome Trust (091561/Z/10/Z). The funders played no part in the development of the article or the decision to submit it for publication.

Contributors

MN and AP had the original idea for the study. MN designed the data analysis strategy in discussion with AP. MN, GA, AS, CS and NS prepared the data. MN performed the analysis and prepared the tables. NN, PT, NSM, DO, KA, DM and AC are responsible for the trials. AP wrote the first draft of the paper and all authors read and commented on the draft. All authors read and approved the final manuscript.

Competing interests

None to declare

Data Sharing Statement

Data used in the analyses for this study are available upon request, subject to completion of a data sharing agreement. Please contact audrey.prost@ucl.ac.uk for further information.

Provenance and peer review

Not commissioned; externally peer reviewed.

For peer review only - http://bmjopen.bmj.com/site/about/guidelines.xhtml

For peer review only

REFERENCES:

1. World Health Organisation and UNICEF. Countdown 2015: Building a future for women and Children, the 2012 Report. http://countdown2015mnch.org/documents/2012Report/2012-Complete.pdf [Accessed 9th November 2013]
2. Campbell OM, Graham WJ. Strategies for reducing maternal mortality: getting on with what works. *The Lancet* 2006; 368: 1284-1299.
3. Ronsmans C, De Brouwere V, Dubourg D, *et al*.. Measuring the need for life-saving obstetric surgery in developing countries. *BJOG* 2004; 111: 1027-30.
4. World Health Organization. Appropriate technology for birth. *The Lancet* 1985; 2: 436-7.
5. Betrán AP, Merialdi M, Lauer JA, *et al*. Rates of caesarean section: analysis of global, regional and national estimates. *Paediatric and Perinatal Epidemiology* 2007; 21: 98-113.
6. Cavallaro FL, Cresswell JA, França GVA, *et al*. Trends in Caesarean delivery by country and wealth quintile: cross-sectional surveys in southern Asia and sub-Saharan Africa. *WHO Bulletin* 2013; 91: 897-972.
7. Souza JP, Gülmezoglu A, Lumbiganon P, *et al*. WHO Global Survey on Maternal and Perinatal Health Research Group. Caesarean section without medical indications is associated with an increased risk of adverse short-term maternal outcomes: the 2004–2008 WHO Global Survey on Maternal and Perinatal Health. *BMC Medicine* 2010; 8:71.
8. Kyu HH, Shannon HS, Georgiades K, Boyle MH. Caesarian delivery and neonatal mortality rates in 46 low- and middle-income countries: a propensity score matching and meta-analysis of demographic health survey data. *International Journal of Epidemiology* 2013; 1-11.
9. Gibbons L, Belizán JM, Lauer JA, *et al*. The global numbers and costs of additionally needed and unnecessary caesarean sections performed per year: overuse as a barrier to universal coverage. World Health Report (2010), Background paper. Geneva: World Health Organization.
10. Mitra SN, Nawab Ali M, Islam S, *et al*. Bangladesh Demographic and Health Survey, 1993-1994. Calverton, Maryland: National Institute of Population Research and Training (NIPORT), Mitra and Associates, and Macro International Inc, 1994.
11. National Institute of Population Research and Training (NIPORT), Mitra and Associates, and ICF International. Bangladesh Demographic and Health Survey 2011. Dhaka, Bangladesh and Calverton, Maryland, USA: NIPORT, Mitra and Associates, and ICF International, 2013.
12. International Institute for Population Sciences (IIPS) and Macro International. National Family Health Survey (NFHS 1), 1992–93. India, Mumbai: IIPS, 1995.
13. International Institute for Population Sciences (IIPS), Macro International. National Family Health Survey (NFHS 3), 2005–06; India: Volume I. Mumbai: IIPS, 2007.

For peer review only - http://bmjopen.bmj.com/site/about/guidelines.xhtml

For peer review only

14. Pradhan A, Aryal RH, Regmi G, *et al*. Nepal Family Health Survey 1996. Kathmandu, Nepal and Calverton, Maryland: Ministry of Health [Nepal], New ERA, and Macro International Inc, 1997.
15. Ministry of Health and Population (MOHP) [Nepal], New ERA, and ICF International Inc. Nepal Demographic and Health Survey 2011. Kathmandu, Nepal: Ministry of Health and Population, New ERA, and ICF International, Calverton, Maryland, 2012.
16. Ajeet S, Nandkishore K. The boom in unnecessary caesarean surgeries is jeopardizing women's health. *Health Care Women International* 2013; 34: 513-521.
17. Sreevidya S, Sathiyasekaran BW. High caesarean rates in Madras (India): a population-based cross-sectional study. *BJOG* 2003; 110: 106-111.
18. Koblinksi M, Anwar I, Mridha MK, *et al*. Reducing maternal mortality and improving maternal health: Bangladesh and MDG 5. *J Health Popul Nutr* 2008; 26: 280-294.
19. Leone T, Padmadas SS, Matthews Z. Community factors influencing rising caesarean section rates in developing countries: an analysis of six countries. *Soc Sci Med* 2008; 67: 1236-46.
20. Mazzoni A, Althabe A, Liu NH, *et al*. Women's preference for caesarean section: a systematic review and meta-analysis of observational studies. *BJOG* 2011; 118: 391-399.
21. Tripathy P, Nair N, Barnett S, et al. Effect of a participatory intervention with women's groups on birth outcomes and maternal depression in Jharkhand and Orissa, India: A cluster-randomised controlled trial. *Lancet* 2010; 375: 1182–1192.
22. Azad K, Barnett S, Banerjee B, *et al*. Effect of scaling up women's groups on birth outcomes in three rural districts in Bangladesh: a cluster-randomised controlled trial. *Lancet* 2010; 375: 1193–1202.
23. Fottrell E, Azad K, Kuddus A, *et al*. The effect of increased coverage of participatory women's groups on neonatal mortality in Bangladesh: a cluster-randomised trial. *JAMA Pediatrics* 2013; 167: 816-825.
24. Shah More N, Bapat U, Das S, *et al*. Cluster-randomised controlled trial of community mobilisation in Mumbai slums to improve care during pregnancy, delivery, postpartum and for the newborn. *Trials* 2008; 9:7.
25. Shrestha BP, Bhandari B, Manandhar DS, *et al*. Community interventions to reduce child mortality in Dhanusha, Nepal: study protocol for a cluster randomized controlled trial. *Trials* 2011; 12: 136.
26. Prost A, Colbourn T, Seward N, *et al*. Women's groups practising participatory learning and action to improve maternal and newborn health in low-resource settings: a systematic review and meta-analysis. *Lancet* 2013; 381: 1736–46.
27. Schmidt J-0, Ensor T, Hossain A, Khan S. Vouchers as demand side financing instruments for health care: A review of the Bangladesh maternal voucher scheme. *Health Policy* 2010; 96: 98–107.

For peer review only - http://bmjopen.bmj.com/site/about/guidelines.xhtml

28. Ahmed S, Khan MM. A maternal health voucher scheme: what have we learned from the demand-side financing scheme in Bangladesh? *Health Policy & Planning* 2011; 6:25–32.

29. Lim SS, Dandona L, Hoisington JA, *et al*. India's Janani Suraksha Yojana, a conditional cash transfer programme to increase births in health facilities: an impact evaluation**.** *Lancet* 2010; 375: 209-23.

30. Shah More N, Alcock G, Bapat U, *et al*. Tracing pathways from antenatal to delivery care for women in Mumbai, India: cross-sectional study of maternity in low-income areas. International Health 2009; 1: 71–77.

31. Government of India, Ministry of Health & Family Welfare. Janani Suraksha Yojana: Guidelines for Implementation, 2006. http://www.mohfw.nic.in/NRHM/./guidelines/JSY_guidelines_09_06.pdf

32. Witter S, Khadka S, Nath H, Tiwari S. The national free delivery policy in Nepal: early evidence of its effects on health facilities. *Health Policy and Planning* 2011; 26: ii84–ii91.

33. Holgado-Tello FP,Chacón–Moscoso S, Barbero-Garcia I, Vila-Abad E. Polychoric versus Pearson correlations in exploratory and confirmatory factor analysis of ordinal variables. *Quality & Quantity* 2010; 44 (1): 153-166.

34. Rabe-Hesketh S, Skrondal A, Pickles A. Maximum likelihood estimation of limited and discrete dependent variable models with nested random effects. *Journal of Econometrics* 2005; 128: 301-323.

35. StataCorp. 2013. *Stata Statistical Software: Release 13*. College Station, TX: StataCorp LP.

36. Potter J, Berquó E, Perpétuo I, *et al*. Unwanted Caesarean Sections among Public and Private Patients in Brazil: Prospective Study. *BMJ* 2001; 323: 1155-8.

37. Kamal SMM. Preference for institutional delivery and caesarean sections in Bangladesh. *J Health Popul Nutr* 2013; 31: 96-109.

38. Randive B, Diwan V, De Costa A. India's Conditional Cash Transfer Programme (the JSY) to Promote Institutional Birth: Is There an Association between Institutional Birth Proportion and Maternal Mortality? *PLoS ONE* 2013; 8(6): e67452. doi:10.1371/journal.pone.0067452

39. Chatterjee S, Laxminarayan R. Costs of surgical procedures in Indian hospitals. *BMJ Open* 2013; 3: e002844.

40. Parkhurst JO, Rahman SA. Life saving or money wasting? Perceptions of caesarean sections among users of services in Rural Bangladesh. *Health Policy* 2007; 80: 392-401.

41. Khan R, Blum LS, Sultana M, et al. An Examination of Women Experiencing Obstetric Complications Requiring Emergency Care: Perceptions and Sociocultural Consequences�of Caesarean Sections in Bangladesh. *J Health Popul Nutr 2012*; 30: 159-171.

42. Pomeroy A, Koblinksy M, Alva S. Private Delivery Care in Developing Countries: Trends and Determinants. DHS Working document 2010; No.76.

For peer review only - http://bmjopen.bmj.com/site/about/guidelines.xhtml

http://dhsprogram.com/pubs/pdf/WP76/WP76.pdf [Accessed 3rd March 2014].

43. Sufang G, Padmadas SS, Fengmin Z et al. Delivery settings and caesarean section rates in China. *Bull WHO* 2007; 85: 733-820.

44. Lo JC. Patients' attitudes vs. physicians' determination: implications for cesarean sections. *Soc Sci Med* 2003; 57: 91-6.

45. McCourt C, Weaver J, Statham H, *et al.* Elective cesarean section and decision making: a critical review of the literature. *Birth* 2007; 34: 65-79.

46. Ghetti C, Chan BKS, Guise J-M. Physicians' responses to patient-requested caesarean delivery. *Birth* 2004; 31: 280-4.

For peer review only

Table 1: Characteristics of studies and populations

**Study (Country)**	**Bangladesh (rural)**	**India (rural)**	**Nepal (rural)**	**India (urban)**
**Location**	Three districts: Bogra, Maulvibazaar and Faridpur	Three districts of Jharkhand and Odisha: Keonjhar, West Singhbhum and Saraikela	Dhanusha district (Terai)	Mumbai slums
**Period**	2005 – 2011	2005-2008	2008-2011	2006-2009
**Estimated population**	532,900	114,000	240,000	283,000
**Cluster characteristics**	Villages making up a union	8-10 villages with residents classified as Scheduled Tribe or Other Backward Class	Village Development Committee	Slum areas in six municipal wards of Mumbai
**Method of cluster identification**	Purposive sampling of three districts and clusters within districts	Purposive sampling of three districts and clusters within districts	Random sampling of 60 clusters from a list of 79 suitable clusters in one district	92 clusters in six municipal wards identified using municipal documents, surveys, discussions with key informants, and site visits. Random selection of 48 clusters for randomized allocation.
**Clusters, n**	9	18	30	24
**Cluster and individual follow-up**	All clusters followed up<br>Interviews completed after 82% of identified births in control areas in Phase 1, and 99% of births in Phase 2	All clusters followed up<br>Interviews completed after 98% of identified births	All clusters followed up	All clusters followed up<br>Interviews completed after 83% of identified births
**Maternal mortality ratio**	254.3	668.1	Unknown	206.2
**Health facilities available in control areas**	Public facilities: District Hospitals; Maternal and Child Welfare Centres; Upazilla Health Complexes. Private facilities: small-to-medium-size clinics; BRAC (NGO) facilities where deliveries do not take place; larger private hospitals with and without Comprehensive emergency obstetric care (CEmOC) facilities.	Public facilities: District Hospitals; Primary Health Centres (PHCs) in which deliveries can notionally take place but that are not usually equipped for CEmOC; Community Health Centres (CHCs) acting as referral centres for PHCs, covering a population of around 80,000 with EmOC facilities; district hospitals. Private and charitable facilities: medium-sized missionary hospitals with EmOC facilities.	Public facilities: three Primary Health Care Centres, three Health Posts and 24 Sub-Health Posts, none of which are equipped for CEmOC. These health facilities refer to the public Zonal Tertiary Hospital and various private providers in the district headquarters and nearby medical college, which have facilities for caesarean sections.	Public facilities: Municipal tertiary hospitals, general hospitals, and maternity homes. Private facilities: specialty hospitals, general hospitals, and maternity homes.

For peer review only

Table 2: Characteristics of institutional births and caesarean deliveries by location

	Births	Institutional births						Caesarean section births			
		All		Public facility		Private/charitable facility		Public facility		Private/charitable facility	
	(n)	(n)	(% all births)	(n)	(% institutional births)	(n)	(% institutional births)	(n)	(% deliveries in public facilities)	(n)	(% deliveries in private facilities)
**Bangladesh (rural)**	21,560	4,592	21%	2,053	45%	2,539	55%	589	29%	1,852	73%
**India (rural)**	8,541	1,816	21%	421	23%	1,395	77%	64	15%	66	5%
**India (urban)**	10,236	9,259	90%	5,489	59%	3,770	41%	811	15%	697	18%
**Nepal (rural)**	4,931	1,586	32%	1,424	90%	162	10%	201	14%	48	30%

* Nepal numbers weighted using women's probability of selection within each cluster, scaled to total number of institutional births. (Unweighted Nepal totals: 4,990 births and 1,586 institutional births)

For peer review only

Table 3: Mutually adjusted associations of type of facility of delivery and other selected determinants with caesarean section delivery, by location

	Bangladesh (rural) ORs (95% CIs)		India (rural) ORs (95% CIs)		India (urban) ORs (95% CIs)		Nepal (rural)* ORs (95% CIs)	
	Crude	Adjusted	Crude	Adjusted	Crude	Adjusted	Crude	Adjusted
**Health facility characteristics**								
Public health facility (ref)								
Private health facility	6.82 (5.96,7.81)	5.91 (5.15,6.78)	0.39 (0.25,0.61)	0.46 (0.29,0.73)	1.36 (1.21,1.52)	1.22 (1.09,1.38)	2.42 (1.48,3.94)	2.37 (1.62,3.44)
**Pregnancy and delivery characteristics**								
4+ antenatal care visits (fewer or none = ref)**		1.46 (1.26,1.69)		1.49 (0.96,2.32)		1.06 (0.93,1.21)		1.92 (1.43,2.58)
Birth order								
1 (ref)								
2		0.95 (0.80,1.13)		1.25 (0.80,1.95)		0.65 (0.56,0.75)		1.41 (1.02,1.94)
3		1.19 (0.94,1.52)		0.36 (0.15,0.87)		0.60 (0.50,0.71)		0.58 (0.37,0.92)
4+		1.17 (0.90,1.53)		0.36 (0.15,0.85)		0.39 (0.32,0.48)		1.00 (0.54,1.84)
Serious complications in pregnancy/delivery*** (no complications=ref)		0.87 (0.76,1.00)		1.77 (1.17,2.67)		1.71 (1.39,2.11)		4.87 (2.51,9.47)
Multiple birth (ref=single)		0.93 (0.65,1.32)		1.57 (0.52,4.75)		3.01 (2.14,4.23)		3.42 (1.77,6.61)
**Maternal characteristics**								
Age (years)								
15-24 (ref)								
25-34		1.12 (0.94,1.34)		1.81 (1.14,2.86)		1.45 (1.27,1.66)		1.52 (0.63,1.94)
35+		1.10 (0.77,1.58)		2.11 (0.63,7.07)		1.79 (1.27,2.53)		1.55 (0.55,1.47)

For peer review only - http://bmjopen.bmj.com/site/about/guidelines.xhtml

For peer review only

	Bangladesh (rural) ORs (95% CIs)		India (rural) ORs (95% CIs)		India (urban) ORs (95% CIs)		Nepal (rural)* ORs (95% CIs)	
	Crude	Adjusted	Crude	Adjusted	Crude	Adjusted	Crude	Adjusted
Maternal education****								
No formal education (ref)								
Primary education		1.07		0.98		1.13		0.90
		(0.83,1.38)		(0.39,2.47)		(0.85,1.49)		(0.59,1.39)
Secondary education		1.44		1.11		1.22		1.37
		(1.13,1.84)		(0.68,1.80)		(1.04,1.42)		(0.97,1.94)
Bachelor’s degree or higher		2.44		1.20		1.62		-
		(1.52,3.92)		(0.43,3.31)		(1.30,2.02)		-
Household wealth quintile								
1st (poorest, ref)								
2nd		1.31		1.61		1.14		1.11
		(1.06,1.63)		(0.69,3.75)		(0.77,1.68)		(0.63,1.94)
3rd		1.41		1.36		1.13		0.90
		(1.10,1.82)		(0.58,3.21)		(0.95,1.33)		(0.55,1.47)
4th (wealthiest)		1.36		2.16		1.50		0.99
		(1.09,1.70)		(0.87,5.33)		(1.27,1.78)		(0.52,1.90)
Year of delivery								
2004-2006 (ref: BD, IN)								-
2007-2009 (ref: NP rural)		0.90		0.90		1.14		-
		(0.74,1.10)		(0.61,1.33)		(1.09,1.38)		-
2010-2012		1.15		-		-		1.29
		(0.94,1.41)		-		-		(0.91,1.82)
N	4,592	4,592	1,816	1,816	9,259	9,259	1,586	1,586

* Nepal numbers weighted using women's probability of selection within each cluster, scaled to total number of institutional births.

** At least one visit with skilled provider

***Includes: symptoms of eclampsia (fits, seizures, convulsions, or unconsciousness during pregnancy or delivery); reduced or no fetal movement; labour lasting more than 24 hours.

****For the Nepal data, two respondents with bachelor's degrees (2 respondent total, 1 delivering in institution) were combined with respondents with secondary education.

All analyses additionally adjusted for survey design using fixed effect of stratum and random effect of cluster.

For peer review only

For peer review only - http://bmjopen.bmj.com/site/about/guidelines.xhtml

Table 4: Interactive associations between type of delivery facility, maternal education, and caesarean delivery, by location

	Bangladesh (rural)		India (rural)		India (urban)		Nepal (rural)*	
	(n)	AOR, 95% CI	(n)	AOR, 95% CI	(n)	AOR, 95% CI	(unweighted n)	AOR, 95% CI
Public facility - no maternal education (ref)	202	-	109	-	1,396	-	822	-
Public facility - primary education	448	1.07 (0.76,1.50)	17	0.92 (0.18,4.77)	357	0.88 (0.61,1.27)	196	1.04 (0.65,1.65)
Public facility - secondary education	202	1.63 (1.18,2.24)	604	1.09 (0.52,2.26)	920	1.1 (0.91,1.33)	413	1.45 (1.04,2.02)
Public facility - bachelor's degree	448	4.58 (2.24,9.38)	100	1.55 (0.41,5.89)	180	1.06 (0.76,1.47)	-	- -
Private facility - no education	332	7.24 (4.82,10.87)	109	0.46 (0.21,1.01)	1396	0.91 (0.70,1.19)	100	2.83 (1.73,4.61)
Private facility - primary education	578	7.58 (5.36,10.71)	17	0.47 (0.14,1.58)	357	1.52 (0.99,2.33)	16	0.86 (0.14,5.21)
Private facility - secondary education	1,102	9.03 (6.57,12.41)	274	0.52 (0.24,1.12)	3,382	1.3 (1.06,1.60)	39	3.01 (1.71,5.30)
Private facility - bachelor's degree	41	11.24 (6.37,19.85)	21	0.4 (0.08,2.00)	354	2.1 (1.61,2.75)	-	- -
N	3,353		1,251		8,342		1586	
p-value**	0.021		0.792		0.001		0.394	

All results adjusted for number of antenatal care visits, parity, medical indication for caesarean, multiple birth, maternal age (10-yr group), household assets, stratum, and cluster (random effect).
* Nepal numbers weighted using women's probability of selection within each cluster, scaled to total number of institutional births.

** p-value from -2 log-likelihood test comparing nested models with and without interaction terms

For peer review only - http://bmjopen.bmj.com/site/about/guidelines.xhtml

"Prevalence and determinants of caesarean section in private and public health facilities in underserved South Asian communities: cross-sectional analysis of data from Bangladesh, India, and Nepal"

Supplemental table

Supplemental table 1. Characteristics of institutional deliveries by characteristics of the mother, the delivery facility, and location

For peer review only

	Bangladesh (rural)				India (rural)				India (urban)				Nepal (rural)			
	Not caesarean	Caesarean	Total		Not caesarean	Caesarean	Total		Not caesarean	Caesarean	Total		Not caesarean	Caesarean	Total	
	(%)	(%)	(n)	(%)	(%)	(%)	(n)	(%)	(%)	(%)	(n)	(%)	(%)	(%)	(n)	(%)
**Health facility characteristics**																
Public health facility	68.1	24.1	2053	44.7	21.2	49.2	421	23.2	60.4	53.8	5489	59.3	91.5	80.7	1424	89.8
Private/charitable health facility	31.9	75.9	2539	55.3	78.8	50.8	1395	76.9	39.6	46.2	3770	40.7	8.5	19.3	162	10.2
**Pregnancy and delivery characteristics**																
4+ antenatal care visits*	25.2	38.8	1491	67.5	47.1	56.2	867	52.3	73.4	74.2	6810	26.4	41.3	53.6	686	43.2
Birth order																
1	47.1	47.4	2170	47.3	51.6	55.4	942	51.9	29.7	41.6	2932	31.7	50.1	46.7	786	49.6
2	25.9	26.1	1194	26	23	32.3	430	23.7	27.9	26.1	2554	27.6	22.5	27.1	368	23.2
3	12.4	13.8	603	13.1	11.7	5.4	204	11.2	19.4	17.6	1769	19.1	14.9	10.3	225	14.2
4+	14.6	12.7	625	13.6	13.7	6.9	240	13.2	23	14.7	2004	21.6	12.5	15.9	207	13
Serious complications in pregnancy/delivery**	53.4	43.5	2212	48.2	29.2	44.6	550	30.3	6.1	9.2	608	6.6	21.2	38.5	379	23.9
Multiple birth	3.6	3.9	173	3.8	2.1	3.1	40	2.2	1.4	3.6	163	1.8	2.2	7.8	48	3.1
**Maternal socio-demographic characteristics**																
Maternal age (years)																
15-24	59.8	55.8	2650	57.7	63.6	60	1150	63.3	53.1	51.5	4894	52.9	71.6	66.3	1123	70.8
25-34	34.4	39.5	1705	37.1	32.7	36.9	600	33	43.6	45.3	4060	43.8	26.4	31	431	27.2
35+	5.8	4.6	237	5.2	3.7	3.1	66	3.6	3.3	3.2	305	3.3	1.9	2.7	33	2.1
Maternal education																
No formal education	15.2	8.3	531	11.6	40.1	28.5	713	39.3	26.3	18.5	2316	25	58.4	57.2	924	58.2
Primary education	27.3	17.9	1025	22.3	6.6	4.6	117	6.4	6	4.9	537	5.8	13.6	10.8	209	13.2
Secondary education	55.7	68.6	2872	62.5	50.2	62.3	927	51	59.6	61.9	5550	59.9	28	32	453	28.6
Bachelor's degree or higher***	1.7	5.2	164	3.6	3.1	4.6	59	3.2	8.2	14.7	856	9.2	-	-	-	-

Household wealth quintile																
1st (poorest)	23	10.4	749	16.3	12.6	5.4	220	12.1	24.1	17.2	2125	23	14.2	13.4	223	14.1
2nd	32.4	31.5	1465	31.9	30	31.5	547	30.1	3	2.3	268	2.9	22	22.4	350	22.1
3rd	14	15.2	673	14.7	39.9	32.3	715	39.4	35.9	31.3	3254	35.1	33.9	30.5	529	33.4
4th (wealthiest)	30.5	42.9	1705	37.1	17.4	30.8	334	18.4	37	49.3	3612	39	29.9	33.6	483	30.5
**Year of delivery**																
2004-2006	19.2	15.2	782	17	36.7	40.8	672	37	46.4	42.2	4237	45.8	-	-	-	-
2007-2009	44.7	38.7	1907	41.5	63.3	59.2	1144	63	53.6	57.8	5022	54.2	45.1	45.7	717	45.2
2010-2012	36.1	46.1	1903	41.4	-	-	-	-	-	-	-	-	54.9	54.3	869	54.8
Total (n)	2151	2441	4592	100	1686	130	4592	100	7751	1508	9259	100	734	135	869	100

* Nepal numbers weighted using women's probability of selection within each cluster, scaled to total number of institutional births.

** At least one visit with skilled provider

***Includes: symptoms of pre-eclampsia (swollen face or limbs or blurred vision [not asked in Nepal]), eclampsia (fits, seizures, convulsions, or unconsciousness during pregnancy or delivery); haemorrhaging during delivery, labour lasting more than 24 hours.

****For the Nepal data, secondary and bachelor's (2 respondent total, 1 delivering in institution) were combined.

For peer review only

STROBE 2007 (v4) checklist of items to be included in reports of observational studies in epidemiology*
Checklist for cohort, case-control, and cross-sectional studies (combined)

Section/Topic	Item #	Recommendation	Reported on page #
**Title and abstract**	1	(*a*) Indicate the study's design with a commonly used term in the title or the abstract	1
		(*b*) Provide in the abstract an informative and balanced summary of what was done and what was found	2
**Introduction**			
Background/rationale	2	Explain the scientific background and rationale for the investigation being reported	4
Objectives	3	State specific objectives, including any pre-specified hypotheses	4
**Methods**			
Study design	4	Present key elements of study design early in the paper	1, 5-6
Setting	5	Describe the setting, locations, and relevant dates, including periods of recruitment, exposure, follow-up, and data collection	5 and Table 1
Participants	6	(*a*) *Cohort study*—Give the eligibility criteria, and the sources and methods of selection of participants. Describe methods of follow-up<br>*Case-control study*—Give the eligibility criteria, and the sources and methods of case ascertainment and control selection. Give the rationale for the choice of cases and controls<br>*Cross-sectional study*—Give the eligibility criteria, and the sources and methods of selection of participants	6
		(*b*) *Cohort study*—For matched studies, give matching criteria and number of exposed and unexposed<br>*Case-control study*—For matched studies, give matching criteria and the number of controls per case	
Variables	7	Clearly define all outcomes, exposures, predictors, potential confounders, and effect modifiers. Give diagnostic criteria, if applicable	7
Data sources/ measurement	8*	For each variable of interest, give sources of data and details of methods of assessment (measurement). Describe comparability of assessment methods if there is more than one group	7
Bias	9	Describe any efforts to address potential sources of bias	7-8
Study size	10	Explain how the study size was arrived at	6
Quantitative variables	11	Explain how quantitative variables were handled in the analyses. If applicable, describe which groupings were chosen and why	7
Statistical methods	12	(*a*) Describe all statistical methods, including those used to control for confounding	7-8
		(*b*) Describe any methods used to examine subgroups and interactions	Table 4
		(*c*) Explain how missing data were addressed	7
		(*d*) *Cohort study*—If applicable, explain how loss to follow-up was addressed<br>*Case-control study*—If applicable, explain how matching of cases and controls was addressed	

For peer review only

		Cross-sectional study—If applicable, describe analytical methods taking account of sampling strategy	
		(*e*) Describe any sensitivity analyses	N/A
**Results**			
Participants	13*	(a) Report numbers of individuals at each stage of study—eg numbers potentially eligible, examined for eligibility, confirmed eligible, included in the study, completing follow-up, and analysed	7, 8
		(b) Give reasons for non-participation at each stage	7, 8
		(c) Consider use of a flow diagram	Not used
Descriptive data	14*	(a) Give characteristics of study participants (eg demographic, clinical, social) and information on exposures and potential confounders	Supplemental Table 1
		(b) Indicate number of participants with missing data for each variable of interest	
		(c) *Cohort study*—Summarise follow-up time (eg, average and total amount)	
Outcome data	15*	*Cohort study*—Report numbers of outcome events or summary measures over time	
		Case-control study—Report numbers in each exposure category, or summary measures of exposure	
		Cross-sectional study—Report numbers of outcome events or summary measures	Table 3
Main results	16	(*a*) Give unadjusted estimates and, if applicable, confounder-adjusted estimates and their precision (eg, 95% confidence interval). Make clear which confounders were adjusted for and why they were included	Table 3
		(*b*) Report category boundaries when continuous variables were categorized	
		(*c*) If relevant, consider translating estimates of relative risk into absolute risk for a meaningful time period	
Other analyses	17	Report other analyses done—eg analyses of subgroups and interactions, and sensitivity analyses	Table 4
**Discussion**			
Key results	18	Summarise key results with reference to study objectives	10
Limitations	19	Discuss limitations of the study, taking into account sources of potential bias or imprecision. Discuss both direction and magnitude of any potential bias	12
Interpretation	20	Give a cautious overall interpretation of results considering objectives, limitations, multiplicity of analyses, results from similar studies, and other relevant evidence	10-1
Generalisability	21	Discuss the generalisability (external validity) of the study results	12
**Other information**			
Funding	22	Give the source of funding and the role of the funders for the present study and, if applicable, for the original study on which the present article is based	13

*Give information separately for cases and controls in case-control studies and, if applicable, for exposed and unexposed groups in cohort and cross-sectional studies.

Note: An Explanation and Elaboration article discusses each checklist item and gives methodological background and published examples of transparent reporting. The STROBE checklist is best used in conjunction with this article (freely available on the Web sites of PLoS Medicine at http://www.plosmedicine.org/, Annals of Internal Medicine at http://www.annals.org/, and Epidemiology at http://www.epidem.com/). Information on the STROBE Initiative is available at www.strobe-statement.org.

For peer review only

BMJ Open

Prevalence and determinants of caesarean section in private and public health facilities in underserved South Asian communities: cross-sectional analysis of data from Bangladesh, India, and Nepal

Journal:	*BMJ Open*
Manuscript ID:	bmjopen-2014-005982.R1
Article Type:	Research
Date Submitted by the Author:	04-Sep-2014
Complete List of Authors:	Neuman, Melissa; UCL Institute for Global Health,<br>Alcock, Glyn; UCL Institute for Global Health,<br>Azad, Kishwar; Diabetic Association of Bangladesh, Perinatal Care Project<br>Kuddus, Abdul; Diabetic Association of Bangladesh, Perinatal Care Project<br>Osrin, David; UCL Institute for Global Health,<br>Shah More, Neena; Society for Nutrition, Education and Health Action (SNEHA),<br>Nair, Nirmala; Ekjut,<br>Tripathy, Prasanta; Ekjut,<br>Sikorski, Catherine; UCL Institute for Global Health,<br>Saville, Naomi; UCL Institute for Global Health,<br>Sen, Aman; Mother and Infant Research Activities (MIRA),<br>Colbourn, Timothy; UCL Institute for Global Health,<br>Houweling, Tanja; Erasmus MC University Medical Center, Department of Public Health<br>Seward, Nadine; UCL Institute for Global Health,<br>Manandhar, Dharma; Mother and Infant Research Activities (MIRA),<br>Shrestha, Bhim; Mother and Infant Research Activities (MIRA),<br>Costello, Anthony; UCL Institute for Global Health,<br>Prost, Audrey; UCL Institute for Global Health,
**Primary Subject Heading**:	Global health
Secondary Subject Heading:	Epidemiology, Obstetrics and gynaecology
Keywords:	Epidemiology < TROPICAL MEDICINE, OBSTETRICS, PUBLIC HEALTH

SCHOLARONE™ Manuscripts

Prevalence and determinants of caesarean section in private and public health facilities in underserved South Asian communities: cross-sectional analysis of data from Bangladesh, India, and Nepal

Melissa Neuman[1], Glyn Alcock[1], Kishwar Azad[2], Abdul Kuddus[2], David Osrin[1], Neena Shah More[3], Nirmala Nair[4], Prasanta Tripathy[4], Catherine Sikorski[1], Naomi Saville[1], Aman Sen[5], Tim Colbourn[1], Tanja AJ Houweling[6], Nadine Seward[1], Dharma S Manandhar[5], Bhim P Shrestha[5], Anthony Costello[1] and Audrey Prost[1]

Author affiliations:

[1] Institute for Global Health, University College London, 30 Guilford Street, London WC1N 1EH, UK

[2] Perinatal Care Project, Diabetic Association of Bangladesh, 122 Kazi Nazrul Islam Avenue, Dhaka 1000, Bangladesh

[3] Society for Nutrition, Education and Health Action (SNEHA), Urban Health Centre, Shahunagar, Dharavi, Mumbai 400017, Maharashtra, India

[4] Ekjut, Plot 556B, Potka, Chakradharpur, West Singhbhum, Jharkhand, India

[5] Mother and Infant Research Activities (MIRA), YB Bhavan, Thapathali, GPO Box 921, Kathmandu, Nepal

[6] Department of Public Health, Erasmus MC University Medical Center Rotterdam, P.O. Box 2040, 3000 CA Rotterdam, The Netherlands

Proposed running head: Caesarean sections in private vs. public health facilities in South Asia

Word count: 4393

Figures and tables: 0 figures and 4 tables

For peer review only

For peer review only - http://bmjopen.bmj.com/site/about/guidelines.xhtml

ABSTRACT (Max: 300 words; Current: 298)

Objectives: To describe the prevalence and determinants of births by caesarean section in private and public health facilities in underserved communities in South Asia.

Design: Secondary, cross-sectional analysis.

Setting: 81 community-based geographical clusters in four locations in Bangladesh, India and Nepal (three rural, one urban).

Participants: 45,327 births (2005 to 2012).

Outcome measures: proportion of caesarean section delivery by location and type of facility; determinants of caesarean section delivery by location.

Results: Institutional delivery rates varied widely between settings, from 21% in rural India to 90% in informal settlements in urban India. The proportion of births by caesarean section in private facilities versus public facilities was 73% vs. 29% in Bangladesh, 30% vs. 14% in rural Nepal, 18% vs. 15% in informal Indian urban settlements, and 5% vs. 15% in rural India. The odds of caesarean section were greater in private facilities than in public facilities in three of four study locations, even when adjusted for pregnancy and delivery characteristics, maternal characteristics, and year of delivery (Bangladesh AOR: 5.91, 95% CI: 5.15-6.78; Nepal AOR: 2.37, 95% CI: 1.62-3.44; informal settlements in urban India AOR: 1.22, 95% CI: 1.09-1.38). We found significant interactive associations between maternal education and private facility delivery in two of four locations (p=0.025 in rural Bangladesh and p<0.001 in informal settlements in urban India), with highly educated women particularly likely to deliver by caesarean in private facilities.

Conclusions:

Our results lend support to the hypothesis that increased caesarean section rates in these South Asian countries may be driven in part by the private sector. They also suggest that preferences for caesarean delivery may be stronger among highly educated women, and that individual- and provider-level factors interact in driving caesarean rates upwards. Increasing rates of caesarean section in the private sector require close monitoring and action.

For peer review only

For peer review only - http://bmjopen.bmj.com/site/about/guidelines.xhtml

Article summary

Article focus

- Caesarean sections are on the rise in South Asia. Research suggests that private health facilities may be contributing to this by conducting caesareans over and above clinical need.
- Literature from other settings indicates that increases in caesarean sections are shaped by both supply and demand pressures: providers often have financial incentives to intervene surgically, and women of higher socio-economic status are also more likely to want caesareans.
- This study examined the prevalence and determinants of caesarean sections in private and charitable facilities compared with public health facilities for over 45,000 births in underserved communities of rural Nepal, rural Bangladesh, rural India, and informal settlements in urban India.

Key messages

- Caesarean sections were more common in private than in public health facilities in three of four study locations (rural Nepal, rural Bangladesh, and informal settlements in urban India).
- We found significant interactive associations between maternal education and caesarean delivery in Bangladesh (both private and public facilities) and in informal Indian urban settlements (private facilities only).
- Future research should monitor caesarean section rates in the private sector in South Asia and subsequent maternal and neonatal health outcomes

Strengths and limitations

- This study had a large sample size and focused on underserved communities, which are a priority for public health interventions in South Asia.
- Our data were not nationally or sub-nationally representative, which limits the generalisability of our findings. Other limitations included small denominators and missing data for some variables. We did not have data on some known predictors of caesarean section, which would have enhanced the completeness of our determinants analysis.

For peer review only

For peer review only - http://bmjopen.bmj.com/site/about/guidelines.xhtml

INTRODUCTION

Access to comprehensive emergency obstetric care, including caesarean section, is key to preventing the estimated 287,000 maternal and 2.9 million neonatal deaths that occur worldwide every year.[1 2] Although debate continues about how to quantify the need for life-saving obstetric surgery, a 1985 World Health Organization (WHO) report suggested that the optimal population range for caesarean section rates is between 5% and 15%, and this endures as a reference.[3 4] Caesarean section rates are increasing worldwide, albeit unequally: a recent analysis of Demographic and Health Survey (DHS) data in 26 South Asian and sub-Saharan African countries found that rates were highest among the 'urban rich' in all countries, and lowest among the 'rural poor' in 18.[5] In all countries, fewer than 5% of mothers in the poorest wealth quintile delivered by caesarean.[6]

Caesarean sections conducted without clinical need can have adverse consequences for mothers and children. A 2008 WHO survey of 373 facilities across 24 countries found that unnecessary caesareans were associated with an increased risk of maternal mortality and serious outcomes for mothers and newborn infants, compared with spontaneous vaginal delivery.[7] Recent ecological analyses also highlighted strong associations between caesarean delivery and increased neonatal mortality in countries with low and medium caesarean section rates.[8] Unnecessary caesareans lead to considerable costs for families and health systems: an estimated 6.2 million unnecessary procedures were performed in 2008, costing approximately US$ 2.32 billion. [9]

Several South Asian countries have recorded substantial increases in caesarean section rates over the past decade. In Bangladesh, rates rose from 2% (2000) to 17% (2011); in India, from 3% (1992) to 11 % (2006); and in Nepal, from 1% (2000) to 5% (2011).[10-5] Several studies in these countries have raised concerns about high caesarean rates in private facilities, and a recent DHS analysis speculated that national increases in caesarean section rates in South Asian countries could be driven in part by higher rates among deliveries in private sector facilities[.6 16-18] Literature from other settings indicates that increases in caesarean sections are shaped by both supply and demand pressures: providers often have financial incentives to intervene surgically, and women of higher socio-economic status are also more likely to desire caesareans.[19 20]

There are few large, recent community-based studies from South Asia quantifying differences in caesarean section rates between public and private facilities. Such studies are necessary in order to examine whether increases in caesareans in these settings are indeed likely to be driven by the private sector, demand from wealthier and more educated mothers, or a combination of the two. We conducted a cross-sectional analysis of data from Bangladesh, India, and Nepal to explore the prevalence and determinants of caesarean section delivery by type of facility and location, and to examine the

For peer review only - http://bmjopen.bmj.com/site/about/guidelines.xhtml

interactions between maternal education and caesarean section delivery in private and public facilities.

METHODS

Study populations

We used data collected through vital events surveillance systems established during four cluster-randomised controlled trials conducted between 2005 and 2011. The trials were done with communities that can be considered socio-economically disadvantaged: in Bangladesh and Nepal, they took place in four rural, underserved districts (Bogra, Maulvibazaar, and Faridpur in Bangladesh, and the Terai district of Dhanusha, Nepal); in rural India, most participants were from Scheduled Tribes in two states of eastern India (Jharkhand and Odisha); in urban India, data came from informal settlements (slums) in Mumbai. **Table 1** describes the characteristics of each study and its population, including the background maternal mortality ratio and types of facilities present in the study areas. The original trials were designed to evaluate the impact of participatory women's groups on maternal and neonatal health outcomes.[21-25] We used data from the control areas of these trials only, because the women's group intervention led to changes in mortality and practices in several locations.[26]

Health system contexts

All three study countries have experienced substantial increases in institutional delivery rates over the last two decades: births in health facilities increased from 4% (1993) to 29% (2011) in Bangladesh; from 26% (1992-3) to 47% (2007-8) in India; and from 8% (1996) to 35% (2011) in Nepal.[10-15] All three countries have implemented incentive schemes to promote institutional delivery, though these have varying coverage. In 2010, Bangladesh's pilot maternity voucher scheme reached an estimated 10.4 million people across 31 sub-districts, around 7% of the country's population.[27 28] Mothers receive a cash incentive for antenatal care and delivery in a public or private facility, or at home with a skilled birth attendant. Both government and private facility staff also receive cash incentives, including 3000 Bangladeshi Taka (US $38.5) for a caesarean section and 300 Taka for a normal delivery.[28] This maternity incentive scheme was operational in two of the three districts (Faridpur and Maulvibazaar) covered by our study. All three districts had public facilities including District Hospitals, Maternal and Child Welfare Centres, and Upazilla Health Complexes. Private facilities included a number of small-to-medium-size clinics, BRAC (NGO) facilities, and larger private hospitals.

In India, the *Janani Suraksha Yojana* (JSY) maternity incentive scheme entitles women in rural areas of high focus states, including those in this study, to 1400 Indian Rupees ($22.4) after delivering in a government or accredited private health facility. Local community health volunteers called Accredited Social Health Activists (ASHAs) also receive 600 Indian Rupees ($9.6) for identifying pregnant women and helping them get to a health facility.[29] Although the rural Indian data included in this study were

For peer review only - http://bmjopen.bmj.com/site/about/guidelines.xhtml

collected between 2005 and 2008, the JSY was only operational in the study areas from 2008 onwards and its impact is unlikely to be reflected here. The Indian urban area included in our study spanned informal settlements with a wealth of public and private providers.[30] Mothers with Below Poverty Line cards in such areas are eligible for JSY and can receive Rs 500 towards the costs of delivering in a health facility.[31] Nepal began a safe delivery incentive scheme in 2005 and free deliveries have been available in government facilities since 2009 through the *Aama Surakshya* programme.[32] Dhanusha district, from which data for this study come, has one zonal tertiary hospital and a private medical college hospital equipped for caesarean sections, and a variety of small public and private health facilities without comprehensive obstetric care.

Data collection

All data were collected using surveillance systems to monitor births and deaths prospectively.[21-25] In all study locations, a female community-based key informant reported births and deaths in her area, which covered a population ranging from 250 to 350. A trained interviewer then verified these reports and paid the informant an incentive for each correct identification. In Bangladesh, rural and urban India, the interviewer administered a structured questionnaire to all eligible mothers around six weeks after delivery; in Nepal, all births in the study area were registered, and interviews were conducted on all births in small clusters and on a random sample of 10 births per month in the larger clusters. In each study location, mothers were interviewed using a questionnaire to collect information about events in the antenatal, delivery and postnatal periods. In each trial and in this subsequent study, participants were women of reproductive age (15-49) who delivered in the study area during the data collection period, and who consented to be interviewed six weeks after delivery, and their infants.

Study sample

The initial sample for this analysis included 46,393 births in Mumbai, rural India, rural Bangladesh, or rural Nepal, of whom 17,565 (38%) were delivered in health care facilities. We excluded the 2348 births occurring outside Mumbai, because we collected limited information on delivery location for these. Additionally, 223 deliveries had missing data on form of delivery (caesarean or vaginal), 602 had missing or implausible values for mother's age, and 98 were missing information on other maternal characteristics (educational attainment, household assets, number of prior pregnancies, number of antenatal visits, severe problems in pregnancy or delivery). 143 births had missing or incomplete information on place of delivery. The final analytic sample included 45,327 births (97.7% of the initial sample).

For peer review only - http://bmjopen.bmj.com/site/about/guidelines.xhtml

Measures used

The primary outcome in these analyses was caesarean section delivery, identified by self-report from the mother or another household member around six weeks after giving birth. The main covariate of interest was the type of delivery facility, coded as public (e.g. district hospitals), private (individual private clinics or hospitals), or NGO/charitable (e.g. Christian missionary hospitals in rural India; BRAC clinics in Bangladesh). We grouped private and NGO/charitable facilities together due to the small number of births in NGO/charitable facilities (6% of institutional deliveries in Bangladesh, 7% in rural India, and 0 in informal settlements in urban India and rural Nepal; data on request). While we refer to all results from Mumbai as being from 'informal settlements', is worth noting that the health facilities used by women in informal settlements for delivery are not always in the settlements themselves: women often travel to other parts of the city to seek care with a recommended or preferred provider, usually private. Additional covariates in the models included measures of the characteristics of the pregnancy and delivery, maternal socio-demographic characteristics, and the location in which the delivery occurred. We created an indicator variable to identify women experiencing serious problems during pregnancy or delivery, with women reporting symptoms of pre-eclampsia (blurred vision or swelling of face and hands), symptoms of eclampsia (fits or seizures during pregnancy or delivery), hæmorrhage during delivery, or labour lasting more than 24 hours considered to have serious complications. Other characteristics of the pregnancy and delivery considered in the analysis included full utilisation of antenatal care, number of prior pregnancies, and multiple pregnancy. Full utilization of antenatal care was defined as four or more visits to antenatal care, with at least one visit to a skilled provider. Number of pregnancies was entered as an ordered categorical variable, with categories of one (first), two, three, or four or more pregnancies.

Maternal socio-demographic characteristics included in the models were: mother's age at delivery, her educational attainment, and household assets. Mother's age was entered into the model as a categorical measure in 10-year groups. Educational attainment was entered as a categorical variable using the following categories: no formal education, primary education, secondary education, or bachelor's degree or higher. To develop an asset index, we used polychoric factor analysis on data on common assets and amenities found in the mother's household, and grouped the resulting factor scores into quartiles.[33] Assets and amenities included electricity, radio or cassette player, electric fan (Bangladesh and India only), television, refrigerator (Bangladesh and India only), telephone (Bangladesh and Nepal only), generator (India only), and bicycle. All models were additionally adjusted for location (Bangladesh, India, Nepal), and year of interview in three-year groups. The data were collected in a stratified, cluster-sampled survey, and we accounted for survey design in the analysis using a fixed effect for stratum and a random effect for cluster.

For peer review only - http://bmjopen.bmj.com/site/about/guidelines.xhtml

Statistical analysis

We used frequencies to describe caesarean section rates by delivery location at each site. We used the Generalized Linear Latent And Mixed Models (GLLAMM) procedure in Stata 13.1, with adaptive quadrature for binary outcomes, to estimate the crude association between type of delivery facility and caesarean section.[34 35] We identified other maternal, pregnancy, and delivery characteristics potentially associated with caesarean section using existing literature, especially studies resulting from the WHO multi-country surveys, and entered these in adjusted models to explore how they modified the association between type of delivery facility and caesarean section, and their individual association with caesarean section. Some South Asian and Latin American studies have detected a strong association between maternal education and caesarean delivery. [7 36 37] We fitted models including indicator variables for each group of mothers by education and type of delivery facility to explore differences in the strength of association between caesarean delivery and private facility by mother's education. To account for the sampling procedure used in rural Nepal, models were adjusted using pweights (probability of selection within cluster); these weights were rescaled to reflect the total number of institutional deliveries.

Ethical approval

The trials that provided data for this study received ethical approval from the following committees: the ethics committee of the Diabetic Association of Bangladesh (Perinatal Care Project, Bangladesh Diabetes Somity, BADAS); an independent ethics committee in Jamshedpur, Jharkhand, India; the Nepal Health Research Council; the Municipal Corporation of Greater Mumbai and the Independent Ethics Committee for Research on Human Subjects (Mumbai, India); the ethics committee of the Institute of Child Health, University College London.

RESULTS

Table 2 describes the characteristics of institutional births and caesarean deliveries by location. We analysed data for 45,327 births: 21,560 in rural Bangladesh, 8,541 in rural India, 10,236 in informal settlements in urban India, and 4,931 in rural Nepal. The proportion of women delivering in health facilities varied widely between locations, from 90% in urban India to 21% in rural India. There were also large variations in the proportion of women delivering in private/charitable facilities rather than public facilities, with the highest (77%) in rural India and the lowest (10%) in rural Nepal. The proportion of women giving birth by caesarean section in private rather than public facilities also varied widely between settings. In Bangladesh, only 21% of women delivered in a health facility, around half of them in the private/charitable sector, but 73% of private facility births were by caesarean section. In rural Nepal, 30% of the 162 private facility births were by caesarean section. In informal settlements of Mumbai, 15% of public facility deliveries were caesareans, compared with 18% of private facility

For peer review only - http://bmjopen.bmj.com/site/about/guidelines.xhtml

For peer review only

deliveries. In rural India, caesarean sections were more commonly performed in public health facilities than private or charitable facilities (15% vs. 5%). **Supplemental table 1** describes the characteristics of institutional deliveries (caesarean or non-caesarean) according to type of delivery facility (private or public), maternal socio-economic and socio-demographic characteristics, pregnancy and delivery characteristics, and year of delivery.

Table 3 shows crude and adjusted measures of association between type of delivery facility (private or public) and caesarean section for each location. Delivering in a private health facility was associated with an increased odds of caesarean section in all but one location (rural India). The relative odds of a caesarean in a private facility were greatest in Bangladesh (OR: 6.82, 95% CI: 5.96, 7.81), followed by Nepal (OR: 2.42, 95% CI: 1.48, 3.94) and informal settlements in urban India (OR 1.36, 95% CI: 1.21, 1.52). These associations persisted and were only mildly attenuated when adjusted for maternal characteristics, pregnancy and delivery characteristics, and year of delivery (Bangladesh AOR: 5.91, 95% CI: 5.15, 6.78; Nepal AOR: 2.37, 95% CI: 1.62, 3.44; informal settlements in urban India AOR: 1.22, 95% CI: 1.09, 1.38).

Women who had four or more antenatal check-ups were significantly more likely to have a caesarean delivery in rural Bangladesh and Nepal, with positive but non-significant trends in all other locations. Parity was not associated with caesarean delivery in any location. Having a serious health complication during pregnancy and delivery was associated with caesarean delivery in all locations except rural Bangladesh, where we observed a negative association (AOR: 0.87, 95% CI: 0.76, 1.00). Multiple birth was associated with an increased odds of caesarean section only in informal settlements in urban India and in rural Nepal. Higher maternal age was only associated with an increased odds of caesarean section in informal settlements in urban India. Maternal education was only associated with an increased odds of caesarean delivery in rural Bangladesh and informal settlements in urban India, with mothers with secondary education or Bachelor's degrees having higher odds (AOR: 1.44, 95% CI: 1.13, 1.84 and AOR 2.44, 95% CI: 1.52, 3.92 for Bangladesh and AOR: 1.22, 95% CI: 1.04, 1.42 and 1.62, 95% CI: 1.30, 2.02 for informal settlements in urban India, respectively). There were significant positive associations only in rural Bangladesh, with small increases in odds of caesarean section for unit increase in wealth quartile (AOR comparing wealthiest to poorest group in Bangladesh: 1.36, 95% CI: 1.09,1.70), and in informal settlements in urban India, for the wealthiest quartile (AOR: 1.50, 95% CI: 1.27, 1.78).

We found interactive associations between maternal education and private facility delivery in two of four sites (p=0.021 in rural Bangladesh and p<0.001 in informal settlements in urban India; **table 4**). In Bangladesh, there was a pronounced positive educational gradient in caesarean delivery within those who delivered in private facilities, as well as a clear positive association between private facility delivery

For peer review only - http://bmjopen.bmj.com/site/about/guidelines.xhtml

For peer review only

and caesarean delivery. Women with bachelor's degrees delivering in private facilities had 11 times greater odds of delivering by caesarean (AOR 11.09, 95% CI: 6.28, 19.57) than did women with no formal education delivering in public facilities. Women with no formal education delivering in private facilities had nearly seven times the odds of caesarean compared with the reference group (AOR 6.94, 95% CI: 4.64, 10.39). A similar positive gradient was seen among women delivering in public facilities, with women with bachelor's education having four times greater odds of caesarean delivery in public facilities than women with no formal education (AOR 4.55, 95% CI: 2.22, 9.33). There was also an educational gradient among women in informal settlements in urban India delivering in private facilities. Educated mothers delivering in private facilities were more likely to deliver by caesarean than were mothers in other groups (AOR 2.10; 95% CI: 1.61, 2.75). However, there was no apparent gradient by educational attainment among women delivering in public facilities.

DISCUSSION

Our analysis of data from over 45,000 births confirms the findings of other studies identifying differences in caesarean rates between public and private facilities, and suggests that, even in underserved areas in South Asia, caesareans without medical indication are of concern. In three of four locations, rates of caesarean section were higher in private/charitable facilities than in public facilities. The findings from Bangladesh are particularly noteworthy as they show much greater odds of caesarean section in private facilities, concurring with previous analyses.[37] This was also the only location where serious complications in pregnancy and delivery were not associated with caesarean delivery, suggesting that obstetric surgery was performed over and above clinical need.

Our findings confirm the results of earlier studies of the prevalence of caesarean delivery in South Asia, and indicate that high rates can be found in underserved rural areas. In India, a recent analysis of 2010-11 Annual Health Survey (AHS) data from 284 districts in nine States, including Jharkhand, found that the median caesarean section rate in the private sector was 28%, compared with 5% in the public sector.[38] There appear to be strong financial incentives for surgical procedures in the private sector.[39] In a rural, largely indigenous part of eastern India where more mothers delivered in private/charitable facilities than in public facilities, more caesarean sections were performed in the public sector. In this particular setting, our field experience suggests that only women with serious complications would go to a facility and have a caesarean section, especially as JSY was not yet available in the study areas at the time of data collection. Such women are likely to have experienced multiple referrals from either ill-equipped public or private facilities not wanting to take the risk of admission. It is however possible that preference for the private sector has changed in Jharkhand since the advent of JSY, and further

For peer review only - http://bmjopen.bmj.com/site/about/guidelines.xhtml

disaggregated analyses of Annual Health Survey data would allow a more contemporary exploration of State-level variations in caesarean section rates between public and private sectors.

In Mumbai slums with a high uptake of institutional births and a 60/40 split in favour of the public sector for delivery care, we found no difference in caesarean section rates between the two sectors, which is somewhat surprising given the high rates of caesareans observed in the private sector in nine other states of India (excluding Maharashtra).[38] At least two scenarios may be relevant in this setting. First, public sector hospitals are able to provide caesarean section, while smaller private maternity homes may not be able to. Second, the costs of caesarean section borne by private sector providers are higher in Mumbai, and families may be unwilling to bear these additional costs. This provides motivation for private providers not to provide caesareans, and makes it likely that women experiencing complications move into the public sector. The variety of facility options in an urban environment means that mothers are able to choose between different types of facilities, while in rural areas only one provider may be accessible.

Analyses using the most recent Bangladesh DHS found that, in 2011, three in five facility births were delivered by caesarean section. This reflects a historical trend: in 2001-2003, nearly half of deliveries in private facilities in Bangladesh were already by caesarean section.[18] Our data from rural, socio-economically disadvantaged communities in three districts of Bangladesh confirm these population-level findings, but also suggest that high caesarean section rates in private facilities are not merely an issue for wealthy urban mothers. Although some research from Bangladesh suggests that mothers may have a preference for caesarean delivery because of fear of labour pain or a desire to select an auspicious date for the birth, other studies also highlight women's fears of caesarean section and their distrust of health providers who recommend them, mainly because of the high costs associated with the procedure.[37 40 41] A qualitative study involving twenty women who had experienced obstetric complications in Matlab in 2008-9 found that most of the fourteen women who had undergone caesareans had spent over Tk 14,999 (US$ 217) on the procedure, which was approximately one-third of GDP per capita at the time.[41] The lack of association between caesareans and complications in pregnancy or delivery and multiple births, coupled with the high financial incentives given to providers for performing caesarean sections, further suggest that obstetric surgery is being used over and above clinical need. A possible explanation for the significant interactive associations between maternal education and caesarean delivery in both public and private facilities in Bangladesh is that well-educated women may be delivering in more expensive or highly rated institutions, which may in turn be more likely to do caesarean sections for financial reasons. Our own experience also suggests that such facilities have additional incentives to encourage caesareans in order to allow junior doctors to practice their surgical skills.

For peer review only - http://bmjopen.bmj.com/site/about/guidelines.xhtml

For peer review only

In our sample of institutional deliveries from rural Nepal, 5% of all births were by caesarean, which is similar to the national average: the 2011 Nepal DHS found an overall national caesarean section rate of 4.6%, with sections more commonly performed for births to highly educated mothers (13%) and mothers in the highest wealth quintile (14%). [26]

A 2010 analysis of DHS data examining the role of the private sector in maternity care in 16 countries found evidence of a trend towards privatisation in delivery care between the 1990s and mid-2000s, but with strong differences between countries, which might reflect the heterogeneous nature of this sector both between and within countries. [42] This DHS study highlighted the need for more context-specific data on the nature of the private sector in low and middle-income countries, and its role in maternity care. Further research might focus on understanding the motivations and experiences of women undergoing caesarean sections in private facilities in South Asian settings, pathways for switching between public and private sectors in the event of obstetric complications, a more comprehensive tally of the financial incentives (official or non-official) that motivate private providers to carry out caesareans in each setting, and the consequences of increased caesarean sections in the private sector for maternal and neonatal health outcomes.

In our study, locations with higher prevalence of caesarean deliveries also showed a positive educational gradient even after adjusting for wealth measured by household assets. Moreover, there was an interactive association between education and type of facility, with highly educated women particularly likely to receive caesarean deliveries in private facilities in Bangladesh and informal settlements in urban India. Literature exploring the determinants of caesarean delivery emphasises that multiple influences drive a woman's decision to deliver by caesarean. Profit considerations at the facility level may prompt some providers to urge women to receive unnecessary procedures, while women themselves may prefer caesareans for cultural reasons, fear of painful deliveries, or because they believe it to be safer. [36 43-45] Within the client-provider interaction, providers may be more likely to acquiesce to a request for caesarean from a highly educated woman; and such women may be more likely to accept advice from a provider. [19 46] Our findings lend support to the hypothesis that, while provider-level factors are probably partially responsible for the rapid increase in caesarean deliveries, it is also necessary to consider women's own preferences and decision-making processes and how they are shaped by social and cultural factors.

Strengths and limitations

The strengths of the study were its large sample size and focus on underserved communities, which are a priority for public health interventions in South Asia. It had five main limitations. It was not a nationally or sub-nationally representative study, and districts or clusters were sampled purposively from previous

For peer review only - http://bmjopen.bmj.com/site/about/guidelines.xhtml

cRCTs. This limits the generalisability of our findings to geographical settings outside the study areas. The study was cross-sectional, and therefore only able to suggest associations rather than causal relationships. The data also did not include key predictors of caesarean section such as breech presentation, and whether the previous delivery was by caesarean, which limits the completeness of our analysis of determinants. In addition, levels of serious complications in pregnancy and delivery varied considerably between locations and were often higher than expected, suggesting potential over-reporting and limited reliability as an indicator of complications. Finally, some variables had small denominators, and others had high levels of missing data (for example, maternal age in rural India).

Conclusions

Our study found that delivering in a private health facility was associated with an increased odds of caesarean section in three of four South Asian locations, and that the associations persisted after adjustments for maternal, pregnancy and delivery characteristics, and year of delivery. We also found significant interactive associations between maternal education and caesarean delivery in Bangladesh (both private and public facilities) and informal settlements in urban India (private facilities only). These results lend support to the hypothesis that increased caesarean section rates in these three South Asian countries may in part be driven by the private sector, but also suggest that, in some settings such as Bangladesh and informal settlements in urban India, demand from more educated mothers may play a part. These findings urgently call for greater monitoring of the role of private providers in maternity care, together with action to mitigate the negative consequences of increased caesarean sections in the private sector for maternal and neonatal health outcomes. It remains the explicit responsibility of the medical profession and associated regulatory bodies to ensure that surgical intervention in delivery care is undertaken exclusively in the interests of the mother and unborn child, and not those of care providers. Encouragingly, data recently reviewed in The Lancet's Midwifery Series suggest that, even in countries with a rapid growth in caesarean sections such as Brazil and China, steps can be taken to mitigate over-reliance on obstetric care through the enhancement of midwifery-led services, and that these initiatives could help to curb caesarean section rates. [47] Exploring the revival of midwifery-led services in South Asia is an important future direction for research and action. [48]

For peer review only - http://bmjopen.bmj.com/site/about/guidelines.xhtml

Competing interests

None to declare

Contributors

MN and AP had the original idea for the study. MN designed the data analysis strategy in discussion with AP. MN, GA, AS, CS and NS prepared the data. MN performed the analysis and prepared the tables. NN, PT, NSM, DO, KA, DM and AC are responsible for the trials. AP wrote the first draft of the paper and all authors read and commented on the draft. All authors read and approved the final manuscript.

Funding

MN and NS are funded through a Wellcome Trust Strategic Award (085417MA/Z/08/Z). In Bangladesh, the Perinatal Care Project trial (covering KA and AK) was registered with trial number ISRCTN54792066 and has received funding from the Big Lottery Fund and the Wellcome Trust. In Eastern India, the Ekjut trial (covering NN and PT) was registered with trial number ISRCTN21817853 and funded by the Health Foundation. The Dhanusha (Nepal) trial (covering DSM, NS, BPS, and CS) was registered with trial number ISRCTN87820538 and has received funding from: the UBS Optimus Foundation; USAID; DfID and the Wellcome Trust. The City Initiative for Newborn Health trial led by SNEHA (Mumbai, India) was registered with trial number ISRCTN96256793 and has received funding from the ICICI Foundation for Inclusive Growth and the Wellcome Trust. GA, NSM, and DO are currently funded by The Wellcome Trust (091561/Z/10/Z). The funders played no part in the development of the article or the decision to submit it for publication.

Provenance and peer review

Not commissioned; externally peer reviewed.

For peer review only

REFERENCES:

1. World Health Organisation and UNICEF. Countdown 2015: Building a future for women and Children, the 2012 Report. http://countdown2015mnch.org/documents/2012Report/2012-Complete.pdf [Accessed 9th November 2013]
2. Campbell OM, Graham WJ. Strategies for reducing maternal mortality: getting on with what works. *The Lancet* 2006; 368: 1284-1299.
3. Ronsmans C, De Brouwere V, Dubourg D, *et al*.. Measuring the need for life-saving obstetric surgery in developing countries. *BJOG* 2004; 111: 1027-30.
4. World Health Organization. Appropriate technology for birth. *The Lancet* 1985; 2: 436-7.
5. Betrán AP, Merialdi M, Lauer JA, *et al*. Rates of caesarean section: analysis of global, regional and national estimates. *Paediatric and Perinatal Epidemiology* 2007; 21: 98-113.
6. Cavallaro FL, Cresswell JA, França GVA, *et al*. Trends in Caesarean delivery by country and wealth quintile: cross-sectional surveys in southern Asia and sub-Saharan Africa. *WHO Bulletin* 2013; 91: 897-972.
7. Souza JP, Gülmezoglu A, Lumbiganon P, *et al*. WHO Global Survey on Maternal and Perinatal Health Research Group. Caesarean section without medical indications is associated with an increased risk of adverse short-term maternal outcomes: the 2004–2008 WHO Global Survey on Maternal and Perinatal Health. *BMC Medicine* 2010; 8:71.
8. Kyu HH, Shannon HS, Georgiades K, Boyle MH. Caesarian delivery and neonatal mortality rates in 46 low- and middle-income countries: a propensity score matching and meta-analysis of demographic health survey data. *International Journal of Epidemiology* 2013; 1-11.
9. Gibbons L, Belizán JM, Lauer JA, *et al*. The global numbers and costs of additionally needed and unnecessary caesarean sections performed per year: overuse as a barrier to universal coverage. World Health Report (2010), Background paper. Geneva: World Health Organization.
10. Mitra SN, Nawab Ali M, Islam S, *et al*. Bangladesh Demographic and Health Survey, 1993-1994. Calverton, Maryland: National Institute of Population Research and Training (NIPORT), Mitra and Associates, and Macro International Inc, 1994.
11. National Institute of Population Research and Training (NIPORT), Mitra and Associates, and ICF International. Bangladesh Demographic and Health Survey 2011. Dhaka, Bangladesh and Calverton, Maryland, USA: NIPORT, Mitra and Associates, and ICF International, 2013.
12. International Institute for Population Sciences (IIPS) and Macro International. National Family Health Survey (NFHS 1), 1992–93. India, Mumbai: IIPS, 1995.
13. International Institute for Population Sciences (IIPS), Macro International. National Family Health Survey (NFHS 3), 2005–06; India: Volume I. Mumbai: IIPS, 2007.
14. Pradhan A, Aryal RH, Regmi G, *et al*. Nepal Family Health Survey 1996. Kathmandu, Nepal and

For peer review only - http://bmjopen.bmj.com/site/about/guidelines.xhtml

Calverton, Maryland: Ministry of Health [Nepal], New ERA, and Macro International Inc, 1997.

15. Ministry of Health and Population (MOHP) [Nepal], New ERA, and ICF International Inc. Nepal Demographic and Health Survey 2011. Kathmandu, Nepal: Ministry of Health and Population, New ERA, and ICF International, Calverton, Maryland, 2012.
16. Ajeet S, Nandkishore K. The boom in unnecessary caesarean surgeries is jeopardizing women's health. *Health Care Women International* 2013; 34: 513-521.
17. Sreevidya S, Sathiyasekaran BW. High caesarean rates in Madras (India): a population-based cross-sectional study. *BJOG* 2003; 110: 106-111.
18. Koblinksi M, Anwar I, Mridha MK, *et al*. Reducing maternal mortality and improving maternal health: Bangladesh and MDG 5. *J Health Popul Nutr* 2008; 26: 280-294.
19. Leone T, Padmadas SS, Matthews Z. Community factors influencing rising caesarean section rates in developing countries: an analysis of six countries. *Soc Sci Med* 2008; 67: 1236-46.
20. Mazzoni A, Althabe A, Liu NH, *et al*. Women's preference for caesarean section: a systematic review and meta-analysis of observational studies. *BJOG* 2011; 118: 391-399.
21. Tripathy P, Nair N, Barnett S, et al. Effect of a participatory intervention with women's groups on birth outcomes and maternal depression in Jharkhand and Orissa, India: A cluster-randomised controlled trial. *Lancet* 2010; 375: 1182–1192.
22. Azad K, Barnett S, Banerjee B, *et al*. Effect of scaling up women's groups on birth outcomes in three rural districts in Bangladesh: a cluster-randomised controlled trial. *Lancet* 2010; 375: 1193–1202.
23. Fottrell E, Azad K, Kuddus A, *et al*. The effect of increased coverage of participatory women's groups on neonatal mortality in Bangladesh: a cluster-randomised trial. *JAMA Pediatrics* 2013; 167: 816-825.
24. Shah More N, Bapat U, Das S, *et al*. Cluster-randomised controlled trial of community mobilisation in Mumbai slums to improve care during pregnancy, delivery, postpartum and for the newborn. *Trials* 2008; 9:7.
25. Shrestha BP, Bhandari B, Manandhar DS, *et al*. Community interventions to reduce child mortality in Dhanusha, Nepal: study protocol for a cluster randomized controlled trial. *Trials* 2011; 12: 136.
26. Prost A, Colbourn T, Seward N, *et al*. Women's groups practising participatory learning and action to improve maternal and newborn health in low-resource settings: a systematic review and meta-analysis. *Lancet* 2013; 381: 1736–46.
27. Schmidt J-0, Ensor T, Hossain A, Khan S. Vouchers as demand side financing instruments for health care: A review of the Bangladesh maternal voucher scheme. *Health Policy* 2010; 96: 98–107.
28. Ahmed S, Khan MM. A maternal health voucher scheme: what have we learned from the

For peer review only - http://bmjopen.bmj.com/site/about/guidelines.xhtml

demand-side financing scheme in Bangladesh? *Health Policy & Planning* 2011; 6:25–32.

29. Lim SS, Dandona L, Hoisington JA, *et al*. India's Janani Suraksha Yojana, a conditional cash transfer programme to increase births in health facilities: an impact evaluation**.** *Lancet* 2010; 375: 209-23.
30. Shah More N, Alcock G, Bapat U, *et al*. Tracing pathways from antenatal to delivery care for women in Mumbai, India: cross-sectional study of maternity in low-income areas. International Health 2009; 1: 71–77.
31. Government of India, Ministry of Health & Family Welfare. Janani Suraksha Yojana: Guidelines for Implementation, 2006. http://www.mohfw.nic.in/NRHM/./guidelines/JSY_guidelines_09_06.pdf
32. Witter S, Khadka S, Nath H, Tiwari S. The national free delivery policy in Nepal: early evidence of its effects on health facilities. *Health Policy and Planning* 2011; 26: ii84–ii91.
33. Holgado-Tello FP,Chacón–Moscoso S, Barbero-Garcia I, Vila-Abad E. Polychoric versus Pearson correlations in exploratory and confirmatory factor analysis of ordinal variables. *Quality & Quantity* 2010; 44 (1): 153-166.
34. Rabe-Hesketh S, Skrondal A, Pickles A. Maximum likelihood estimation of limited and discrete dependent variable models with nested random effects. *Journal of Econometrics* 2005; 128: 301-323.
35. StataCorp. 2013. *Stata Statistical Software: Release 13*. College Station, TX: StataCorp LP.
36. Potter J, Berquó E, Perpétuo I, *et al*. Unwanted Caesarean Sections among Public and Private Patients in Brazil: Prospective Study. *BMJ* 2001; 323: 1155-8.
37. Kamal SMM. Preference for institutional delivery and caesarean sections in Bangladesh. *J Health Popul Nutr* 2013; 31: 96-109.
38. Randive B, Diwan V, De Costa A. India's Conditional Cash Transfer Programme (the JSY) to Promote Institutional Birth: Is There an Association between Institutional Birth Proportion and Maternal Mortality? *PLoS ONE* 2013; 8(6): e67452. doi:10.1371/journal.pone.0067452
39. Chatterjee S, Laxminarayan R. Costs of surgical procedures in Indian hospitals. *BMJ Open* 2013; 3: e002844.
40. Parkhurst JO, Rahman SA. Life saving or money wasting? Perceptions of caesarean sections among users of services in Rural Bangladesh. *Health Policy* 2007; 80: 392-401.
41. Khan R, Blum LS, Sultana M, et al. An Examination of Women Experiencing Obstetric Complications Requiring Emergency Care: Perceptions and Sociocultural Consequences of Caesarean Sections in Bangladesh. *J Health Popul Nutr 2012*; 30: 159-171.
42. Pomeroy A, Koblinksy M, Alva S. Private Delivery Care in Developing Countries: Trends and Determinants. DHS Working document 2010; No.76. http://dhsprogram.com/pubs/pdf/WP76/WP76.pdf [Accessed 3rd March 2014].

For peer review only

For peer review only - http://bmjopen.bmj.com/site/about/guidelines.xhtml

43. Sufang G, Padmadas SS, Fengmin Z et al. Delivery settings and caesarean section rates in China. *Bull WHO* 2007; 85: 733-820.

44. Lo JC. Patients' attitudes vs. physicians' determination: implications for cesarean sections. *Soc Sci Med* 2003; 57: 91-6.

45. McCourt C, Weaver J, Statham H, *et al.* Elective cesarean section and decision making: a critical review of the literature. *Birth* 2007; 34: 65-79.

46. Ghetti C, Chan BKS, Guise J-M. Physicians' responses to patient-requested caesarean delivery. *Birth* 2004; 31: 280-4.

47. Renfrew MJ, McFadden A, Bastos MH, *et al*. Midwifery and quality care: findings from a new evidence-informed framework for maternal and newborn care. *Lancet* 2014 http://dx.doi.org/10.1016/ S0140-6736(14)60789-3.

48. Mavalankar D, Sankara Raman P, Vora K. Midwives of India: missing in action. *Midwifery* 2011; 27: 700-6.

For peer review only

Table 1: Characteristics of studies and populations

Study (Country)	Bangladesh (rural)	India (rural)	Nepal (rural)	India (informal urban settlements)
**Location**	Three districts: Bogra, Maulvibazaar and Faridpur	Three districts of Jharkhand and Odisha: Keonjhar, West Singhbhum and Saraikela	Dhanusha district (Terai)	Mumbai slums
**Period**	2005 – 2011	2005-2008	2008-2011	2006-2009
**Estimated population**	532,900	114,000	240,000	283,000
**Cluster characteristics**	Villages making up a union	8-10 villages with residents classified as Scheduled Tribe or Other Backward Class	Village Development Committee	Slum areas in six municipal wards of Mumbai
**Method of cluster identification**	Purposive sampling of three districts and clusters within districts	Purposive sampling of three districts and clusters within districts	Random sampling of 60 clusters from a list of 79 suitable clusters in one district	92 clusters in six municipal wards identified using municipal documents, surveys, discussions with key informants, and site visits. Random selection of 48 clusters for randomized allocation.
**Clusters, n**	9	18	30	24
**Cluster and individual follow-up**	All clusters followed up<br>Interviews completed after 82% of identified births in control areas in Phase 1, and 99% of births in Phase 2	All clusters followed up<br>Interviews completed after 98% of identified births	All clusters followed up	All clusters followed up<br>Interviews completed after 83% of identified births
**Maternal mortality ratio**	254.3	668.1	Unknown	206.2
**Health facilities available in control areas**	Public facilities: District Hospitals; Maternal and Child Welfare Centres; Upazilla Health Complexes. Private facilities: small-to-medium-size clinics; BRAC (NGO) facilities where deliveries do not take place; larger private hospitals with and without Comprehensive emergency obstetric care (CEmOC) facilities.	Public facilities: District Hospitals; Primary Health Centres (PHCs) in which deliveries can notionally take place but that are not usually equipped for CEmOC; Community Health Centres (CHCs) acting as referral centres for PHCs, covering a population of around 80,000 with EmOC facilities; district hospitals. Private and charitable facilities: medium-sized	Public facilities: three Primary Health Care Centres, three Health Posts and 24 Sub-Health Posts, none of which are equipped for CEmOC. These health facilities refer to the public Zonal Tertiary Hospital and various private providers in the district headquarters and nearby medical college, which have facilities for caesarean sections.	Public facilities: Municipal tertiary hospitals, general hospitals, and maternity homes. Private facilities: specialty hospitals, general hospitals, and maternity homes.

For peer review only

missionary hospitals with EmOC facilities.

For peer review only

For peer review only - http://bmjopen.bmj.com/site/about/guidelines.xhtml

Table 2: Characteristics of institutional births and caesarean deliveries by location

	Births	Institutional births						Caesarean section births			
		All		Public facility		Private/charitable facility		Public facility		Private/charitable facility	
	(n)	(n)	(% all births)	(n)	(% institutional births)	(n)	(% institutional births)	(n)	(% deliveries in public facilities)	(n)	(% deliveries in private facilities)
**Bangladesh (rural)**	21,560	4,592	21%	2,053	45%	2,539	55%	589	29%	1,852	73%
**India (rural)**	8,541	1,816	21%	421	23%	1,395	77%	64	15%	66	5%
**India (informal urban settlements)**	10,236	9,259	90%	5,489	59%	3,770	41%	811	15%	697	18%
**Nepal (rural)**	4,931	1,586	32%	1,424	90%	162	10%	201	14%	48	30%

* Nepal numbers weighted using women's probability of selection within each cluster, scaled to total number of institutional births. (Unweighted Nepal totals: 4,990 births and 1,586 institutional births)

For peer review only

Table 3: Mutually adjusted associations of type of facility of delivery and other selected determinants with caesarean section delivery, by location

	**Bangladesh (rural)**		**India (rural)**		**India (informal urban settlements)**		**Nepal (rural)***	
	ORs (95% CIs)		ORs (95% CIs)		ORs (95% CIs)		ORs (95% CIs)	
	Crude	Adjusted	Crude	Adjusted	Crude	Adjusted	Crude	Adjusted
**Health facility characteristics**								
Public health facility (ref)								
Private health facility	6.82 (5.96,7.81)	5.91 (5.15,6.78)	0.39 (0.25,0.61)	0.46 (0.29,0.73)	1.36 (1.21,1.52)	1.22 (1.09,1.38)	2.42 (1.48,3.94)	2.37 (1.62,3.44)
**Pregnancy and delivery characteristics**								
4+ antenatal care visits (fewer or none = ref)**		1.46 (1.26,1.69)		1.49 (0.96,2.32)		1.06 (0.93,1.21)		1.92 (1.43,2.58)
Birth order								
1 (ref)								
2		0.95 (0.80,1.13)		1.25 (0.80,1.95)		0.65 (0.56,0.75)		1.41 (1.02,1.94)
3		1.19 (0.94,1.52)		0.36 (0.15,0.87)		0.60 (0.50,0.71)		0.58 (0.37,0.92)
4+		1.17 (0.90,1.53)		0.36 (0.15,0.85)		0.39 (0.32,0.48)		1.00 (0.54,1.84)
Serious complications in pregnancy/delivery*** (no complications=ref)		0.87 (0.76,1.00)		1.77 (1.17,2.67)		1.71 (1.39,2.11)		4.87 (2.51,9.47)
Multiple birth (ref=single)		0.93 (0.65,1.32)		1.57 (0.52,4.75)		3.01 (2.14,4.23)		3.42 (1.77,6.61)
**Maternal characteristics**								
Age (years)								
15-24 (ref)								
25-34		1.12 (0.94,1.34)		1.81 (1.14,2.86)		1.45 (1.27,1.66)		1.52 (0.63,1.94)
35+		1.10		2.11		1.79		1.55

For peer review only - http://bmjopen.bmj.com/site/about/guidelines.xhtml

For peer review only

	Bangladesh (rural)		India (rural)		India (informal urban settlements)		Nepal (rural)*	
	ORs (95% CIs)		ORs (95% CIs)		ORs (95% CIs)		ORs (95% CIs)	
	Crude	Adjusted	Crude	Adjusted	Crude	Adjusted	Crude	Adjusted
		(0.77,1.58)		(0.63,7.07)		(1.27,2.53)		(0.55,1.47)
Maternal education****								
No formal education (ref)								
Primary education		1.07		0.98		1.13		0.90
		(0.83,1.38)		(0.39,2.47)		(0.85,1.49)		(0.59,1.39)
Secondary education		1.44		1.11		1.22		1.37
		(1.13,1.84)		(0.68,1.80)		(1.04,1.42)		(0.97,1.94)
Bachelor's degree or higher		2.44		1.20		1.62		-
		(1.52,3.92)		(0.43,3.31)		(1.30,2.02)		-
Household wealth quintile								
1st (poorest, ref)								
2nd		1.31		1.61		1.14		1.11
		(1.06,1.63)		(0.69,3.75)		(0.77,1.68)		(0.63,1.94)
3rd		1.41		1.36		1.13		0.90
		(1.10,1.82)		(0.58,3.21)		(0.95,1.33)		(0.55,1.47)
4th (wealthiest)		1.36		2.16		1.50		0.99
		(1.09,1.70)		(0.87,5.33)		(1.27,1.78)		(0.52,1.90)
Year of delivery								
2004-2006 (ref: BD, IN)								-
2007-2009 (ref: NP rural)		0.90		0.90		1.14		-
		(0.74,1.10)		(0.61,1.33)		(1.09,1.38)		-
2010-2012		1.15		-		-		1.29
		(0.94,1.41)		-		-		(0.91,1.82)
N	4,592	4,592	1,816	1,816	9,259	9,259	1,586	1,586

* Nepal numbers weighted using women's probability of selection within each cluster, scaled to total number of institutional births.

** At least one visit with skilled provider

***Includes: symptoms of eclampsia (fits, seizures, convulsions, or unconsciousness during pregnancy or delivery); reduced or no fetal movement; labour lasting more than 24 hours.

****For the Nepal data, two respondents with bachelor's degrees (2 respondent total, 1 delivering in institution) were combined with respondents with secondary education.

For peer review only

All analyses additionally adjusted for survey design using fixed effect of stratum and random effect of cluster.

For peer review only

For peer review only - http://bmjopen.bmj.com/site/about/guidelines.xhtml

Table 4: Interactive associations between type of delivery facility, maternal education, and caesarean delivery, by location

	Bangladesh (rural)		India (rural)		India (informal urban settlements)		Nepal (rural)*	
	(n)	AOR, 95% CI	(n)	AOR, 95% CI	(n)	AOR, 95% CI	(unweighted n)	AOR, 95% CI
Public facility - no maternal education (ref)	202	-	109	-	1,396	-	822	-
Public facility - primary education	448	1.07 (0.76,1.50)	17	0.92 (0.18,4.77)	357	0.88 (0.61,1.27)	196	1.04 (0.65,1.65)
Public facility - secondary education	202	1.63 (1.18,2.24)	604	1.09 (0.52,2.26)	920	1.1 (0.91,1.33)	413	1.45 (1.04,2.02)
Public facility - bachelor's degree	448	4.58 (2.24,9.38)	100	1.55 (0.41,5.89)	180	1.06 (0.76,1.47)	-	- -
Private facility - no education	332	7.24 (4.82,10.87)	109	0.46 (0.21,1.01)	1396	0.91 (0.70,1.19)	100	2.83 (1.73,4.61)
Private facility - primary education	578	7.58 (5.36,10.71)	17	0.47 (0.14,1.58)	357	1.52 (0.99,2.33)	16	0.86 (0.14,5.21)
Private facility - secondary education	1,102	9.03 (6.57,12.41)	274	0.52 (0.24,1.12)	3,382	1.3 (1.06,1.60)	39	3.01 (1.71,5.30)
Private facility - bachelor's degree	41	11.24 (6.37,19.85)	21	0.4 (0.08,2.00)	354	2.1 (1.61,2.75)	-	- -
N	3,353		1,251		8,342		1586	
p-value**	0.021		0.792		0.001		0.394	

All results adjusted for number of antenatal care visits, parity, medical indication for caesarean, multiple birth, maternal age (10-yr group), household assets, stratum, and cluster (random effect).
* Nepal numbers weighted using women's probability of selection within each cluster, scaled to total number of institutional births.

** p-value from -2 log-likelihood test comparing nested models with and without interaction terms

For peer review only - http://bmjopen.bmj.com/site/about/guidelines.xhtml

STROBE 2007 (v4) checklist of items to be included in reports of observational studies in epidemiology*

Checklist for cohort, case-control, and cross-sectional studies (combined)

Section/Topic	Item #	Recommendation	Reported on page #
**Title and abstract**	1	(*a*) Indicate the study's design with a commonly used term in the title or the abstract	1
		(*b*) Provide in the abstract an informative and balanced summary of what was done and what was found	2
**Introduction**			
Background/rationale	2	Explain the scientific background and rationale for the investigation being reported	4
Objectives	3	State specific objectives, including any pre-specified hypotheses	4
**Methods**			
Study design	4	Present key elements of study design early in the paper	1, 5-6
Setting	5	Describe the setting, locations, and relevant dates, including periods of recruitment, exposure, follow-up, and data collection	5 and Table 1
Participants	6	(*a*) *Cohort study*—Give the eligibility criteria, and the sources and methods of selection of participants. Describe methods of follow-up<br>*Case-control study*—Give the eligibility criteria, and the sources and methods of case ascertainment and control selection. Give the rationale for the choice of cases and controls<br>*Cross-sectional study*—Give the eligibility criteria, and the sources and methods of selection of participants	6
		(*b*) *Cohort study*—For matched studies, give matching criteria and number of exposed and unexposed<br>*Case-control study*—For matched studies, give matching criteria and the number of controls per case	
Variables	7	Clearly define all outcomes, exposures, predictors, potential confounders, and effect modifiers. Give diagnostic criteria, if applicable	7
Data sources/ measurement	8*	For each variable of interest, give sources of data and details of methods of assessment (measurement). Describe comparability of assessment methods if there is more than one group	7
Bias	9	Describe any efforts to address potential sources of bias	7-8
Study size	10	Explain how the study size was arrived at	6
Quantitative variables	11	Explain how quantitative variables were handled in the analyses. If applicable, describe which groupings were chosen and why	7
Statistical methods	12	(*a*) Describe all statistical methods, including those used to control for confounding	7-8
		(*b*) Describe any methods used to examine subgroups and interactions	Table 4
		(*c*) Explain how missing data were addressed	7
		(*d*) *Cohort study*—If applicable, explain how loss to follow-up was addressed<br>*Case-control study*—If applicable, explain how matching of cases and controls was addressed	

For peer review only

		Cross-sectional study—If applicable, describe analytical methods taking account of sampling strategy	
		(*e*) Describe any sensitivity analyses	N/A
**Results**			
Participants	13*	(a) Report numbers of individuals at each stage of study—eg numbers potentially eligible, examined for eligibility, confirmed eligible, included in the study, completing follow-up, and analysed	7, 8
		(b) Give reasons for non-participation at each stage	7, 8
		(c) Consider use of a flow diagram	Not used
Descriptive data	14*	(a) Give characteristics of study participants (eg demographic, clinical, social) and information on exposures and potential confounders	Supplemental Table 1
		(b) Indicate number of participants with missing data for each variable of interest	
		(c) *Cohort study*—Summarise follow-up time (eg, average and total amount)	
Outcome data	15*	*Cohort study*—Report numbers of outcome events or summary measures over time	
		Case-control study—Report numbers in each exposure category, or summary measures of exposure	
		Cross-sectional study—Report numbers of outcome events or summary measures	Table 3
Main results	16	(*a*) Give unadjusted estimates and, if applicable, confounder-adjusted estimates and their precision (eg, 95% confidence interval). Make clear which confounders were adjusted for and why they were included	Table 3
		(*b*) Report category boundaries when continuous variables were categorized	
		(*c*) If relevant, consider translating estimates of relative risk into absolute risk for a meaningful time period	
Other analyses	17	Report other analyses done—eg analyses of subgroups and interactions, and sensitivity analyses	Table 4
**Discussion**			
Key results	18	Summarise key results with reference to study objectives	10
Limitations	19	Discuss limitations of the study, taking into account sources of potential bias or imprecision. Discuss both direction and magnitude of any potential bias	12
Interpretation	20	Give a cautious overall interpretation of results considering objectives, limitations, multiplicity of analyses, results from similar studies, and other relevant evidence	10-1
Generalisability	21	Discuss the generalisability (external validity) of the study results	12
**Other information**			
Funding	22	Give the source of funding and the role of the funders for the present study and, if applicable, for the original study on which the present article is based	13

*Give information separately for cases and controls in case-control studies and, if applicable, for exposed and unexposed groups in cohort and cross-sectional studies.
Note: An Explanation and Elaboration article discusses each checklist item and gives methodological background and published examples of transparent reporting. The STROBE checklist is best used in conjunction with this article (freely available on the Web sites of PLoS Medicine at http://www.plosmedicine.org/, Annals of Internal Medicine at http://www.annals.org/, and Epidemiology at http://www.epidem.com/). Information on the STROBE Initiative is available at www.strobe-statement.org.

For peer review only

"Prevalence and determinants of caesarean section in private and public health facilities in underserved South Asian communities: cross-sectional analysis of data from Bangladesh, India, and Nepal"

Supplemental table

Supplemental table 1. Characteristics of institutional deliveries by characteristics of the mother, the delivery facility, and location

	Bangladesh (rural)				India (rural)				India (urban)				Nepal (rural)			
	Not caesarean	Caesarean	Total		Not caesarean	Caesarean	Total		Not caesarean	Caesarean	Total		Not caesarean	Caesarean	Total	
	(%)	(%)	(n)	(%)	(%)	(%)	(n)	(%)	(%)	(%)	(n)	(%)	(%)	(%)	(n)	(%)
**Health facility characteristics**																
Public health facility	68.1	24.1	2053	44.7	21.2	49.2	421	23.2	60.4	53.8	5489	59.3	91.5	80.7	1424	89.8
Private/charitable health facility	31.9	75.9	2539	55.3	78.8	50.8	1395	76.9	39.6	46.2	3770	40.7	8.5	19.3	162	10.2
**Pregnancy and delivery characteristics**																
4+ antenatal care visits*	25.2	38.8	1491	67.5	47.1	56.2	867	52.3	73.4	74.2	6810	26.4	41.3	53.6	686	43.2
Birth order																
1	47.1	47.4	2170	47.3	51.6	55.4	942	51.9	29.7	41.6	2932	31.7	50.1	46.7	786	49.6
2	25.9	26.1	1194	26	23	32.3	430	23.7	27.9	26.1	2554	27.6	22.5	27.1	368	23.2
3	12.4	13.8	603	13.1	11.7	5.4	204	11.2	19.4	17.6	1769	19.1	14.9	10.3	225	14.2
4+	14.6	12.7	625	13.6	13.7	6.9	240	13.2	23	14.7	2004	21.6	12.5	15.9	207	13
Serious complications in pregnancy/delivery**	53.4	43.5	2212	48.2	29.2	44.6	550	30.3	6.1	9.2	608	6.6	21.2	38.5	379	23.9
Multiple birth	3.6	3.9	173	3.8	2.1	3.1	40	2.2	1.4	3.6	163	1.8	2.2	7.8	48	3.1
**Maternal socio-demographic characteristics**																
Maternal age (years)																
15-24	59.8	55.8	2650	57.7	63.6	60	1150	63.3	53.1	51.5	4894	52.9	71.6	66.3	1123	70.8
25-34	34.4	39.5	1705	37.1	32.7	36.9	600	33	43.6	45.3	4060	43.8	26.4	31	431	27.2
35+	5.8	4.6	237	5.2	3.7	3.1	66	3.6	3.3	3.2	305	3.3	1.9	2.7	33	2.1
Maternal education																
No formal education	15.2	8.3	531	11.6	40.1	28.5	713	39.3	26.3	18.5	2316	25	58.4	57.2	924	58.2
Primary education	27.3	17.9	1025	22.3	6.6	4.6	117	6.4	6	4.9	537	5.8	13.6	10.8	209	13.2
Secondary education	55.7	68.6	2872	62.5	50.2	62.3	927	51	59.6	61.9	5550	59.9	28	32	453	28.6
Bachelor's degree or higher***	1.7	5.2	164	3.6	3.1	4.6	59	3.2	8.2	14.7	856	9.2	-	-	-	-

For peer review only

Household wealth quintile																
1st (poorest)	23	10.4	749	16.3	12.6	5.4	220	12.1	24.1	17.2	2125	23	14.2	13.4	223	14.1
2nd	32.4	31.5	1465	31.9	30	31.5	547	30.1	3	2.3	268	2.9	22	22.4	350	22.1
3rd	14	15.2	673	14.7	39.9	32.3	715	39.4	35.9	31.3	3254	35.1	33.9	30.5	529	33.4
4th (wealthiest)	30.5	42.9	1705	37.1	17.4	30.8	334	18.4	37	49.3	3612	39	29.9	33.6	483	30.5
**Year of delivery**																
2004-2006	19.2	15.2	782	17	36.7	40.8	672	37	46.4	42.2	4237	45.8	-	-	-	-
2007-2009	44.7	38.7	1907	41.5	63.3	59.2	1144	63	53.6	57.8	5022	54.2	45.1	45.7	717	45.2
2010-2012	36.1	46.1	1903	41.4	-	-	-	-	-	-	-	-	54.9	54.3	869	54.8
Total (n)	2151	2441	4592	100	1686	130	4592	100	7751	1508	9259	100	734	135	869	100

* Nepal numbers weighted using women's probability of selection within each cluster, scaled to total number of institutional births.

** At least one visit with skilled provider

***Includes: symptoms of pre-eclampsia (swollen face or limbs or blurred vision [not asked in Nepal]), eclampsia (fits, seizures, convulsions, or unconsciousness during pregnancy or delivery); haemorrhaging during delivery, labour lasting more than 24 hours.

****For the Nepal data, secondary and bachelor's (2 respondent total, 1 delivering in institution) were combined.

For peer review only

Prevalence and determinants of caesarean section in private and public health facilities in underserved South Asian communities: cross-sectional analysis of data from Bangladesh, India, and Nepal

Melissa Neuman[1], Glyn Alcock[1], Kishwar Azad[2], Abdul Kuddus[2], David Osrin[1], Neena Shah More[3], Nirmala Nair[4], Prasanta Tripathy[4], Catherine Sikorski[1], Naomi Saville[1], Aman Sen[5], Tim Colbourn[1], Tanja AJ Houweling[6], Nadine Seward[1], Dharma S Manandhar[5], Bhim P Shrestha[5], Anthony Costello[1] and Audrey Prost[1]

Author affiliations:

[1] Institute for Global Health, University College London, 30 Guilford Street, London WC1N 1EH, UK

[2] Perinatal Care Project, Diabetic Association of Bangladesh, 122 Kazi Nazrul Islam Avenue, Dhaka 1000, Bangladesh

[3] Society for Nutrition, Education and Health Action (SNEHA), Urban Health Centre, Shahunagar, Dharavi, Mumbai 400017, Maharashtra, India

[4] Ekjut, Plot 556B, Potka, Chakradharpur, West Singhbhum, Jharkhand, India

[5] Mother and Infant Research Activities (MIRA), YB Bhavan, Thapathali, GPO Box 921, Kathmandu, Nepal

[6] Department of Public Health, Erasmus MC University Medical Center Rotterdam, P.O. Box 2040, 3000 CA Rotterdam, The Netherlands

Proposed running head: Caesarean sections in private vs. public health facilities in South Asia

Word count: 4393

Figures and tables: 0 figures and 4 tables

For peer review only

For peer review only - http://bmjopen.bmj.com/site/about/guidelines.xhtml

ABSTRACT (Max: 300 words; Current: 298)

Objectives: To describe the prevalence and determinants of births by caesarean section in private and public health facilities in underserved communities in South Asia.

Design: Secondary, cross-sectional analysis.

Setting: 81 community-based geographical clusters in four locations in Bangladesh, India and Nepal (three rural, one urban).

Participants: 45,327 births (2005 to 2012).

Outcome measures: proportion of caesarean section delivery by location and type of facility; determinants of caesarean section delivery by location.

Results: Institutional delivery rates varied widely between settings, from 21% in rural India to 90% in informal ~~urban~~ settlements in urban India. The proportion of births by caesarean section in private facilities versus public facilities was 73% vs. 29% in Bangladesh, 30% vs. 14% in rural Nepal, 18% vs. 15% in informal Indian urban settlements, and 5% vs. 15% in rural India. The odds of caesarean section were greater in private facilities than in public facilities in three of four study locations, even when adjusted for pregnancy and delivery characteristics, maternal characteristics, and year of delivery (Bangladesh AOR: 5.91, 95% CI: 5.15-6.78; Nepal AOR: 2.37, 95% CI: 1.62-3.44; informal settlements in urban India AOR: 1.22, 95% CI: 1.09-1.38). We found significant interactive associations between maternal education and private facility delivery in two of four locations (p=0.025 in rural Bangladesh and p<0.001 in informal settlements in urban India), with highly educated women particularly likely to deliver by caesarean in private facilities.

Conclusions:
Our results lend support to the hypothesis that increased caesarean section rates in these South Asian countries may be driven in part by the private sector. They also suggest that preferences for caesarean delivery may be stronger among highly educated women, and that individual- and provider-level factors interact in driving caesarean rates upwards. Increasing rates of caesarean section in the private sector require close monitoring and action.

For peer review only

Article summary

Article focus

- Caesarean sections are on the rise in South Asia. Research suggests that private health facilities may be contributing to this by conducting caesareans over and above clinical need.
- Literature from other settings indicates that increases in caesarean sections are shaped by both supply and demand pressures: providers often have financial incentives to intervene surgically, and women of higher socio-economic status are also more likely to want caesareans.
- This study examined the prevalence and determinants of caesarean sections in private and charitable facilities compared with public health facilities for over 45,000 births in underserved communities of rural Nepal, rural Bangladesh, rural India, and informal settlements in urban Indiaurban India.

Key messages

- Caesarean sections were more common in private than in public health facilities in three of four study locations (urban India, rural Nepal, and rural Bangladesh, and informal settlements in urban India).
- We found significant interactive associations between maternal education and caesarean delivery in Bangladesh (both private and public facilities) and in informal Indian urban settlements urban India (private facilities only).
- Future research should monitor caesarean section rates in the private sector in South Asia and subsequent maternal and neonatal health outcomes

Strengths and limitations

- This study had a large sample size and focused on underserved communities, which are a priority for public health interventions in South Asia.
- Our data were not nationally or sub-nationally -representative, which limits the generalisability of our findings. Other limitations included small denominators and missing data for some variables. We did not have data on some known predictors of caesarean section, which would have enhanced the completeness of our determinants analysis.

For peer review only

INTRODUCTION

Access to comprehensive emergency obstetric care, including caesarean section, is key to preventing the estimated 287,000 maternal and 2.9 million neonatal deaths that occur worldwide every year.[1 2] Although debate continues about how to quantify the need for life-saving obstetric surgery, a 1985 World Health Organization (WHO) report suggested that the optimal population range for caesarean section rates is between 5% and 15%, and this endures as a reference.[3 4] Caesarean section rates are increasing worldwide, albeit unequally: a recent analysis of Demographic and Health Survey (DHS) data in 26 South Asian and sub-Saharan African countries found that rates were highest among the 'urban rich' in all countries, and lowest among the 'rural poor' in 18.[5] In all countries, fewer than 5% of mothers in the poorest wealth quintile delivered by caesarean.[6]

Caesarean sections conducted without clinical need can have adverse consequences for mothers and children. A 2008 WHO survey of 373 facilities across 24 countries found that unnecessary caesareans were associated with an increased risk of maternal mortality and serious outcomes for mothers and newborn infants, compared with spontaneous vaginal delivery.[7] Recent ecological analyses also highlighted strong associations between caesarean delivery and increased neonatal mortality in countries with low and medium caesarean section rates.[8] Unnecessary caesareans lead to considerable costs for families and health systems: an estimated 6.2 million unnecessary procedures were performed in 2008, costing approximately US$ 2.32 billion. [9]

Several South Asian countries have recorded substantial increases in caesarean section rates over the past decade. In Bangladesh, rates rose from 2% (2000) to 17% (2011); in India, from 3% (1992) to 11 % (2006); and in Nepal, from 1% (2000) to 5% (2011).[10-5] Several studies in these countries have raised concerns about high caesarean rates in private facilities, and a recent DHS analysis speculated that national increases in caesarean section rates in South Asian countries could be driven in part by higher rates among deliveries in private sector facilities[.6 16-18] Literature from other settings indicates that increases in caesarean sections are shaped by both supply and demand pressures: providers often have financial incentives to intervene surgically, and women of higher socio-economic status are also more likely to desire caesareans.[19 20]

There are few large, recent community-based studies from South Asia quantifying differences in caesarean section rates between public and private facilities. Such studies are necessary in order to examine whether increases in caesareans in these settings are indeed likely to be driven by the private sector, demand from wealthier and more educated mothers, or a combination of the two. We conducted a cross-sectional analysis of data from Bangladesh, India, and Nepal to explore the prevalence and determinants of caesarean section delivery by type of facility and location, and to examine the

For peer review only

interactions between maternal education and caesarean section delivery in private and public facilities.

METHODS

Study populations

We used data collected through vital events surveillance systems established during four cluster-randomised controlled trials conducted between 2005 and 2011. The trials were done with communities that can be considered socio-economically disadvantaged: in Bangladesh and Nepal, they took place in four rural, underserved districts (Bogra, Maulvibazaar, and Faridpur in Bangladesh, and the Terai district of Dhanusha, Nepal); in rural India, most participants were from Scheduled Tribes in two states of eastern India (Jharkhand and Odisha); in urban India, data came from informal settlements (slums) slum communities in Mumbai. **Table 1** describes the characteristics of each study and its population, including the background maternal mortality ratio and types of facilities present in the study areas. The original trials were designed to evaluate the impact of participatory women's groups on maternal and neonatal health outcomes.[21-25] We used data from the control areas of these trials only, because the women's group intervention led to changes in mortality and practices in several locations.[26]

Health system contexts

All three study countries have experienced substantial increases in institutional delivery rates over the last two decades: births in health facilities increased from 4% (1993) to 29% (2011) in Bangladesh; from 26% (1992-3) to 47% (2007-8) in India; and from 8% (1996) to 35% (2011) in Nepal.[10-15] All three countries have implemented incentive schemes to promote institutional delivery, though these have varying coverage. In 2010, Bangladesh's pilot maternity voucher scheme reached an estimated 10.4 million people across 31 sub-districts, around 7% of the country's population.[27 28] Mothers receive a cash incentive for antenatal care and delivery in a public or private facility, or at home with a skilled birth attendant. Both government and private facility staff also receive cash incentives, including 3000 Bangladeshi Taka (US $38.5) for a caesarean section and 300 Taka for a normal delivery.[28] This maternity incentive scheme was operational in two of the three districts (Faridpur and Maulvibazaar) covered by our study. All three districts had public facilities including District Hospitals, Maternal and Child Welfare Centres, and Upazilla Health Complexes. Private facilities included a number of small-to-medium-size clinics, BRAC (NGO) facilities, and larger private hospitals.

In India, the *Janani Suraksha Yojana* (JSY) maternity incentive scheme entitles women in rural areas of high focus states, including those in this study, to 1400 Indian Rupees ($22.4) after delivering in a government or accredited private health facility. Local community health volunteers called Accredited Social Health Activists (ASHAs) also receive 600 Indian Rupees ($9.6) for identifying pregnant women and helping them get to a health facility.[29] Although the rural Indian data included in this study were

For peer review only - http://bmjopen.bmj.com/site/about/guidelines.xhtml

collected between 2005 and 2008, the JSY was only operational in the study areas from 2008 onwards and its impact is unlikely to be reflected here. The Indian urban area included in our study spanned informal settlements (slums) with a wealth of public and private providers.[30] Mothers with Below Poverty Line cards in such areas are eligible for JSY and can receive Rs 500 towards the costs of delivering in a health facility.[31] Nepal began a safe delivery incentive scheme in 2005 and free deliveries have been available in government facilities since 2009 through the *Aama Surakshya* programme.[32] Dhanusha district, from which data for this study come, has one zonal tertiary hospital and a private medical college hospital equipped for caesarean -sections, and a variety of small public and private health facilities without comprehensive obstetric care.

Data collection

All data were collected using surveillance systems to monitor births and deaths prospectively.[21-25] In all study locations, a female community-based key informant reported births and deaths in her area, which covered a population ranging from 250 to 350. A trained interviewer then verified these reports and paid the informant an incentive for each correct identification. In Bangladesh, rural and urban India, the interviewer administered a structured questionnaire to all eligible mothers around six weeks after delivery; in Nepal, all births in the study area were registered, and interviews were conducted on all births in small clusters and on a random sample of 10 births per month in the larger clusters. In each study location, mothers were interviewed using a questionnaire to collect information about events in the antenatal, delivery and postnatal periods. In each trial and in this subsequent study, participants were women of reproductive age (15-49) who delivered in the study area during the data collection period, and who consented to be interviewed six weeks after delivery, and their infants.

Study sample

The initial sample for this analysis included 46,393 births in Mumbai, rural India, rural Bangladesh, or rural Nepal, of whom 17,565 (38%) were delivered in health care facilities. We excluded the 2348 births occurring outside Mumbai, because we collected limited information on delivery location for these. Additionally, 223 deliveries had missing data on form of delivery (caesarean or vaginal), 602 had missing or implausible values for mother's age, and 98 were missing information on other maternal characteristics (educational attainment, household assets, number of prior pregnancies, number of antenatal visits, severe problems in pregnancy or delivery). 143 births had missing or incomplete information on place of delivery. The final analytic sample included 45,327 births (97.7% of the initial sample).

For peer review only - http://bmjopen.bmj.com/site/about/guidelines.xhtml

Measures used

The primary outcome in these analyses was caesarean section delivery, identified by self-report from the mother or another household member around six weeks after giving birth. The main covariate of interest was the type of delivery facility, coded as public (e.g. district hospitals), private (individual private clinics or hospitals), or NGO/charitable (e.g. Christian missionary hospitals in rural India; BRAC clinics in Bangladesh). We grouped private and NGO/charitable facilities together due to the small number of births in NGO/charitable facilities (6% of institutional deliveries in Bangladesh, 7% in rural India, and 0 in informal settlements in urban India and rural Nepal; data on request). While we refer to all results from Mumbai as being from 'informal settlements', is worth noting that the health facilities used by women in informal settlements for delivery are not always in the settlements themselves: women often travel to other parts of the city to seek care with a recommended or preferred provider, usually private. Additional covariates in the models included measures of the characteristics of the pregnancy and delivery, maternal socio-demographic characteristics, and the location in which the delivery occurred. We created an indicator variable to identify women experiencing serious problems during pregnancy or delivery, with women reporting symptoms of pre-eclampsia (blurred vision or swelling of face and hands), symptoms of eclampsia (fits or seizures during pregnancy or delivery), hæmorrhage during delivery, or labour lasting more than 24 hours considered to have serious complications. Other characteristics of the pregnancy and delivery considered in the analysis included full utilisation of antenatal care, number of prior pregnancies, and multiple pregnancy. Full utilization of antenatal care was defined as four or more visits to antenatal care, with at least one visit to a skilled provider. Number of pregnancies was entered as an ordered categorical variable, with categories of one (first), two, three, or four or more pregnancies.

Maternal socio-demographic characteristics included in the models were: mother's age at delivery, her educational attainment, and household assets. Mother's age was entered into the model as a categorical measure in 10-year groups. Educational attainment was entered as a categorical variable using the following categories: no formal education, primary education, secondary education, or bachelor's degree or higher. To develop an asset index, we used polychoric factor analysis on data on common assets and amenities found in the mother's household, and grouped the resulting factor scores into quartiles.[33] Assets and amenities included electricity, radio or cassette player, electric fan (Bangladesh and India only), television, refrigerator (Bangladesh and India only), telephone (Bangladesh and Nepal only), generator (India only), and bicycle. All models were additionally adjusted for location (Bangladesh, India, Nepal), and year of interview in three-year groups. The data were collected in a stratified, cluster-sampled survey, and we accounted for survey design in the analysis using a fixed effect for stratum and a random effect for cluster.

For peer review only - http://bmjopen.bmj.com/site/about/guidelines.xhtml

Statistical analysis

We used frequencies to describe caesarean section rates by delivery location at each site. We used the Generalized Linear Latent And Mixed Models (GLLAMM) procedure in Stata 13.1, with adaptive quadrature for binary outcomes, to estimate the crude association between type of delivery facility and caesarean section.[34 35] We identified other maternal, pregnancy, and delivery characteristics potentially associated with caesarean section using existing literature, especially studies resulting from the WHO multi-country surveys, and entered these in adjusted models to explore how they modified the association between type of delivery facility and caesarean section, and their individual association with caesarean section. Some South Asian and Latin American studies have detected a strong association between maternal education and caesarean delivery. [7 36 37] We fitted models including indicator variables for each group of mothers by education and type of delivery facility to explore differences in the strength of association between caesarean delivery and private facility by mother's education. To account for the sampling procedure used in rural Nepal, models were adjusted using pweights (probability of selection within cluster); these weights were rescaled to reflect the total number of institutional deliveries.

Ethical approval

The trials that provided data for this study received ethical approval from the following committees: the ethics committee of the Diabetic Association of Bangladesh (Perinatal Care Project, Bangladesh Diabetes Somity, BADAS); an independent ethics committee in Jamshedpur, Jharkhand, India; the Nepal Health Research Council; the Municipal Corporation of Greater Mumbai and the Independent Ethics Committee for Research on Human Subjects (Mumbai, India); the ethics committee of the Institute of Child Health, University College London.

RESULTS

Table 2 describes the characteristics of institutional births and caesarean deliveries by location. We analysed data for 45,327 births: 21,560 in rural Bangladesh, 8,541 in rural India, 10,236 in ~~urban India~~informal settlements in urban India, and 4,931 in rural Nepal. The proportion of women delivering in health facilities varied widely between locations, from 90% in urban India to 21% in rural India. There were also large variations in the proportion of women delivering in private/charitable facilities rather than public facilities, with the highest (77%) in rural India and the lowest (10%) in rural Nepal. The proportion of women giving birth by caesarean section in private rather than public facilities also varied widely between settings. In Bangladesh, only 21% of women delivered in a health facility, around half of them in the private/charitable sector, but 73% of private facility births were by caesarean section. In rural Nepal, 30% of the 162 private facility births were by caesarean section. In informal settlements of Mumbai, 15% of public facility deliveries were caesareans, compared with 18% of private facility

For peer review only - http://bmjopen.bmj.com/site/about/guidelines.xhtml

deliveries. In rural India, caesarean sections were more commonly performed in public health facilities than private or charitable facilities (15% vs. 5%). **Supplemental table 1** describes the characteristics of institutional deliveries (caesarean or non-caesarean) according to type of delivery facility (private or public), maternal socio-economic and socio-demographic characteristics, pregnancy and delivery characteristics, and year of delivery.

Table 3 shows crude and adjusted measures of association between type of delivery facility (private or public) and caesarean section for each location. Delivering in a private health facility was associated with an increased odds of caesarean section in all but one location (rural India). The relative odds of a caesarean in a private facility were greatest in Bangladesh (OR: 6.82, 95% CI: 5.96, 7.81), followed by Nepal (OR: 2.42, 95% CI: 1.48, 3.94) and informal settlements in urban India urban India (OR 1.36, 95% CI: 1.21, 1.52). These associations persisted and were only mildly attenuated when adjusted for maternal characteristics, pregnancy and delivery characteristics, and year of delivery (Bangladesh AOR: 5.91, 95% CI: 5.15, 6.78; Nepal AOR: 2.37, 95% CI: 1.62, 3.44; informal settlements in urban India urban India AOR: 1.22, 95% CI: 1.09, 1.38).

Women who had four or more antenatal check-ups were significantly more likely to have a caesarean delivery in rural Bangladesh and Nepal, with positive but non-significant trends in all other locations. Parity was not associated with caesarean delivery in any location. Having a serious health complication during pregnancy and delivery was associated with caesarean delivery in all locations except rural Bangladesh, where we observed a negative association (AOR: 0.87, 95% CI: 0.76, 1.00). Multiple birth was associated with an increased odds of caesarean section only in informal settlements in urban India urban India and in rural Nepal. Higher maternal age was only associated with an increased odds of caesarean section in informal settlements in urban Indiaurban India. Maternal education was only associated with an increased odds of caesarean delivery in rural Bangladesh and informal settlements in urban Indiaurban India, with mothers with secondary education or Bachelor's degrees having higher odds (AOR: 1.44, 95% CI: 1.13, 1.84 and AOR 2.44, 95% CI: 1.52, 3.92 for Bangladesh and AOR: 1.22, 95% CI: 1.04, 1.42 and 1.62, 95% CI: 1.30, 2.02 for urban informal settlements in urban IndiaIndia, respectively). There were significant positive associations only in rural Bangladesh, with small increases in odds of caesarean section for unit increase in wealth quartile (AOR comparing wealthiest to poorest group in Bangladesh: 1.36, 95% CI: 1.09,1.70), and in informal settlements in urban Indiaurban India, for the wealthiest quartile (AOR: 1.50, 95% CI: 1.27, 1.78).

We found interactive associationsinteractions between maternal education and private facility delivery in two of four sites (p=0.021 in rural Bangladesh and p<0.001 in informal settlements in urban Indiaurban India; **table 4**). In Bangladesh, there was a pronounced positive educational gradient in

For peer review only

caesarean delivery within those who delivered in private facilities, as well as a clear positive association between private facility delivery and caesarean delivery. Women with bachelor's degrees delivering in private facilities had 11 times greater odds of delivering by caesarean (AOR 11.09, 95% CI: 6.28, 19.57) than did women with no formal education delivering in public facilities. Women with no formal education delivering in private facilities had nearly seven times the odds of caesarean compared with the reference group (AOR 6.94, 95% CI: 4.64, 10.39). A similar positive gradient was seen among women delivering in public facilities, with women with bachelor's education having four times greater odds of caesarean delivery in public facilities than women with no formal education (AOR 4.55, 95% CI: 2.22, 9.33). There was also an educational gradient among women in informal settlements in urban India delivering in private facilitieswomen delivering in private facilities in urban India. Educated mothers delivering in private facilities were more likely to deliver by caesarean than were mothers in other groups (AOR 2.10; 95% CI: 1.61, 2.75). However, there was no apparent gradient by educational attainment among women delivering in public facilities.

Formatted: Font: 11 pt

DISCUSSION

Our analysis of data from over 45,000 births confirms the findings of other studies identifying differences in caesarean rates between public and private facilities, and suggests that, even in underserved areas in South Asia, caesareans without medical indication are of concern. In three of four locations, rates of caesarean section were higher in private/charitable facilities than in public facilities. The findings from Bangladesh are particularly noteworthy as they show much greater odds of caesarean section in private facilities, concurring with previous analyses.[37] This was also the only location where serious complications in pregnancy and delivery were not associated with caesarean delivery, suggesting that obstetric surgery was performed over and above clinical need.

Our findings confirm the results of earlier studies of the prevalence of caesarean delivery in South Asia, and indicate that high rates can be found in underserved rural areas. In India, a recent analysis of 2010-11 Annual Health Survey (AHS) data from 284 districts in nine States, including Jharkhand, found that the median caesarean section rate in the private sector was 28%, compared with 5% in the public sector.[38] There appear to be strong financial incentives for surgical procedures in the private sector.[39] In a rural, largely indigenous part of eastern India where more mothers delivered in private/charitable facilities than in public facilities, more caesarean sections were performed in the public sector. In this particular setting, our field experience suggests that only women with serious complications would go to a facility and have a caesarean section, especially as JSY was not yet available in the study areas at the time of data collection. Such women are likely to have experienced multiple referrals from either ill-equipped public or private facilities not wanting to take the risk of admission. It is however possible that

For peer review only - http://bmjopen.bmj.com/site/about/guidelines.xhtml

preference for the private sector has changed in Jharkhand since the advent of JSY, and further disaggregated analyses of Annual Health Survey data would allow a more contemporary exploration of State-level variations in caesarean section rates between public and private sectors.

In Mumbai slums with a high uptake of institutional births and a 60/40 split in favour of the public sector for delivery care, we found no difference in caesarean section rates between the two sectors, which is somewhat surprising given the high rates of caesareans observed in the private sector in nine other states of India (excluding Maharashtra).[38] At least two scenarios may be relevant in this setting. First, public sector hospitals are able to provide caesarean section, while smaller private maternity homes may not be able to. Second, the costs of caesarean section borne by private sector providers are higher in Mumbai, and families may be unwilling to bear these additional costs. This provides motivation for private providers not to provide caesareans, and makes it likely that women experiencing complications move into the public sector. The variety of facility options in an urban environment means that mothers are able to choose between different types of facilities, while in rural areas only one provider may be accessible.

Formatted: Superscript

Analyses using the most recent Bangladesh DHS found that, in 2011, three in five facility births were delivered by caesarean section. This reflects a historical trend: in 2001-2003, nearly half of deliveries in private facilities in Bangladesh were already by caesarean section.[18] Our data from rural, socio-economically disadvantaged communities in three districts of Bangladesh confirm these population-level findings, but also suggest that high caesarean section rates in private facilities are not merely an issue for wealthy urban mothers. Although some research from Bangladesh suggests that mothers may have a preference for caesarean delivery because of fear of labour pain or a desire to select an auspicious date for the birth, other studies also highlight women's fears of caesarean section and their distrust of health providers who recommend them, mainly because of the high costs associated with the procedure.[37 40 41] A qualitative study involving twenty women who had experienced obstetric complications in Matlab in 2008-9 found that most of the fourteen women who had undergone caesareans had spent over Tk 14,999 (US$ 217) on the procedure, which was approximately one-third of GDP per capita at the time.[41] The lack of association between caesareans and complications in pregnancy or delivery and multiple births, coupled with the high financial incentives given to providers for performing caesarean sections ~~and the requirement for junior doctors to 'practise' their surgical skills~~, further suggest that obstetric surgery is being used over and above clinical need. A possible explanation for the significant interactive associations between maternal education and caesarean delivery in both public and private facilities in Bangladesh is that well-educated women may be delivering in more expensive or highly rated institutions, which may in turn be more likely to do caesarean sections for financial reasons ~~and if they act as training centres for junior doctors~~. Our own experience also suggests that such facilities have

For peer review only - http://bmjopen.bmj.com/site/about/guidelines.xhtml

additional incentives to encourage caesareans in order to allow junior doctors to practice their surgical skills.

In our sample of institutional deliveries from rural Nepal, 5~~16~~% of all ~~facility~~ births were by caesarean, which is similar to ~~higher than~~ the national average: the 2011 Nepal DHS found an overall national caesarean section rate of 4.6~~5~~%, with sections more commonly performed for births to highly educated mothers (13%) and mothers in the highest wealth quintile (14%). [26] ~~It is possible that women with complications are more likely to deliver in facilities, and also that, as in Bangladesh, private providers are motivated by financial incentives to conduct caesareans more frequently than strictly necessary. Women who can afford to seek care in private facilities may also be more willing or able to pay for caesarean sections, and providers may conduct more of them to increase their income.~~

A 2010 analysis of DHS data examining the role of the private sector in maternity care in 16 countries found evidence of a trend towards privatisation in delivery care between the 1990s and mid-2000s, but with strong differences between countries, which might reflect the heterogeneous nature of this sector both between and within countries. [42] This DHS study highlighted the need for more context-specific data on the nature of the private sector in low and middle-income countries, and its role in maternity care. Further research might focus on understanding the motivations and experiences of women undergoing caesarean sections in private facilities in South Asian settings, pathways for switching between public and private sectors in the event of obstetric complications, a more comprehensive tally of the financial incentives (official or non-official) that motivate private providers to carry out caesareans in each setting, and the consequences of increased caesarean sections in the private sector for maternal and neonatal health outcomes.

In our study, locations with higher prevalence of caesarean deliveries also showed a positive educational gradient even after adjusting for wealth measured by household assets. Moreover, there was an interactive association between education and type of facility, with highly educated women particularly likely to receive caesarean deliveries in private facilities in Bangladesh and informal settlements in urban India~~urban India~~. Literature exploring the determinants of caesarean delivery emphasises that multiple influences drive a woman's decision to deliver by caesarean. Profit considerations at the facility level may prompt some providers to urge women to receive unnecessary procedures, while women themselves may prefer caesareans for cultural reasons, fear of painful deliveries, or because they believe it to be safer. [36 43-45] Within the client-provider interaction, providers may be more likely to acquiesce to a request for caesarean from a highly educated woman; and such women may be more likely to accept advice from a provider. [19 46] Our findings lend support to the hypothesis that, while provider-level factors are probably partially responsible for the rapid increase in caesarean deliveries, it is also necessary to

For peer review only

For peer review only - http://bmjopen.bmj.com/site/about/guidelines.xhtml

consider women's own preferences and decision-making processes and how they are shaped by social and cultural factors.

Strengths and limitations

The strengths of the study were its large sample size and focus on underserved communities, which are a priority for public health interventions in South Asia. It had five main limitations. It was not a nationally or sub-nationally representative study, and districts or clusters were sampled purposively from previous cRCTs. This limits the generalisability of our findings to geographical settings outside the study areas. The study was cross-sectional, and therefore only able to suggest associations rather than causal relationships. The data also did not include key predictors of caesarean section such as breech presentation, and whether the previous delivery was by caesarean, which limits the completeness of our analysis of determinants. In addition, levels of serious complications in pregnancy and delivery varied considerably between locations and were often higher than expected, suggesting potential over-reporting and limited reliability as an indicator of complications. Finally, some variables had small denominators, and others had high levels of missing data (for example, maternal age in rural India).

Conclusions

Our study found that delivering in a private health facility was associated with an increased odds of caesarean section in three of four South Asian locations, and that the associations persisted after adjustments for maternal, pregnancy and delivery characteristics, and year of delivery. We also found significant interactive associations between maternal education and caesarean delivery in Bangladesh (both private and public facilities) and informal settlements in urban India ~~urban India~~ (private facilities only). These results lend support to the hypothesis that increased caesarean section rates in these three South Asian countries may in part be driven by the private sector, but also suggest that, in some settings such as Bangladesh and informal settlements in urban India~~urban India~~, demand from more educated mothers may play a part. These findings urgently call for greater monitoring of the role of private providers in maternity care, together with action to mitigate the negative consequences of increased caesarean sections in the private sector for maternal and neonatal health outcomes. It remains the explicit responsibility of the medical profession and associated regulatory bodies to ensure that surgical intervention in delivery care is undertaken exclusively in the interests of the mother and unborn child, and not those of care providers. Encouragingly, data recently reviewed in The Lancet's Midwifery Series suggest that, even in countries with a rapid growth in caesarean sections such as Brazil and China, steps can be taken to mitigate over-reliance on obstetric care through the enhancement of midwifery-led services, and that these initiatives could help to curb caesarean section rates. [47] Exploring the revival of midwifery-led services in South Asia is an important future direction for research and action. [48]

Formatted: No widow/orphan control, Don't adjust space between Latin and Asian text, Don't adjust space between Asian text and numbers, Tab stops: Not at 1.3"

Formatted: Font: 11 pt

For peer review only - http://bmjopen.bmj.com/site/about/guidelines.xhtml

Competing interests

None to declare

Contributors

MN and AP had the original idea for the study. MN designed the data analysis strategy in discussion with AP. MN, GA, AS, CS and NS prepared the data. MN performed the analysis and prepared the tables. NN, PT, NSM, DO, KA, DM and AC are responsible for the trials. AP wrote the first draft of the paper and all authors read and commented on the draft. All authors read and approved the final manuscript.

Funding

MN and NS are funded through a Wellcome Trust Strategic Award (085417MA/Z/08/Z). In Bangladesh, the Perinatal Care Project trial (covering KA and AK) was registered with trial number ISRCTN54792066 and has received funding from the Big Lottery Fund and the Wellcome Trust. In Eastern India, the Ekjut trial (covering NN and PT) was registered with trial number ISRCTN21817853 and funded by the Health Foundation. The Dhanusha (Nepal) trial (covering DSM, NS, BPS, and CS) was registered with trial number ISRCTN87820538 and has received funding from: the UBS Optimus Foundation; USAID; DfID and the Wellcome Trust. The City Initiative for Newborn Health trial led by SNEHA (Mumbai, India) was registered with trial number ISRCTN96256793 and has received funding from the ICICI Foundation for Inclusive Growth and the Wellcome Trust. GA, NSM, and DO are currently funded by The Wellcome Trust (091561/Z/10/Z). The funders played no part in the development of the article or the decision to submit it for publication.

Provenance and peer review

Not commissioned; externally peer reviewed.

For peer review only

REFERENCES:

1. World Health Organisation and UNICEF. Countdown 2015: Building a future for women and Children, the 2012 Report. http://countdown2015mnch.org/documents/2012Report/2012-Complete.pdf [Accessed 9th November 2013]
2. Campbell OM, Graham WJ. Strategies for reducing maternal mortality: getting on with what works. *The Lancet* 2006; 368: 1284-1299.
3. Ronsmans C, De Brouwere V, Dubourg D, *et al*.. Measuring the need for life-saving obstetric surgery in developing countries. *BJOG* 2004; 111: 1027-30.
4. World Health Organization. Appropriate technology for birth. *The Lancet* 1985; 2: 436-7.
5. Betrán AP, Merialdi M, Lauer JA, *et al*. Rates of caesarean section: analysis of global, regional and national estimates. *Paediatric and Perinatal Epidemiology* 2007; 21: 98-113.
6. Cavallaro FL, Cresswell JA, França GVA, *et al*. Trends in Caesarean delivery by country and wealth quintile: cross-sectional surveys in southern Asia and sub-Saharan Africa. *WHO Bulletin* 2013; 91: 897-972.
7. Souza JP, Gülmezoglu A, Lumbiganon P, *et al*. WHO Global Survey on Maternal and Perinatal Health Research Group. Caesarean section without medical indications is associated with an increased risk of adverse short-term maternal outcomes: the 2004–2008 WHO Global Survey on Maternal and Perinatal Health. *BMC Medicine* 2010; 8:71.
8. Kyu HH, Shannon HS, Georgiades K, Boyle MH. Caesarian delivery and neonatal mortality rates in 46 low- and middle-income countries: a propensity score matching and meta-analysis of demographic health survey data. *International Journal of Epidemiology* 2013; 1-11.
9. Gibbons L, Belizán JM, Lauer JA, *et al*. The global numbers and costs of additionally needed and unnecessary caesarean sections performed per year: overuse as a barrier to universal coverage. World Health Report (2010), Background paper. Geneva: World Health Organization.
10. Mitra SN, Nawab Ali M, Islam S, *et al*. Bangladesh Demographic and Health Survey, 1993-1994. Calverton, Maryland: National Institute of Population Research and Training (NIPORT), Mitra and Associates, and Macro International Inc, 1994.
11. National Institute of Population Research and Training (NIPORT), Mitra and Associates, and ICF International. Bangladesh Demographic and Health Survey 2011. Dhaka, Bangladesh and Calverton, Maryland, USA: NIPORT, Mitra and Associates, and ICF International, 2013.
12. International Institute for Population Sciences (IIPS) and Macro International. National Family Health Survey (NFHS 1), 1992–93. India, Mumbai: IIPS, 1995.
13. International Institute for Population Sciences (IIPS), Macro International. National Family Health Survey (NFHS 3), 2005–06; India: Volume I. Mumbai: IIPS, 2007.
14. Pradhan A, Aryal RH, Regmi G, *et al*. Nepal Family Health Survey 1996. Kathmandu, Nepal and

For peer review only - http://bmjopen.bmj.com/site/about/guidelines.xhtml

Calverton, Maryland: Ministry of Health [Nepal], New ERA, and Macro International Inc, 1997.

15. Ministry of Health and Population (MOHP) [Nepal], New ERA, and ICF International Inc. Nepal Demographic and Health Survey 2011. Kathmandu, Nepal: Ministry of Health and Population, New ERA, and ICF International, Calverton, Maryland, 2012.
16. Ajeet S, Nandkishore K. The boom in unnecessary caesarean surgeries is jeopardizing women's health. *Health Care Women International* 2013; 34: 513-521.
17. Sreevidya S, Sathiyasekaran BW. High caesarean rates in Madras (India): a population-based cross-sectional study. *BJOG* 2003; 110: 106-111.
18. Koblinksi M, Anwar I, Mridha MK, *et al*. Reducing maternal mortality and improving maternal health: Bangladesh and MDG 5. *J Health Popul Nutr* 2008; 26: 280-294.
19. Leone T, Padmadas SS, Matthews Z. Community factors influencing rising caesarean section rates in developing countries: an analysis of six countries. *Soc Sci Med* 2008; 67: 1236-46.
20. Mazzoni A, Althabe A, Liu NH, *et al*. Women's preference for caesarean section: a systematic review and meta-analysis of observational studies. *BJOG* 2011; 118: 391-399.
21. Tripathy P, Nair N, Barnett S, et al. Effect of a participatory intervention with women's groups on birth outcomes and maternal depression in Jharkhand and Orissa, India: A cluster-randomised controlled trial. *Lancet* 2010; 375: 1182–1192.
22. Azad K, Barnett S, Banerjee B, *et al*. Effect of scaling up women's groups on birth outcomes in three rural districts in Bangladesh: a cluster-randomised controlled trial. *Lancet* 2010; 375: 1193–1202.
23. Fottrell E, Azad K, Kuddus A, *et al*. The effect of increased coverage of participatory women's groups on neonatal mortality in Bangladesh: a cluster-randomised trial. *JAMA Pediatrics* 2013; 167: 816-825.
24. Shah More N, Bapat U, Das S, *et al*. Cluster-randomised controlled trial of community mobilisation in Mumbai slums to improve care during pregnancy, delivery, postpartum and for the newborn. *Trials* 2008; 9:7.
25. Shrestha BP, Bhandari B, Manandhar DS, *et al*. Community interventions to reduce child mortality in Dhanusha, Nepal: study protocol for a cluster randomized controlled trial. *Trials* 2011; 12: 136.
26. Prost A, Colbourn T, Seward N, *et al*. Women's groups practising participatory learning and action to improve maternal and newborn health in low-resource settings: a systematic review and meta-analysis. *Lancet* 2013; 381: 1736–46.
27. Schmidt J-0, Ensor T, Hossain A, Khan S. Vouchers as demand side financing instruments for health care: A review of the Bangladesh maternal voucher scheme. *Health Policy* 2010; 96: 98–107.
28. Ahmed S, Khan MM. A maternal health voucher scheme: what have we learned from the

For peer review only

demand-side financing scheme in Bangladesh? *Health Policy & Planning* 2011; 6:25–32.

29. Lim SS, Dandona L, Hoisington JA, *et al*. India's Janani Suraksha Yojana, a conditional cash transfer programme to increase births in health facilities: an impact evaluation**.** *Lancet* 2010; 375: 209-23.
30. Shah More N, Alcock G, Bapat U, *et al*. Tracing pathways from antenatal to delivery care for women in Mumbai, India: cross-sectional study of maternity in low-income areas. International Health 2009; 1: 71–77.
31. Government of India, Ministry of Health & Family Welfare. Janani Suraksha Yojana: Guidelines for Implementation, 2006. http://www.mohfw.nic.in/NRHM/./guidelines/JSY_guidelines_09_06.pdf
32. Witter S, Khadka S, Nath H, Tiwari S. The national free delivery policy in Nepal: early evidence of its effects on health facilities. *Health Policy and Planning* 2011; 26: ii84–ii91.
33. Holgado-Tello FP,Chacón–Moscoso S, Barbero-Garcia I, Vila-Abad E. Polychoric versus Pearson correlations in exploratory and confirmatory factor analysis of ordinal variables. *Quality & Quantity* 2010; 44 (1): 153-166.
34. Rabe-Hesketh S, Skrondal A, Pickles A. Maximum likelihood estimation of limited and discrete dependent variable models with nested random effects. *Journal of Econometrics* 2005; 128: 301-323.
35. StataCorp. 2013. *Stata Statistical Software: Release 13*. College Station, TX: StataCorp LP.
36. Potter J, Berquó E, Perpétuo I, *et al*. Unwanted Caesarean Sections among Public and Private Patients in Brazil: Prospective Study. *BMJ* 2001; 323: 1155-8.
37. Kamal SMM. Preference for institutional delivery and caesarean sections in Bangladesh. *J Health Popul Nutr* 2013; 31: 96-109.
38. Randive B, Diwan V, De Costa A. India's Conditional Cash Transfer Programme (the JSY) to Promote Institutional Birth: Is There an Association between Institutional Birth Proportion and Maternal Mortality? *PLoS ONE* 2013; 8(6): e67452. doi:10.1371/journal.pone.0067452
39. Chatterjee S, Laxminarayan R. Costs of surgical procedures in Indian hospitals. *BMJ Open* 2013; 3: e002844.
40. Parkhurst JO, Rahman SA. Life saving or money wasting? Perceptions of caesarean sections among users of services in Rural Bangladesh. *Health Policy* 2007; 80: 392-401.
41. Khan R, Blum LS, Sultana M, et al. An Examination of Women Experiencing Obstetric Complications Requiring Emergency Care: Perceptions and Sociocultural Consequences of Caesarean Sections in Bangladesh. *J Health Popul Nutr 2012*; 30: 159-171.
42. Pomeroy A, Koblinksy M, Alva S. Private Delivery Care in Developing Countries: Trends and Determinants. DHS Working document 2010; No.76. http://dhsprogram.com/pubs/pdf/WP76/WP76.pdf [Accessed 3rd March 2014].

For peer review only - http://bmjopen.bmj.com/site/about/guidelines.xhtml

43. Sufang G, Padmadas SS, Fengmin Z et al. Delivery settings and caesarean section rates in China. *Bull WHO* 2007; 85: 733-820.

44. Lo JC. Patients' attitudes vs. physicians' determination: implications for cesarean sections. *Soc Sci Med* 2003; 57: 91-6.

45. McCourt C, Weaver J, Statham H, *et al.* Elective cesarean section and decision making: a critical review of the literature. *Birth* 2007; 34: 65-79.

46. Ghetti C, Chan BKS, Guise J-M. Physicians' responses to patient-requested caesarean delivery. *Birth* 2004; 31: 280-4.

47. Renfrew MJ, McFadden A, Bastos MH, *et al*. Midwifery and quality care: findings from a new evidence-informed framework for maternal and newborn care. *Lancet* 2014 http://dx.doi.org/10.1016/ S0140-6736(14)60789-3.

48. Mavalankar D, Sankara Raman P, Vora K. Midwives of India: missing in action. *Midwifery* 2011; 27: 700-6.

For peer review only - http://bmjopen.bmj.com/site/about/guidelines.xhtml

Table 1: Characteristics of studies and populations

Study (Country)	Bangladesh (rural)	India (rural)	Nepal (rural)	India (informal urban settlementsurban)
**Location**	Three districts: Bogra, Maulvibazaar and Faridpur	Three districts of Jharkhand and Odisha: Keonjhar, West Singhbhum and Saraikela	Dhanusha district (Terai)	Mumbai slums
**Period**	2005 – 2011	2005-2008	2008-2011	2006-2009
**Estimated population**	532,900	114,000	240,000	283,000
**Cluster characteristics**	Villages making up a union	8-10 villages with residents classified as Scheduled Tribe or Other Backward Class	Village Development Committee	Slum areas in six municipal wards of Mumbai
**Method of cluster identification**	Purposive sampling of three districts and clusters within districts	Purposive sampling of three districts and clusters within districts	Random sampling of 60 clusters from a list of 79 suitable clusters in one district	92 clusters in six municipal wards identified using municipal documents, surveys, discussions with key informants, and site visits. Random selection of 48 clusters for randomized allocation.
**Clusters, n**	9	18	30	24
**Cluster and individual follow-up**	All clusters followed up<br>Interviews completed after 82% of identified births in control areas in Phase 1, and 99% of births in Phase 2	All clusters followed up<br>Interviews completed after 98% of identified births	All clusters followed up	All clusters followed up<br>Interviews completed after 83% of identified births
**Maternal mortality ratio**	254.3	668.1	Unknown	206.2
**Health facilities available in control areas**	Public facilities: District Hospitals; Maternal and Child Welfare Centres; Upazilla Health Complexes. Private facilities: small-to-medium-size clinics; BRAC (NGO) facilities where deliveries do not take place; larger private hospitals with and without Comprehensive emergency obstetric care (CEmOC)	Public facilities: District Hospitals; Primary Health Centres (PHCs) in which deliveries can notionally take place but that are not usually equipped for CEmOC; Community Health Centres (CHCs) acting as referral centres for PHCs, covering a population of around 80,000 with EmOC facilities; district hospitals. Private and charitable facilities: medium-sized	Public facilities: three Primary Health Care Centres, three Health Posts and 24 Sub-Health Posts, none of which are equipped for CEmOC. These health facilities refer to the public Zonal Tertiary Hospital and various private providers in the district headquarters and nearby medical college, which have facilities for caesarean	Public facilities: Municipal tertiary hospitals, general hospitals, and maternity homes. Private facilities: specialty hospitals, general hospitals, and maternity homes.

Formatted: Font: 9 pt

For peer review only

For peer review only - http://bmjopen.bmj.com/site/about/guidelines.xhtml

facilities.	missionary hospitals with EmOC facilities.	sections.

For peer review only

For peer review only - http://bmjopen.bmj.com/site/about/guidelines.xhtml

Table 2: Characteristics of institutional births and caesarean deliveries by location

	Births	Institutional births						Caesarean section births			
		All		Public facility		Private/charitable facility		Public facility		Private/charitable facility	
	(n)	(n)	(% all births)	(n)	(% institutional births)	(n)	(% institutional births)	(n)	(% deliveries in public facilities)	(n)	(% deliveries in private facilities)
**Bangladesh (rural)**	21,560	4,592	21%	2,053	45%	2,539	55%	589	29%	1,852	73%
**India (rural)**	8,541	1,816	21%	421	23%	1,395	77%	64	15%	66	5%
**India (informal urban ~~urban~~settlements)**	10,236	9,259	90%	5,489	59%	3,770	41%	811	15%	697	18%
**Nepal (rural)**	4,931	1,586	32%	1,424	90%	162	10%	201	14%	48	30%

Formatted Table

Formatted: Left, Indent: Left: 0", Add space between paragraphs of the same style

* Nepal numbers weighted using women's probability of selection within each cluster, scaled to total number of institutional births. (Unweighted Nepal totals: 4,990 births and 1,586 institutional births)

For peer review only

For peer review only - http://bmjopen.bmj.com/site/about/guidelines.xhtml

Table 3: Mutually adjusted associations of type of facility of delivery and other selected determinants with caesarean section delivery, by location

	Bangladesh (rural)		India (rural)		India (informal urban settlements~~urban~~)		Nepal (rural)*	
	ORs (95% CIs)		ORs (95% CIs)		ORs (95% CIs)		ORs (95% CIs)	
	Crude	Adjusted	Crude	Adjusted	Crude	Adjusted	Crude	Adjusted
**Health facility characteristics**								
Public health facility (ref)								
Private health facility	6.82 (5.96,7.81)	5.91 (5.15,6.78)	0.39 (0.25,0.61)	0.46 (0.29,0.73)	1.36 (1.21,1.52)	1.22 (1.09,1.38)	2.42 (1.48,3.94)	2.37 (1.62,3.44)
**Pregnancy and delivery characteristics**								
4+ antenatal care visits (fewer or none = ref)**		1.46 (1.26,1.69)		1.49 (0.96,2.32)		1.06 (0.93,1.21)		1.92 (1.43,2.58)
Birth order								
1 (ref)								
2		0.95 (0.80,1.13)		1.25 (0.80,1.95)		0.65 (0.56,0.75)		1.41 (1.02,1.94)
3		1.19 (0.94,1.52)		0.36 (0.15,0.87)		0.60 (0.50,0.71)		0.58 (0.37,0.92)
4+		1.17 (0.90,1.53)		0.36 (0.15,0.85)		0.39 (0.32,0.48)		1.00 (0.54,1.84)
Serious complications in pregnancy/delivery*** (no complications=ref)		0.87 (0.76,1.00)		1.77 (1.17,2.67)		1.71 (1.39,2.11)		4.87 (2.51,9.47)
Multiple birth (ref=single)		0.93 (0.65,1.32)		1.57 (0.52,4.75)		3.01 (2.14,4.23)		3.42 (1.77,6.61)
**Maternal characteristics**								
Age (years)								
15-24 (ref)								
25-34		1.12 (0.94,1.34)		1.81 (1.14,2.86)		1.45 (1.27,1.66)		1.52 (0.63,1.94)
35+		1.10		2.11		1.79		1.55

For peer review only - http://bmjopen.bmj.com/site/about/guidelines.xhtml

	Bangladesh (rural) ORs (95% CIs)		India (rural) ORs (95% CIs)		India (informal urban settlements~~urban~~) ORs (95% CIs)		Nepal (rural)* ORs (95% CIs)	
	Crude	Adjusted	Crude	Adjusted	Crude	Adjusted	Crude	Adjusted
		(0.77,1.58)		(0.63,7.07)		(1.27,2.53)		(0.55,1.47)
Maternal education****								
No formal education (ref)								
Primary education		1.07		0.98		1.13		0.90
		(0.83,1.38)		(0.39,2.47)		(0.85,1.49)		(0.59,1.39)
Secondary education		1.44		1.11		1.22		1.37
		(1.13,1.84)		(0.68,1.80)		(1.04,1.42)		(0.97,1.94)
Bachelor's degree or higher		2.44		1.20		1.62		-
		(1.52,3.92)		(0.43,3.31)		(1.30,2.02)		-
Household wealth quintile								
1st (poorest, ref)								
2nd		1.31		1.61		1.14		1.11
		(1.06,1.63)		(0.69,3.75)		(0.77,1.68)		(0.63,1.94)
3rd		1.41		1.36		1.13		0.90
		(1.10,1.82)		(0.58,3.21)		(0.95,1.33)		(0.55,1.47)
4th (wealthiest)		1.36		2.16		1.50		0.99
		(1.09,1.70)		(0.87,5.33)		(1.27,1.78)		(0.52,1.90)
Year of delivery								
2004-2006 (ref: BD, IN)								-
2007-2009 (ref: NP rural)		0.90		0.90		1.14		-
		(0.74,1.10)		(0.61,1.33)		(1.09,1.38)		-
2010-2012		1.15		-		-		1.29
		(0.94,1.41)		-		-		(0.91,1.82)
N	4,592	4,592	1,816	1,816	9,259	9,259	1,586	1,586

* Nepal numbers weighted using women's probability of selection within each cluster, scaled to total number of institutional births.

** At least one visit with skilled provider

***Includes: symptoms of eclampsia (fits, seizures, convulsions, or unconsciousness during pregnancy or delivery); reduced or no fetal movement; labour lasting more than 24 hours.

****For the Nepal data, two respondents with bachelor's degrees (2 respondent total, 1 delivering in institution) were combined with respondents with secondary education.

For peer review only

For peer review only - http://bmjopen.bmj.com/site/about/guidelines.xhtml

All analyses additionally adjusted for survey design using fixed effect of stratum and random effect of cluster.

For peer review only

Table 4: Interactive associations between type of delivery facility, maternal education, and caesarean delivery, by location

	**Bangladesh (rural)**		**India (rural)**		**India (informal urban settlements~~urban~~)**		**Nepal (rural)***	
	(n)	AOR, 95% CI	(n)	AOR, 95% CI	(n)	AOR, 95% CI	(unweighted n)	AOR, 95% CI
Public facility - no maternal education (ref)	202	-	109	-	1,396	-	822	-
Public facility - primary education	448	1.07	17	0.92	357	0.88	196	1.04
		(0.76,1.50)		(0.18,4.77)		(0.61,1.27)		(0.65,1.65)
Public facility - secondary education	202	1.63	604	1.09	920	1.1	413	1.45
		(1.18,2.24)		(0.52,2.26)		(0.91,1.33)		(1.04,2.02)
Public facility - bachelor's degree	448	4.58	100	1.55	180	1.06	-	-
		(2.24,9.38)		(0.41,5.89)		(0.76,1.47)		-
Private facility - no education	332	7.24	109	0.46	1396	0.91	100	2.83
		(4.82,10.87)		(0.21,1.01)		(0.70,1.19)		(1.73,4.61)
Private facility - primary education	578	7.58	17	0.47	357	1.52	16	0.86
		(5.36,10.71)		(0.14,1.58)		(0.99,2.33)		(0.14,5.21)
Private facility - secondary education	1,102	9.03	274	0.52	3,382	1.3	39	3.01
		(6.57,12.41)		(0.24,1.12)		(1.06,1.60)		(1.71,5.30)
Private facility - bachelor's degree	41	11.24	21	0.4	354	2.1	-	-
		(6.37,19.85)		(0.08,2.00)		(1.61,2.75)		-
N	3,353		1,251		8,342		1586	
p-value**	0.021		0.792		0.001		0.394	

All results adjusted for number of antenatal care visits, parity, medical indication for caesarean, multiple birth, maternal age (10-yr group), household assets, stratum, and cluster (random effect).
* Nepal numbers weighted using women's probability of selection within each cluster, scaled to total number of institutional births.

** p-value from -2 log-likelihood test comparing nested models with and without interaction terms

For peer review only

For peer review only - http://bmjopen.bmj.com/site/about/guidelines.xhtml

BMJ Open

Prevalence and determinants of caesarean section in private and public health facilities in underserved South Asian communities: cross-sectional analysis of data from Bangladesh, India, and Nepal

Journal:	*BMJ Open*
Manuscript ID:	bmjopen-2014-005982.R2
Article Type:	Research
Date Submitted by the Author:	29-Oct-2014
Complete List of Authors:	Neuman, Melissa; UCL Institute for Global Health,<br>Alcock, Glyn; UCL Institute for Global Health,<br>Azad, Kishwar; Diabetic Association of Bangladesh, Perinatal Care Project<br>Kuddus, Abdul; Diabetic Association of Bangladesh, Perinatal Care Project<br>Osrin, David; UCL Institute for Global Health,<br>Shah More, Neena; Society for Nutrition, Education and Health Action (SNEHA),<br>Nair, Nirmala; Ekjut,<br>Tripathy, Prasanta; Ekjut,<br>Sikorski, Catherine; UCL Institute for Global Health,<br>Saville, Naomi; UCL Institute for Global Health,<br>Sen, Aman; Mother and Infant Research Activities (MIRA),<br>Colbourn, Timothy; UCL Institute for Global Health,<br>Houweling, Tanja; Erasmus MC University Medical Center, Department of Public Health<br>Seward, Nadine; UCL Institute for Global Health,<br>Manandhar, Dharma; Mother and Infant Research Activities (MIRA),<br>Shrestha, Bhim; Mother and Infant Research Activities (MIRA),<br>Costello, Anthony; UCL Institute for Global Health,<br>Prost, Audrey; UCL Institute for Global Health,
**Primary Subject Heading**:	Global health
Secondary Subject Heading:	Epidemiology, Obstetrics and gynaecology
Keywords:	Epidemiology < TROPICAL MEDICINE, OBSTETRICS, PUBLIC HEALTH

SCHOLARONE™ Manuscripts

Prevalence and determinants of caesarean section in private and public health facilities in underserved South Asian communities: cross-sectional analysis of data from Bangladesh, India, and Nepal

Melissa Neuman[1], Glyn Alcock[1], Kishwar Azad[4], Abdul Kuddus[4], David Osrin[1], Neena Shah More[3], Nirmala Nair[5], Prasanta Tripathy[5], Catherine Sikorski[1], Naomi Saville[1], Aman Sen[2], Tim Colbourn[1], Tanja AJ Houweling[6], Nadine Seward[1], Dharma S Manandhar[2], Bhim P Shrestha[2], Anthony Costello[1] and Audrey Prost[1]

Author affiliations:

[1] Institute for Global Health, University College London, 30 Guilford Street, London WC1N 1EH, UK

[2] Mother and Infant Research Activities (MIRA), YB Bhavan, Thapathali, GPO Box 921, Kathmandu, Nepal

[3] Society for Nutrition, Education and Health Action (SNEHA), Urban Health Centre, Shahunagar, Dharavi, Mumbai 400017, Maharashtra, India

[4] Perinatal Care Project, Diabetic Association of Bangladesh, 122 Kazi Nazrul Islam Avenue, Dhaka 1000, Bangladesh

[5] Ekjut, Plot 556B, Potka, Chakradharpur, West Singhbhum, Jharkhand, India

[6] Department of Public Health, Erasmus MC University Medical Center Rotterdam, P.O. Box 2040, 3000 CA Rotterdam, The Netherlands

Proposed running head: Caesarean sections in private vs. public health facilities in South Asia

Word count: 4393

Figures and tables: 0 figures and 4 tables

For peer review only

For peer review only - http://bmjopen.bmj.com/site/about/guidelines.xhtml

ABSTRACT (Max: 300 words; Current: 300)

Objectives: To describe the prevalence and determinants of births by caesarean section in private and public health facilities in underserved communities in South Asia.

Design: Cross-sectional study.

Setting: 81 community-based geographical clusters in four locations in Bangladesh, India and Nepal (three rural, one urban).

Participants: 45,327 births occurring in the study areas between 2005 and 2012.

Outcome measures: proportion of caesarean section delivery by location and type of facility; determinants of caesarean section delivery by location.

Results: Institutional delivery rates varied widely between settings, from 21% in rural India to 90% in urban India. The proportion of private and charitable facility births delivered by caesarean section was 73% in Bangladesh, 30% in rural Nepal, 18% in urban India, and 5% in rural India. The odds of caesarean section were greater in private and charitable health facilities than in public facilities in three of four study locations, even when adjusted for pregnancy and delivery characteristics, maternal characteristics, and year of delivery (Bangladesh AOR: 5.91, 95% CI: 5.15-6.78; Nepal AOR: 2.37, 95% CI: 1.62-3.44; urban India AOR: 1.22, 95% CI: 1.09-1.38). We found significant interactive associations between maternal education and private facility delivery in two of four locations, with highly educated women particularly likely to deliver by caesarean in private facilities in both urban India (AOR 2.10; 95% CI: 1.61, 2.75) and rural Bangladesh (AOR 11.09, 95% CI: 6.28, 19.57).

Conclusions:

Our results lend support to the hypothesis that increased caesarean section rates in these South Asian countries may be driven in part by the private sector. They also suggest that preferences for caesarean delivery may be higher among highly educated women, and that individual- and provider-level factors interact in driving caesarean rates higher. Rates of caesarean section in the private sector, and their maternal and neonatal health outcomes, require close monitoring.

For peer review only - http://bmjopen.bmj.com/site/about/guidelines.xhtml

For peer review only

Article summary

Article focus

- Caesarean sections are on the rise in South Asia. Research suggests that private health facilities may be contributing to this by conducting caesareans over and above clinical need.
- Literature from other settings indicates that increases in caesarean sections are shaped by both supply and demand pressures: providers often have financial incentives to intervene surgically, and women of higher socio-economic status are also more likely to want caesareans.
- This study examined the prevalence and determinants of caesarean sections in private and charitable facilities compared with public health facilities for over 45,000 births in underserved communities of rural Nepal, rural Bangladesh, rural and urban India.

Key messages

- Caesarean sections were more common in private than in public health facilities in three of four study locations (urban India, rural Nepal, and rural Bangladesh).
- We found significant interactive associations between maternal education and private facility delivery on caesarean in Bangladesh (both private and public facilities) and urban India (private facilities only).
- Future research should monitor caesarean section rates in the private sector in South Asia and subsequent maternal and neonatal health outcomes

Strengths and limitations

- This study had a large sample size and focused on underserved communities, which are a priority for public health interventions in South Asia.
- Our data were not nationally representative, which limits the generalisability of our findings. Other limitations included small denominators and missing data for some variables. We did not have data on some known predictors of caesarean section, which would have enhanced the completeness of our determinants analysis.

For peer review only - http://bmjopen.bmj.com/site/about/guidelines.xhtml

INTRODUCTION

Access to comprehensive emergency obstetric care, including caesarean section, is key to preventing the estimated 287,000 maternal and 2.9 million neonatal deaths that occur worldwide every year.[1 2] Although debate continues about how to quantify the need for life-saving obstetric surgery, a 1985 World Health Organization (WHO) report suggested that the optimal population range for caesarean section rates is between 5% and 15%, and this endures as a reference.[3 4] Caesarean section rates are increasing worldwide, albeit unequally: a recent analysis of Demographic and Health Survey (DHS) data in 26 South Asian and sub-Saharan African countries found that rates were highest among the 'urban rich' in all countries, and lowest among the 'rural poor' in 18.[5] In all countries, fewer than 5% of mothers in the poorest wealth quintile delivered by caesarean.[6]

Caesarean sections conducted without clinical need can have adverse consequences for mothers and children. A 2008 WHO survey of 373 facilities across 24 countries found that unnecessary caesareans were associated with an increased risk of maternal mortality and serious outcomes for mothers and newborn infants, compared with spontaneous vaginal delivery.[7] Recent ecological analyses also highlighted strong associations between caesarean delivery and increased neonatal mortality in countries with low and medium caesarean section rates.[8] Unnecessary caesareans lead to considerable costs for families and health systems: an estimated 6.2 million unnecessary procedures were performed in 2008, costing approximately US$ 2.32 billion. [9]

Several South Asian countries have recorded substantial increases in caesarean section rates over the past decade. In Bangladesh, rates rose from 2% (2000) to 17% (2011); in India, from 3% (1992) to 11 % (2006); and in Nepal, from 1% (2000) to 5% (2011).[10-5] Several studies in these countries have raised concerns about high caesarean rates in private facilities, and a recent DHS analysis speculated that national increases in caesarean section rates in South Asian countries could be driven in part by higher rates among deliveries in private sector facilities[.6 16-18] Literature from other settings indicates that increases in caesarean sections are shaped by both supply and demand pressures: providers often have financial incentives to intervene surgically, and women of higher socio-economic status are also more likely to desire caesareans.[19 20]

There are few large, recent community-based studies from South Asia quantifying differences in caesarean section rates between public and private facilities. Such studies are necessary in order to examine whether increases in caesareans in these settings are indeed likely to be driven by the private sector, demand from wealthier and more educated mothers, or a combination of the two. We conducted a cross-sectional analysis of data from Bangladesh, India, and Nepal to explore the prevalence and determinants of caesarean section delivery by type of facility and location, and to examine the

For peer review only - http://bmjopen.bmj.com/site/about/guidelines.xhtml

interactions between maternal education and place of delivery on prevalence of caesarean section.

METHODS

Study populations

We used data collected through vital events surveillance systems established during four cluster-randomised controlled trials conducted between 2005 and 2011. The trials were done with communities that can be considered socio-economically disadvantaged: in Bangladesh and Nepal, they took place in four rural, underserved districts (Bogra, Maulvibazaar, and Faridpur in Bangladesh, and the Terai district of Dhanusha, Nepal); in rural India, most participants were from Scheduled Tribes in two states of eastern India (Jharkhand and Odisha); in urban India, data came from slum communities in Mumbai. **Table 1** describes the characteristics of each study and its population, including the background maternal mortality ratio and types of facilities present in the study areas. The original trials were designed to evaluate the impact of participatory women's groups on maternal and neonatal health outcomes.[21-25] We used data from the control areas of these trials only, because the women's group intervention led to changes in mortality and practices in several locations.[26]

Health system contexts

All three study countries have experienced substantial increases in institutional delivery rates over the last two decades: births in health facilities increased from 4% (1993) to 29% (2011) in Bangladesh; from 26% (1992-3) to 47% (2007-8) in India; and from 8% (1996) to 35% (2011) in Nepal.[10-15] All three countries have implemented incentive schemes to promote institutional delivery, though these have varying coverage. In 2010, Bangladesh's pilot maternity voucher scheme reached an estimated 10.4 million people across 31 sub-districts, around 7% of the country's population.[27 28] Mothers receive a cash incentive for antenatal care and delivery in a public or private facility, or at home with a skilled birth attendant. Both government and private facility staff also receive cash incentives, including 3000 Bangladeshi Taka (US $38.5) for a caesarean section and 300 Taka for a normal delivery.[28] This maternity incentive scheme was operational in two of the three districts (Faridpur and Maulvibazaar) covered by our study. All three districts had public facilities including District Hospitals, Maternal and Child Welfare Centres, and Upazilla Health Complexes. Private facilities included a number of small-to-medium-size clinics, BRAC (NGO) facilities, and larger private hospitals.

In India, the *Janani Suraksha Yojana* (JSY) maternity incentive scheme entitles women in rural areas of high focus states, including those in this study, to 1400 Indian Rupees ($22.4) after delivering in a government or accredited private health facility. Local community health volunteers called Accredited Social Health Activists (ASHAs) also receive 600 Indian Rupees ($9.6) for identifying pregnant women and helping them get to a health facility.[29] Although the rural Indian data included in this study were

For peer review only - http://bmjopen.bmj.com/site/about/guidelines.xhtml

collected between 2005 and 2008, the JSY was only operational in the study areas from 2008 onwards and its impact is unlikely to be reflected here. The Indian urban area included in our study spanned informal settlements (slums) with a wealth of public and private providers.[30] Mothers with Below Poverty Line cards in such areas are eligible for JSY and can receive Rs 500 towards the costs of delivering in a health facility.[31] Nepal began a safe delivery incentive scheme in 2005 and free deliveries have been available in government facilities since 2009 through the *Aama Surakshya* programme.[32] Dhanusha district, from which data for this study come, has one zonal tertiary hospital and a private medical college hospital equipped for c-sections, and a variety of small public and private health facilities without comprehensive obstetric care.

Data collection

All data were collected using surveillance systems to monitor births and deaths prospectively.[21-25] In all study locations, a female community-based key informant reported births and deaths in her area, which covered a population ranging from 250 to 350. A trained interviewer then verified these reports and paid the informant an incentive for each correct identification. In Bangladesh, rural and urban India, the interviewer administered a structured questionnaire to all eligible mothers around six weeks after delivery; in Nepal, all births in the study area were registered, and interviews were conducted on all births in small clusters and on a random sample of 10 births per month in the larger clusters. In each study location, mothers were interviewed using a questionnaire to collect information about events in the antenatal, delivery and postnatal periods. In each trial and in this subsequent study, participants were women of reproductive age (15-49) who delivered in the study area during the data collection period, and who consented to be interviewed six weeks after delivery, and their infants.

Study sample

The initial sample for this analysis included 46,393 births in Mumbai, rural India, rural Bangladesh, or rural Nepal, of whom 17,565 (38%) were delivered in health care facilities. We excluded the 2348 births occurring outside Mumbai, because we collected limited information on delivery location for these. Additionally, 223 deliveries had missing data on form of delivery (caesarean or vaginal), 602 had missing or implausible values for mother’s age, and 98 were missing information on other maternal characteristics (educational attainment, household assets, number of prior pregnancies, number of antenatal visits, severe problems in pregnancy or delivery). 143 births had missing or incomplete information on place of delivery. The final analytic sample included 45,327 births (97.7% of the initial sample).

For peer review only - http://bmjopen.bmj.com/site/about/guidelines.xhtml

Measures used

The primary outcome in these analyses was caesarean section delivery, identified by self-report from the mother or another household member around six weeks after giving birth. The main covariate of interest was the type of delivery facility, coded as public (e.g. district hospitals), private (individual private clinics or hospitals), or NGO/charitable (e.g. Christian missionary hospitals in rural India; BRAC clinics in Bangladesh). We grouped private and NGO/charitable facilities together due to the small number of births in NGO/charitable facilities (6% of institutional deliveries in Bangladesh, 7% in rural India, and 0 in urban India and rural Nepal; data on request). Additional covariates in the models included measures of the characteristics of the pregnancy and delivery, maternal socio-demographic characteristics, and the location in which the delivery occurred. We created an indicator variable to identify women experiencing serious problems during pregnancy or delivery, with women reporting symptoms of pre-eclampsia (blurred vision or swelling of face and hands), symptoms of eclampsia (fits or seizures during pregnancy or delivery), hæmorrhage during delivery, or labour lasting more than 24 hours considered to have serious complications. Other characteristics of the pregnancy and delivery considered in the analysis included full utilisation of antenatal care, number of prior pregnancies, and multiple pregnancy. Full utilization of antenatal care was defined as four or more visits to antenatal care, with at least one visit to a skilled provider. Number of pregnancies was entered as an ordered categorical variable, with categories of one (first), two, three, or four or more pregnancies.

Maternal socio-demographic characteristics included in the models were: mother's age at delivery, her educational attainment, and household assets. Mother's age was entered into the model as a categorical measure in 10-year groups. Educational attainment was entered as a categorical variable using the following categories: no formal education, primary education, secondary education, or bachelor's degree or higher. To develop an asset index, we used polychoric factor analysis on data on common assets and amenities found in the mother's household, and grouped the resulting factor scores into quartiles.[33] Assets and amenities included electricity, radio or cassette player, electric fan (Bangladesh and India only), television, refrigerator (Bangladesh and India only), telephone (Bangladesh and Nepal only), generator (India only), and bicycle. All models were additionally adjusted for location (Bangladesh, India, Nepal), and year of interview in three-year groups. The data were collected in a stratified, cluster-sampled survey, and we accounted for survey design in the analysis using a fixed effect for stratum and a random effect for cluster.

Statistical analysis

We used frequencies to describe caesarean section rates by delivery location at each site. We used the Generalized Linear Latent And Mixed Models (GLLAMM) procedure in Stata 13.1, with adaptive quadrature for binary outcomes, to estimate the crude association between type of delivery facility and

For peer review only - http://bmjopen.bmj.com/site/about/guidelines.xhtml

caesarean section.[34 35] We identified other maternal, pregnancy, and delivery characteristics potentially associated with caesarean section using existing literature, especially studies resulting from the WHO multi-country surveys, and entered these in adjusted models to explore how they modified the association between type of delivery facility and caesarean section, and their individual association with caesarean section. Some South Asian and Latin American studies have detected a strong association between maternal education and caesarean delivery. [7 36 37] We fitted models including indicator variables for each group of mothers by education and type of delivery facility to explore differences in the strength of association between caesarean delivery and private facility by mother's education. To account for the sampling procedure used in rural Nepal, models were adjusted using pweights (probability of selection within cluster); these weights were rescaled to reflect the total number of institutional deliveries.

Ethical approval

The trials that provided data for this study received ethical approval from the following committees: the ethics committee of the Diabetic Association of Bangladesh (Perinatal Care Project, Bangladesh Diabetes Somity, BADAS); an independent ethics committee in Jamshedpur, Jharkhand, India; the Nepal Health Research Council; the Municipal Corporation of Greater Mumbai and the Independent Ethics Committee for Research on Human Subjects (Mumbai, India); the ethics committee of the Institute of Child Health, University College London.

RESULTS

Table 2 describes the characteristics of institutional births and caesarean deliveries by location. We analysed data for 45,327 births: 21,560 in rural Bangladesh, 8,541 in rural India, 10,236 in urban India, and 4,931 in rural Nepal. The proportion of women delivering in health facilities varied widely between locations, from 90% in urban India to 21% in rural India There were also large variations in the proportion of women delivering in private/charitable facilities rather than public facilities, with the highest (77%) in rural India and the lowest (10%) in rural Nepal. The proportion of women giving birth by caesarean section in private rather than public facilities also varied widely between settings. In Bangladesh, only 21% of women delivered in a health facility, around half of them in the private/charitable sector, but 73% of private facility births were by caesarean section. In rural Nepal, 30% of the 162 private facility births were by caesarean section. In informal settlements of Mumbai, 15% of public facility deliveries were caesareans, compared with 18% of private facility deliveries. In rural India, caesarean sections were more commonly performed in public health facilities than private or charitable facilities (15% vs. 5%). **Supplemental table 1** describes the characteristics of institutional deliveries (caesarean or non-caesarean) according to type of delivery facility (private or public), maternal

For peer review only - http://bmjopen.bmj.com/site/about/guidelines.xhtml

For peer review only

socio-economic and socio-demographic characteristics, pregnancy and delivery characteristics, and year of delivery.

Table 3 shows crude and adjusted measures of association between type of delivery facility (private or public) and caesarean section for each location. Delivering in a private health facility was associated with an increased odds of caesarean section in all but one location (rural India). The relative odds of a caesarean in a private facility were greatest in Bangladesh (OR: 6.82, 95% CI: 5.96, 7.81), followed by Nepal (OR: 2.42, 95% CI: 1.48, 3.94) and urban India (OR 1.36, 95% CI: 1.21, 1.52). These associations persisted and were only mildly attenuated when adjusted for maternal characteristics, pregnancy and delivery characteristics, and year of delivery (Bangladesh AOR: 5.91, 95% CI: 5.15, 6.78; Nepal AOR: 2.37, 95% CI: 1.62, 3.44; urban India AOR: 1.22, 95% CI: 1.09, 1.38).

Women who had four or more antenatal check-ups were significantly more likely to have a caesarean delivery in rural Bangladesh and Nepal, with positive but non-significant trends in all other locations. Parity was not associated with caesarean delivery in any location. Having a serious health complication during pregnancy and delivery was associated with caesarean delivery in all locations except rural Bangladesh, where we observed a negative association (AOR: 0.87, 95% CI: 0.76, 1.00). Multiple birth was associated with an increased odds of caesarean section only in urban India and in rural Nepal. Higher maternal age was only associated with an increased odds of caesarean section in urban India. Maternal education was only associated with an increased odds of caesarean delivery in rural Bangladesh and urban India, with mothers with secondary education or Bachelor's degrees having higher odds (AOR: 1.44, 95% CI: 1.13, 1.84 and AOR 2.44, 95% CI: 1.52, 3.92 for Bangladesh and AOR: 1.22, 95% CI: 1.04, 1.42 and 1.62, 95% CI: 1.30, 2.02 for urban India, respectively). There were significant positive associations only in rural Bangladesh, with small increases in odds of caesarean section for unit increase in wealth quartile (AOR comparing wealthiest to poorest group in Bangladesh: 1.36, 95% CI: 1.09,1.70), and in urban India, for the wealthiest quartile (AOR: 1.50, 95% CI: 1.27, 1.78).

We found interactive associations between maternal education and private facility delivery in two of four sites, with including an interaction improving model fit in two of the four analyses (p=0.021 in rural Bangladesh and p<0.001 in urban India; **table 4 and supplemental Table 2**). In Bangladesh, there was a pronounced positive educational gradient in caesarean delivery within those who delivered in private facilities, as well as a clear positive association between private facility delivery and caesarean delivery. Women with bachelor's degrees delivering in private facilities had 11 times greater odds of delivering by caesarean (AOR 11.09, 95% CI: 6.28, 19.57) than did women with no formal education delivering in public facilities. Women with no formal education delivering in private facilities had nearly seven times the odds of caesarean compared with the reference group (AOR 6.94, 95% CI: 4.64, 10.39). A similar

For peer review only - http://bmjopen.bmj.com/site/about/guidelines.xhtml

positive gradient was seen among women delivering in public facilities, with women with bachelor's education having four times greater odds of caesarean delivery in public facilities than women with no formal education (AOR 4.55, 95% CI: 2.22, 9.33). There was also an educational gradient among women delivering in private facilities in urban India. Educated mothers delivering in private facilities were more likely to deliver by caesarean than were mothers in other groups (AOR 2.10; 95% CI: 1.61, 2.75). However, there was no apparent gradient by educational attainment among women delivering in public facilities.

DISCUSSION

Our analysis of data from over 45,000 births confirms the findings of other studies identifying differences in caesarean rates between public and private facilities, and suggests that, even in underserved areas in South Asia, caesareans without medical indication are of concern. In three of four locations, rates of caesarean section were higher in private/charitable facilities than in public facilities. The findings from Bangladesh are particularly noteworthy as they show much greater odds of caesarean section in private facilities, concurring with previous analyses.[37] This was also the only location where serious complications in pregnancy and delivery were not associated with caesarean delivery, suggesting that obstetric surgery was performed over and above clinical need.

Our findings confirm the results of earlier studies of the prevalence of caesarean delivery in South Asia, and indicate that high rates can be found in underserved rural areas. In India, a recent analysis of 2010-11 Annual Health Survey (AHS) data from 284 districts in nine States, including Jharkhand, found that the median caesarean section rate in the private sector was 28%, compared with 5% in the public sector.[38] There appear to be strong financial incentives for surgical procedures in the private sector.[39] In a rural, largely indigenous part of eastern India where more mothers delivered in private/charitable facilities than in public facilities, more caesarean sections were performed in the public sector. In this particular setting, our field experience suggests that only women with serious complications would go to a facility and have a caesarean section, especially as JSY was not yet available in the study areas at the time of data collection. Such women are likely to have experienced multiple referrals from either ill-equipped public or private facilities not wanting to take the risk of admission. It is however possible that preference for the private sector has changed in Jharkhand since the advent of JSY, and further disaggregated analyses of Annual Health Survey data would allow a more contemporary exploration of State-level variations in caesarean section rates between public and private sectors.

In Mumbai slums with a high uptake of institutional births and a 60/40 split in favour of the public sector for delivery care, we found no difference in caesarean section rates between the two sectors, which is

For peer review only - http://bmjopen.bmj.com/site/about/guidelines.xhtml

For peer review only

somewhat surprising given the high rates of caesareans observed in the private sector in nine other states of India (excluding Maharashtra). At least two scenarios may be relevant in this setting. First, public sector hospitals are able to provide caesarean section, while smaller private maternity homes may not be able to. Second, the costs of caesarean section borne by private sector providers are higher in Mumbai, and families may be unwilling to bear these additional costs. This provides motivation for private providers not to provide caesareans, and makes it likely that women experiencing complications move into the public sector. The variety of facility options in an urban environment means that mothers are able to choose between different types of facilities, while in rural areas only one provider may be accessible.

Analyses using the most recent Bangladesh DHS found that, in 2011, three in five facility births were delivered by caesarean section. This reflects a historical trend: in 2001-2003, nearly half of deliveries in private facilities in Bangladesh were already by caesarean section.[18] Our data from rural, socio-economically disadvantaged communities in three districts of Bangladesh confirm these population-level findings, but also suggest that high caesarean section rates in private facilities are not merely an issue for wealthy urban mothers. Although some research from Bangladesh suggests that mothers may have a preference for caesarean delivery because of fear of labour pain or a desire to select an auspicious date for the birth, other studies also highlight women's fears of caesarean section and their distrust of health providers who recommend them, mainly because of the high costs associated with the procedure.[37 40 41] A qualitative study involving twenty women who had experienced obstetric complications in Matlab in 2008-9 found that most of the fourteen women who had undergone caesareans had spent over Tk 14,999 (US$ 217) on the procedure, which was approximately one-third of GDP per capita at the time.[41] The lack of association between caesareans and complications in pregnancy or delivery and multiple births, coupled with the high financial incentives given to providers for performing caesarean sections and the requirement for junior doctors to 'practise' their surgical skills, further suggest that obstetric surgery is being used over and above clinical need. A possible explanation for the significant interactive associations between maternal education and caesarean delivery in both public and private facilities in Bangladesh is that well-educated women may be delivering in more expensive or highly rated institutions, which may in turn be more likely to do caesarean sections for financial reasons and if they act as training centres for junior doctors.

In our sample of institutional deliveries from rural Nepal, 16% of facility births were by caesarean, which is higher than the national average: the 2011 Nepal DHS found an overall national caesarean section rate of 5%, with sections more commonly performed for births to highly educated mothers (13%) and mothers in the highest wealth quintile (14%).[26] It is possible that women with complications are more likely to deliver in facilities, and also that, as in Bangladesh, private providers are motivated by financial

For peer review only - http://bmjopen.bmj.com/site/about/guidelines.xhtml

incentives to conduct caesareans more frequently than strictly necessary. Women who can afford to seek care in private facilities may also be more willing or able to pay for caesarean sections, and providers may conduct more of them to increase their income.

A 2010 analysis of DHS data examining the role of the private sector in maternity care in 16 countries found evidence of a trend towards privatisation in delivery care between the 1990s and mid-2000s, but with strong differences between countries, which might reflect the heterogeneous nature of this sector both between and within countries. [42] This DHS study highlighted the need for more context-specific data on the nature of the private sector in low and middle-income countries, and its role in maternity care. Further research might focus on understanding the motivations and experiences of women undergoing caesarean sections in private facilities in South Asian settings, pathways for switching between public and private sectors in the event of obstetric complications, a more comprehensive tally of the financial incentives (official or non-official) that motivate private providers to carry out caesareans in each setting, and the consequences of increased caesarean sections in the private sector for maternal and neonatal health outcomes.

In our study, locations with higher prevalence of caesarean deliveries also showed a positive educational gradient even after adjusting for wealth measured by household assets. Moreover, there was an interactive association between education and type of facility, with highly educated women particularly likely to receive caesarean deliveries in private facilities in Bangladesh and urban India. Literature exploring the determinants of caesarean delivery emphasizes that multiple influences drive a woman's decision to deliver by caesarean. Profit considerations at the facility level may prompt some providers to urge women to receive unnecessary procedures, while women themselves may prefer caesareans for cultural reasons, fear of painful deliveries, or because they believe it to be safer. [36 43-45] Within the client-provider interaction, providers may be more likely to acquiesce to a request for caesarean from a highly educated woman; and such women may be more likely to accept advice from a provider. [19 46] Our findings lend support to the hypothesis that, while provider-level factors are probably partially responsible for the rapid increase in caesarean deliveries, it is also necessary to consider women's own preferences and decision-making processes and how they are shaped by social and cultural factors.

Strengths and limitations

The strengths of the study were its large sample size and focus on underserved communities, which are a priority for public health interventions in South Asia. It had five main limitations. It was not a nationally representative study, and districts or clusters were sampled purposively from previous cRCTs. This limits the generalisability of our findings to geographical settings outside the study areas. The study was cross-sectional, and therefore only able to suggest associations rather than causal relationships. The data also

For peer review only - http://bmjopen.bmj.com/site/about/guidelines.xhtml

did not include key predictors of caesarean section such as breech presentation, and whether the previous delivery was by caesarean, which limits the completeness of our analysis of determinants. In addition, levels of serious complications in pregnancy and delivery varied considerably between locations and were often higher than expected, suggesting potential over-reporting and limited reliability as an indicator of complications. Finally, some variables had small denominators, and others had high levels of missing data (for example, maternal age in rural India).

Conclusions

Our study found that delivering in a private health facility was associated with an increased odds of caesarean section in three of four South Asian locations, and that the associations persisted after adjustments for maternal, pregnancy and delivery characteristics, and year of delivery. We also found significant interactive associations between maternal education and caesarean delivery in Bangladesh (both private and public facilities) and urban India (private facilities only). These results lend support to the hypothesis that increased caesarean section rates in these three South Asian countries may in part be driven by the private sector, but also suggest that, in some settings such as Bangladesh and urban India, demand from more educated mothers may play a part. These findings call for greater, local understanding of the role of private providers in maternity care, together with careful examination of the consequences of increased caesarean sections in the private sector for maternal and neonatal health outcomes.

Competing interests

None to declare

Contributors

MN and AP had the original idea for the study. MN designed the data analysis strategy in discussion with AP. MN, GA, AS, CS and NS prepared the data. MN performed the analysis and prepared the tables. NN, PT, NSM, DO, KA, DM and AC are responsible for the trials. AP wrote the first draft of the paper and all authors read and commented on the draft. All authors read and approved the final manuscript.

For peer review only - http://bmjopen.bmj.com/site/about/guidelines.xhtml

Funding

MN and NS are funded through a Wellcome Trust Strategic Award (085417MA/Z/08/Z). In Bangladesh, the Perinatal Care Project trial (covering KA and AK) was registered with trial number ISRCTN54792066 and has received funding from the Big Lottery Fund and the Wellcome Trust. In Eastern India, the Ekjut trial (covering NN and PT) was registered with trial number ISRCTN21817853 and funded by the Health Foundation. The Dhanusha (Nepal) trial (covering DSM, NS, BPS, and CS) was registered with trial number ISRCTN87820538 and has received funding from: the UBS Optimus Foundation; USAID; DfID and the Wellcome Trust. The City Initiative for Newborn Health trial led by SNEHA (Mumbai, India) was registered with trial number ISRCTN96256793 and has received funding from the ICICI Foundation for Inclusive Growth and the Wellcome Trust. GA, NSM, and DO are currently funded by The Wellcome Trust (091561/Z/10/Z). The funders played no part in the development of the article or the decision to submit it for publication.

Provenance and peer review

Not commissioned; externally peer reviewed.

REFERENCES:

1. World Health Organisation and UNICEF. Countdown 2015: Building a future for women and Children, the 2012 Report. http://countdown2015mnch.org/documents/2012Report/2012-Complete.pdf [Accessed 9th November 2013]
2. Campbell OM, Graham WJ. Strategies for reducing maternal mortality: getting on with what works. *The Lancet* 2006; 368: 1284-1299.
3. Ronsmans C, De Brouwere V, Dubourg D, *et al*.. Measuring the need for life-saving obstetric surgery in developing countries. *BJOG* 2004; 111: 1027-30.
4. World Health Organization. Appropriate technology for birth. *The Lancet* 1985; 2: 436-7.
5. Betrán AP, Merialdi M, Lauer JA, *et al*. Rates of caesarean section: analysis of global, regional and national estimates. *Paediatric and Perinatal Epidemiology* 2007; 21: 98-113.
6. Cavallaro FL, Cresswell JA, França GVA, *et al*. Trends in Caesarean delivery by country and wealth quintile: cross-sectional surveys in southern Asia and sub-Saharan Africa. *WHO Bulletin* 2013; 91: 897-972.
7. Souza JP, Gülmezoglu A, Lumbiganon P, *et al*. WHO Global Survey on Maternal and Perinatal Health Research Group. Caesarean section without medical indications is associated with an increased risk of adverse short-term maternal outcomes: the 2004–2008 WHO Global Survey on Maternal and Perinatal Health. *BMC Medicine* 2010; 8:71.
8. Kyu HH, Shannon HS, Georgiades K, Boyle MH. Caesarian delivery and neonatal mortality rates in

For peer review only - http://bmjopen.bmj.com/site/about/guidelines.xhtml

46 low- and middle-income countries: a propensity score matching and meta-analysis of demographic health survey data. *International Journal of Epidemiology* 2013; 1-11.

9. Gibbons L, Belizán JM, Lauer JA, *et al*. The global numbers and costs of additionally needed and unnecessary caesarean sections performed per year: overuse as a barrier to universal coverage. World Health Report (2010), Background paper. Geneva: World Health Organization.
10. Mitra SN, Nawab Ali M, Islam S, *et al*. Bangladesh Demographic and Health Survey, 1993-1994. Calverton, Maryland: National Institute of Population Research and Training (NIPORT), Mitra and Associates, and Macro International Inc, 1994.
11. National Institute of Population Research and Training (NIPORT), Mitra and Associates, and ICF International. Bangladesh Demographic and Health Survey 2011. Dhaka, Bangladesh and Calverton, Maryland, USA: NIPORT, Mitra and Associates, and ICF International, 2013.
12. International Institute for Population Sciences (IIPS) and Macro International. National Family Health Survey (NFHS 1), 1992–93. India, Mumbai: IIPS, 1995.
13. International Institute for Population Sciences (IIPS), Macro International. National Family Health Survey (NFHS 3), 2005–06; India: Volume I. Mumbai: IIPS, 2007.
14. Pradhan A, Aryal RH, Regmi G, *et al*. Nepal Family Health Survey 1996. Kathmandu, Nepal and Calverton, Maryland: Ministry of Health [Nepal], New ERA, and Macro International Inc, 1997.
15. Ministry of Health and Population (MOHP) [Nepal], New ERA, and ICF International Inc. Nepal Demographic and Health Survey 2011. Kathmandu, Nepal: Ministry of Health and Population, New ERA, and ICF International, Calverton, Maryland, 2012.
16. Ajeet S, Nandkishore K. The boom in unnecessary caesarean surgeries is jeopardizing women's health. *Health Care Women International* 2013; 34: 513-521.
17. Sreevidya S, Sathiyasekaran BW. High caesarean rates in Madras (India): a population-based cross-sectional study. *BJOG* 2003; 110: 106-111.
18. Koblinksi M, Anwar I, Mridha MK, *et al*. Reducing maternal mortality and improving maternal health: Bangladesh and MDG 5. *J Health Popul Nutr* 2008; 26: 280-294.
19. Leone T, Padmadas SS, Matthews Z. Community factors influencing rising caesarean section rates in developing countries: an analysis of six countries. *Soc Sci Med* 2008; 67: 1236-46.
20. Mazzoni A, Althabe A, Liu NH, *et al*. Women's preference for caesarean section: a systematic review and meta-analysis of observational studies. *BJOG* 2011; 118: 391-399.
21. Tripathy P, Nair N, Barnett S, et al. Effect of a participatory intervention with women's groups on birth outcomes and maternal depression in Jharkhand and Orissa, India: A cluster-randomised controlled trial. *Lancet* 2010; 375: 1182–1192.
22. Azad K, Barnett S, Banerjee B, *et al*. Effect of scaling up women's groups on birth outcomes in three rural districts in Bangladesh: a cluster-randomised controlled trial. *Lancet* 2010; 375: 1193–1202.

For peer review only - http://bmjopen.bmj.com/site/about/guidelines.xhtml

23. Fottrell E, Azad K, Kuddus A, *et al*. The effect of increased coverage of participatory women's groups on neonatal mortality in Bangladesh: a cluster-randomised trial. *JAMA Pediatrics* 2013; 167: 816-825.

24. Shah More N, Bapat U, Das S, *et al*. Cluster-randomised controlled trial of community mobilisation in Mumbai slums to improve care during pregnancy, delivery, postpartum and for the newborn. *Trials* 2008; 9:7.

25. Shrestha BP, Bhandari B, Manandhar DS, *et al*. Community interventions to reduce child mortality in Dhanusha, Nepal: study protocol for a cluster randomized controlled trial. *Trials* 2011; 12: 136.

26. Prost A, Colbourn T, Seward N, *et al*. Women's groups practising participatory learning and action to improve maternal and newborn health in low-resource settings: a systematic review and meta-analysis. *Lancet* 2013; 381: 1736–46.

27. Schmidt J-0, Ensor T, Hossain A, Khan S. Vouchers as demand side financing instruments for health care: A review of the Bangladesh maternal voucher scheme. *Health Policy* 2010; 96: 98–107.

28. Ahmed S, Khan MM. A maternal health voucher scheme: what have we learned from the demand-side financing scheme in Bangladesh? *Health Policy & Planning* 2011; 6:25–32.

29. Lim SS, Dandona L, Hoisington JA, *et al*. India's Janani Suraksha Yojana, a conditional cash transfer programme to increase births in health facilities: an impact evaluation**.** *Lancet* 2010; 375: 209-23.

30. Shah More N, Alcock G, Bapat U, *et al*. Tracing pathways from antenatal to delivery care for women in Mumbai, India: cross-sectional study of maternity in low-income areas. International Health 2009; 1: 71–77.

31. Government of India, Ministry of Health & Family Welfare. Janani Suraksha Yojana: Guidelines for Implementation, 2006. http://www.mohfw.nic.in/NRHM/./guidelines/JSY_guidelines_09_06.pdf

32. Witter S, Khadka S, Nath H, Tiwari S. The national free delivery policy in Nepal: early evidence of its effects on health facilities. *Health Policy and Planning* 2011; 26: ii84–ii91.

33. Holgado-Tello FP,Chacón–Moscoso S, Barbero-Garcia I, Vila-Abad E. Polychoric versus Pearson correlations in exploratory and confirmatory factor analysis of ordinal variables. *Quality & Quantity* 2010; 44 (1): 153-166.

34. Rabe-Hesketh S, Skrondal A, Pickles A. Maximum likelihood estimation of limited and discrete dependent variable models with nested random effects. *Journal of Econometrics* 2005; 128: 301-323.

35. StataCorp. 2013. *Stata Statistical Software: Release 13*. College Station, TX: StataCorp LP.

36. Potter J, Berquó E, Perpétuo I, *et al*. Unwanted Caesarean Sections among Public and Private

For peer review only - http://bmjopen.bmj.com/site/about/guidelines.xhtml

Patients in Brazil: Prospective Study. *BMJ* 2001; 323: 1155-8.

37. Kamal SMM. Preference for institutional delivery and caesarean sections in Bangladesh. *J Health Popul Nutr* 2013; 31: 96-109.
38. Randive B, Diwan V, De Costa A. India's Conditional Cash Transfer Programme (the JSY) to Promote Institutional Birth: Is There an Association between Institutional Birth Proportion and Maternal Mortality? *PLoS ONE* 2013; 8(6): e67452. doi:10.1371/journal.pone.0067452
39. Chatterjee S, Laxminarayan R. Costs of surgical procedures in Indian hospitals. *BMJ Open* 2013; 3: e002844.
40. Parkhurst JO, Rahman SA. Life saving or money wasting? Perceptions of caesarean sections among users of services in Rural Bangladesh. *Health Policy* 2007; 80: 392-401.
41. Khan R, Blum LS, Sultana M, et al. An Examination of Women Experiencing Obstetric Complications Requiring Emergency Care: Perceptions and Sociocultural Consequences of Caesarean Sections in Bangladesh. *J Health Popul Nutr 2012*; 30: 159-171.
42. Pomeroy A, Koblinksy M, Alva S. Private Delivery Care in Developing Countries: Trends and Determinants. DHS Working document 2010; No.76. http://dhsprogram.com/pubs/pdf/WP76/WP76.pdf [Accessed 3rd March 2014].
43. Sufang G, Padmadas SS, Fengmin Z et al. Delivery settings and caesarean section rates in China. *Bull WHO* 2007; 85: 733-820.
44. Lo JC. Patients' attitudes vs. physicians' determination: implications for cesarean sections. *Soc Sci Med* 2003; 57: 91-6.
45. McCourt C, Weaver J, Statham H, *et al.* Elective cesarean section and decision making: a critical review of the literature. *Birth* 2007; 34: 65-79.
46. Ghetti C, Chan BKS, Guise J-M. Physicians' responses to patient-requested caesarean delivery. *Birth* 2004; 31: 280-4.

For peer review only - http://bmjopen.bmj.com/site/about/guidelines.xhtml

Table 1: Characteristics of studies and populations

Study (Country)	Bangladesh (rural)	India (rural)	Nepal (rural)	India (urban)
**Location**	Three districts: Bogra, Maulvibazaar and Faridpur	Three districts of Jharkhand and Odisha: Keonjhar, West Singhbhum and Saraikela	Dhanusha district (Terai)	Mumbai slums
**Period**	2005 – 2011	2005-2008	2008-2011	2006-2009
**Estimated population**	532,900	114,000	240,000	283,000
**Cluster characteristics**	Villages making up a union	8-10 villages with residents classified as Scheduled Tribe or Other Backward Class	Village Development Committee	Slum areas in six municipal wards of Mumbai
**Method of cluster identification**	Purposive sampling of three districts and clusters within districts	Purposive sampling of three districts and clusters within districts	Random sampling of 60 clusters from a list of 79 suitable clusters in one district	92 clusters in six municipal wards identified using municipal documents, surveys, discussions with key informants, and site visits. Random selection of 48 clusters for randomized allocation.
**Clusters, n**	9	18	30	24
**Cluster and individual follow-up**	All clusters followed up<br>Interviews completed after 82% of identified births in control areas in Phase 1, and 99% of births in Phase 2	All clusters followed up<br>Interviews completed after 98% of identified births	All clusters followed up	All clusters followed up<br>Interviews completed after 83% of identified births
**Maternal mortality ratio**	254.3	668.1	Unknown	206.2
**Health facilities available in control areas**	Public facilities: District Hospitals; Maternal and Child Welfare Centres; Upazilla Health Complexes. Private facilities: small-to-medium-size clinics; BRAC (NGO) facilities where deliveries do not take place; larger private hospitals with and without Comprehensive emergency obstetric care (CEmOC) facilities.	Public facilities: District Hospitals; Primary Health Centres (PHCs) in which deliveries can notionally take place but that are not usually equipped for CEmOC; Community Health Centres (CHCs) acting as referral centres for PHCs, covering a population of around 80,000 with EmOC facilities; district hospitals. Private and charitable facilities: medium-sized missionary hospitals with EmOC facilities.	Public facilities: three Primary Health Care Centres, three Health Posts and 24 Sub-Health Posts, none of which are equipped for CEmOC. These health facilities refer to the public Zonal Tertiary Hospital and various private providers in the district headquarters and nearby medical college, which have facilities for caesarean sections.	Public facilities: Municipal tertiary hospitals, general hospitals, and maternity homes. Private facilities: specialty hospitals, general hospitals, and maternity homes.

For peer review only

Table 2: Characteristics of institutional births and caesarean deliveries by location

	Births	Institutional births						Caesarean section births			
		All		Public facility		Private/charitable facility		Public facility		Private/charitable facility	
	(n)	(n)	(% all births)	(n)	(% institutional births)	(n)	(% institutional births)	(n)	(% deliveries in public facilities)	(n)	(% deliveries in private facilities)
**Bangladesh (rural)**	21,560	4,592	21%	2,053	45%	2,539	55%	589	29%	1,852	73%
**India (rural)**	8,541	1,816	21%	421	23%	1,395	77%	64	15%	66	5%
**India (urban)**	10,236	9,259	90%	5,489	59%	3,770	41%	811	15%	697	18%
**Nepal (rural)**	4,931	1,586	32%	1,424	90%	162	10%	201	14%	48	30%

* Nepal numbers weighted using women's probability of selection within each cluster, scaled to total number of institutional births. (Unweighted Nepal totals: 4,990 births and 1,586 institutional births)

For peer review only - http://bmjopen.bmj.com/site/about/guidelines.xhtml

Table 3: Mutually adjusted associations of type of facility of delivery and other selected determinants with caesarean section delivery, by location

	Bangladesh (rural) ORs (95% CIs)		India (rural) ORs (95% CIs)		India (urban) ORs (95% CIs)		Nepal (rural)* ORs (95% CIs)	
	Crude	Adjusted	Crude	Adjusted	Crude	Adjusted	Crude	Adjusted
**Health facility characteristics**								
Public health facility (ref)								
Private health facility	6.82 (5.96,7.81)	5.91 (5.15,6.78)	0.39 (0.25,0.61)	0.46 (0.29,0.73)	1.36 (1.21,1.52)	1.22 (1.09,1.38)	2.42 (1.48,3.94)	2.37 (1.62,3.44)
**Pregnancy and delivery characteristics**								
4+ antenatal care visits (fewer or none = ref)**		1.46 (1.26,1.69)		1.49 (0.96,2.32)		1.06 (0.93,1.21)		1.92 (1.43,2.58)
Birth order								
1 (ref)								
2		0.95 (0.80,1.13)		1.25 (0.80,1.95)		0.65 (0.56,0.75)		1.41 (1.02,1.94)
3		1.19 (0.94,1.52)		0.36 (0.15,0.87)		0.60 (0.50,0.71)		0.58 (0.37,0.92)
4+		1.17 (0.90,1.53)		0.36 (0.15,0.85)		0.39 (0.32,0.48)		1.00 (0.54,1.84)
Serious complications in pregnancy/delivery*** (no complications=ref)		0.87 (0.76,1.00)		1.77 (1.17,2.67)		1.71 (1.39,2.11)		4.87 (2.51,9.47)
Multiple birth (ref=single)		0.93 (0.65,1.32)		1.57 (0.52,4.75)		3.01 (2.14,4.23)		3.42 (1.77,6.61)
**Maternal characteristics**								
Age (years)								
15-24 (ref)								
25-34		1.12 (0.94,1.34)		1.81 (1.14,2.86)		1.45 (1.27,1.66)		1.52 (0.63,1.94)
35+		1.10 (0.77,1.58)		2.11 (0.63,7.07)		1.79 (1.27,2.53)		1.55 (0.55,1.47)

For peer review only - http://bmjopen.bmj.com/site/about/guidelines.xhtml

	Bangladesh (rural) ORs (95% CIs)		India (rural) ORs (95% CIs)		India (urban) ORs (95% CIs)		Nepal (rural)* ORs (95% CIs)	
	Crude	Adjusted	Crude	Adjusted	Crude	Adjusted	Crude	Adjusted
Maternal education****								
No formal education (ref)								
Primary education		1.07		0.98		1.13		0.90
		(0.83,1.38)		(0.39,2.47)		(0.85,1.49)		(0.59,1.39)
Secondary education		1.44		1.11		1.22		1.37
		(1.13,1.84)		(0.68,1.80)		(1.04,1.42)		(0.97,1.94)
Bachelor's degree or higher		2.44		1.20		1.62		-
		(1.52,3.92)		(0.43,3.31)		(1.30,2.02)		-
Household wealth quintile								
1st (poorest, ref)								
2nd		1.31		1.61		1.14		1.11
		(1.06,1.63)		(0.69,3.75)		(0.77,1.68)		(0.63,1.94)
3rd		1.41		1.36		1.13		0.90
		(1.10,1.82)		(0.58,3.21)		(0.95,1.33)		(0.55,1.47)
4th (wealthiest)		1.36		2.16		1.50		0.99
		(1.09,1.70)		(0.87,5.33)		(1.27,1.78)		(0.52,1.90)
Year of delivery								
2004-2006 (ref: BD, IN)								-
2007-2009 (ref: NP rural)		0.90		0.90		1.14		-
		(0.74,1.10)		(0.61,1.33)		(1.09,1.38)		-
2010-2012		1.15		-		-		1.29
		(0.94,1.41)		-		-		(0.91,1.82)
N	4,592	4,592	1,816	1,816	9,259	9,259	1,586	1,586

* Nepal numbers weighted using women's probability of selection within each cluster, scaled to total number of institutional births.

** At least one visit with skilled provider

***Includes: symptoms of eclampsia (fits, seizures, convulsions, or unconsciousness during pregnancy or delivery); reduced or no fetal movement; labour lasting more than 24 hours.

****For the Nepal data, two respondents with bachelor's degrees (2 respondent total, 1 delivering in institution) were combined with respondents with secondary education.

All analyses additionally adjusted for survey design using fixed effect of stratum and random effect of cluster.

For peer review only

For peer review only - http://bmjopen.bmj.com/site/about/guidelines.xhtml

Table 4: Interactive associations between type of delivery facility, maternal education, and caesarean delivery, by location

	Bangladesh (rural)		India (rural)		India (urban)		Nepal (rural)*	
	(n)	AOR, 95% CI	(n)	AOR, 95% CI	(n)	AOR, 95% CI	(unweighted n)	AOR, 95% CI
Public facility - no maternal education (ref)	202	-	109	-	1,396	-	822	-
Public facility - primary education	448	1.07 (0.76,1.50)	17	0.92 (0.18,4.77)	357	0.88 (0.61,1.27)	196	1.04 (0.65,1.65)
Public facility - secondary education	202	1.63 (1.18,2.24)	604	1.09 (0.52,2.26)	920	1.1 (0.91,1.33)	413	1.45 (1.04,2.02)
Public facility - bachelor's degree	448	4.58 (2.24,9.38)	100	1.55 (0.41,5.89)	180	1.06 (0.76,1.47)	-	- -
Private facility - no education	332	7.24 (4.82,10.87)	109	0.46 (0.21,1.01)	1396	0.91 (0.70,1.19)	100	2.83 (1.73,4.61)
Private facility - primary education	578	7.58 (5.36,10.71)	17	0.47 (0.14,1.58)	357	1.52 (0.99,2.33)	16	0.86 (0.14,5.21)
Private facility - secondary education	1,102	9.03 (6.57,12.41)	274	0.52 (0.24,1.12)	3,382	1.3 (1.06,1.60)	39	3.01 (1.71,5.30)
Private facility - bachelor's degree	41	11.24 (6.37,19.85)	21	0.4 (0.08,2.00)	354	2.1 (1.61,2.75)	-	- -
N	3,353		1,251		8,342		1586	
p-value**	0.021		0.792		0.001		0.394	

All results adjusted for number of antenatal care visits, parity, medical indication for caesarean, multiple birth, maternal age (10-yr group), household assets, stratum, and cluster (random effect).
* Nepal numbers weighted using women's probability of selection within each cluster, scaled to total number of institutional births.

** p-value from -2 log-likelihood test comparing nested models with and without interaction terms

For peer review only - http://bmjopen.bmj.com/site/about/guidelines.xhtml

Prevalence and determinants of caesarean section in private and public health facilities in underserved South Asian communities: cross-sectional analysis of data from Bangladesh, India, and Nepal

Melissa Neuman[1], Glyn Alcock[1], Kishwar Azad[4], Abdul Kuddus[4], David Osrin[1], Neena Shah More[3], Nirmala Nair[5], Prasanta Tripathy[5], Catherine Sikorski[1], Naomi Saville[1], Aman Sen[2], Tim Colbourn[1], Tanja AJ Houweling[6], Nadine Seward[1], Dharma S Manandhar[2], Bhim P Shrestha[2], Anthony Costello[1] and Audrey Prost[1]

Author affiliations:

[1] Institute for Global Health, University College London, 30 Guilford Street, London WC1N 1EH, UK

[2] Mother and Infant Research Activities (MIRA), YB Bhavan, Thapathali, GPO Box 921, Kathmandu, Nepal

[3] Society for Nutrition, Education and Health Action (SNEHA), Urban Health Centre, Shahunagar, Dharavi, Mumbai 400017, Maharashtra, India

[4] Perinatal Care Project, Diabetic Association of Bangladesh, 122 Kazi Nazrul Islam Avenue, Dhaka 1000, Bangladesh

[5] Ekjut, Plot 556B, Potka, Chakradharpur, West Singhbhum, Jharkhand, India

[6] Department of Public Health, Erasmus MC University Medical Center Rotterdam, P.O. Box 2040, 3000 CA Rotterdam, The Netherlands

Proposed running head: Caesarean sections in private vs. public health facilities in South Asia

Word count: 4393

Figures and tables: 0 figures and 4 tables

For peer review only

ABSTRACT (Max: 300 words; Current: ~~295~~300)

Objectives: To describe the prevalence and determinants of births by caesarean section in private and public health facilities in underserved communities in South Asia.

Design: Cross-sectional study.

Setting: 81 community-based geographical clusters in four locations in Bangladesh, India and Nepal (three rural, one urban).

Participants: 45,327 births occurring in the study areas between 2005 and 2012.

Outcome measures: proportion of caesarean section delivery by location and type of facility; determinants of caesarean section delivery by location.

Results: Institutional delivery rates varied widely between settings, from 21% in rural India to 90% in urban India. The proportion of private and charitable facility births delivered by caesarean section was 73% in Bangladesh, 30% in rural Nepal, 18% in urban India, and 5% in rural India. The odds of caesarean section were greater in private and charitable health facilities than in public facilities in three of four study locations, even when adjusted for pregnancy and delivery characteristics, maternal characteristics, and year of delivery (Bangladesh AOR: 5.91, 95% CI: 5.15-6.78; Nepal AOR: 2.37, 95% CI: 1.62-3.44; urban India AOR: 1.22, 95% CI: 1.09-1.38). We found significant interactive associations between maternal education and private facility delivery in two of four locations ~~(p=0.025 in rural Bangladesh and p<0.001 in urban India)~~, with highly educated women particularly likely to deliver by caesarean in private facilities in both ~~locations.~~urban India (AOR 2.10; 95% CI: 1.61, 2.75) and rural Bangladesh (AOR 11.09, 95% CI: 6.28, 19.57).

Conclusions:

Our results lend support to the hypothesis that increased caesarean section rates in these South Asian countries may be driven in part by the private sector. They also suggest that preferences for caesarean delivery may be higher among highly educated women, and that individual- and provider-level factors interact in driving caesarean rates higher. Rates of caesarean section in the private sector, and their maternal and neonatal health outcomes, require close monitoring.

For peer review only

Article summary

Article focus

- Caesarean sections are on the rise in South Asia. Research suggests that private health facilities may be contributing to this by conducting caesareans over and above clinical need.
- Literature from other settings indicates that increases in caesarean sections are shaped by both supply and demand pressures: providers often have financial incentives to intervene surgically, and women of higher socio-economic status are also more likely to want caesareans.
- This study examined the prevalence and determinants of caesarean sections in private and charitable facilities compared with public health facilities for over 45,000 births in underserved communities of rural Nepal, rural Bangladesh, rural and urban India.

Key messages

- Caesarean sections were more common in private than in public health facilities in three of four study locations (urban India, rural Nepal, and rural Bangladesh).
- We found significant interactive associations between maternal education and private facility ~~caesarean~~ delivery on caesarean in Bangladesh (both private and public facilities) and urban India (private facilities only).
- Future research should monitor caesarean section rates in the private sector in South Asia and subsequent maternal and neonatal health outcomes

Strengths and limitations

- This study had a large sample size and focused on underserved communities, which are a priority for public health interventions in South Asia.
- Our data were not nationally representative, which limits the generalisability of our findings. Other limitations included small denominators and missing data for some variables. We did not have data on some known predictors of caesarean section, which would have enhanced the completeness of our determinants analysis.

For peer review only - http://bmjopen.bmj.com/site/about/guidelines.xhtml

INTRODUCTION

Access to comprehensive emergency obstetric care, including caesarean section, is key to preventing the estimated 287,000 maternal and 2.9 million neonatal deaths that occur worldwide every year.[1 2] Although debate continues about how to quantify the need for life-saving obstetric surgery, a 1985 World Health Organization (WHO) report suggested that the optimal population range for caesarean section rates is between 5% and 15%, and this endures as a reference.[3 4] Caesarean section rates are increasing worldwide, albeit unequally: a recent analysis of Demographic and Health Survey (DHS) data in 26 South Asian and sub-Saharan African countries found that rates were highest among the 'urban rich' in all countries, and lowest among the 'rural poor' in 18.[5] In all countries, fewer than 5% of mothers in the poorest wealth quintile delivered by caesarean.[6]

Caesarean sections conducted without clinical need can have adverse consequences for mothers and children. A 2008 WHO survey of 373 facilities across 24 countries found that unnecessary caesareans were associated with an increased risk of maternal mortality and serious outcomes for mothers and newborn infants, compared with spontaneous vaginal delivery.[7] Recent ecological analyses also highlighted strong associations between caesarean delivery and increased neonatal mortality in countries with low and medium caesarean section rates.[8] Unnecessary caesareans lead to considerable costs for families and health systems: an estimated 6.2 million unnecessary procedures were performed in 2008, costing approximately US$ 2.32 billion. [9]

Several South Asian countries have recorded substantial increases in caesarean section rates over the past decade. In Bangladesh, rates rose from 2% (2000) to 17% (2011); in India, from 3% (1992) to 11 % (2006); and in Nepal, from 1% (2000) to 5% (2011).[10-5] Several studies in these countries have raised concerns about high caesarean rates in private facilities, and a recent DHS analysis speculated that national increases in caesarean section rates in South Asian countries could be driven in part by higher rates among deliveries in private sector facilities[.6 16-18] Literature from other settings indicates that increases in caesarean sections are shaped by both supply and demand pressures: providers often have financial incentives to intervene surgically, and women of higher socio-economic status are also more likely to desire caesareans.[19 20]

There are few large, recent community-based studies from South Asia quantifying differences in caesarean section rates between public and private facilities. Such studies are necessary in order to examine whether increases in caesareans in these settings are indeed likely to be driven by the private sector, demand from wealthier and more educated mothers, or a combination of the two. We conducted a cross-sectional analysis of data from Bangladesh, India, and Nepal to explore the prevalence and determinants of caesarean section delivery by type of facility and location, and to examine the

For peer review only - http://bmjopen.bmj.com/site/about/guidelines.xhtml

interactions between maternal education and caesarean section delivery in and place of delivery private and public facilitieson prevalence of caesarean section.

METHODS

Study populations

We used data collected through vital events surveillance systems established during four cluster-randomised controlled trials conducted between 2005 and 2011. The trials were done with communities that can be considered socio-economically disadvantaged: in Bangladesh and Nepal, they took place in four rural, underserved districts (Bogra, Maulvibazaar, and Faridpur in Bangladesh, and the Terai district of Dhanusha, Nepal); in rural India, most participants were from Scheduled Tribes in two states of eastern India (Jharkhand and Odisha); in urban India, data came from slum communities in Mumbai. **Table 1** describes the characteristics of each study and its population, including the background maternal mortality ratio and types of facilities present in the study areas. The original trials were designed to evaluate the impact of participatory women's groups on maternal and neonatal health outcomes.[21-25] We used data from the control areas of these trials only, because the women's group intervention led to changes in mortality and practices in several locations.[26]

Health system contexts

All three study countries have experienced substantial increases in institutional delivery rates over the last two decades: births in health facilities increased from 4% (1993) to 29% (2011) in Bangladesh; from 26% (1992-3) to 47% (2007-8) in India; and from 8% (1996) to 35% (2011) in Nepal.[10-15] All three countries have implemented incentive schemes to promote institutional delivery, though these have varying coverage. In 2010, Bangladesh's pilot maternity voucher scheme reached an estimated 10.4 million people across 31 sub-districts, around 7% of the country's population.[27 28] Mothers receive a cash incentive for antenatal care and delivery in a public or private facility, or at home with a skilled birth attendant. Both government and private facility staff also receive cash incentives, including 3000 Bangladeshi Taka (US $38.5) for a caesarean section and 300 Taka for a normal delivery.[28] This maternity incentive scheme was operational in two of the three districts (Faridpur and Maulvibazaar) covered by our study. All three districts had public facilities including District Hospitals, Maternal and Child Welfare Centres, and Upazilla Health Complexes. Private facilities included a number of small-to-medium-size clinics, BRAC (NGO) facilities, and larger private hospitals.

In India, the *Janani Suraksha Yojana* (JSY) maternity incentive scheme entitles women in rural areas of high focus states, including those in this study, to 1400 Indian Rupees ($22.4) after delivering in a government or accredited private health facility. Local community health volunteers called Accredited Social Health Activists (ASHAs) also receive 600 Indian Rupees ($9.6) for identifying pregnant women and

For peer review only - http://bmjopen.bmj.com/site/about/guidelines.xhtml

For peer review only

helping them get to a health facility.[29] Although the rural Indian data included in this study were collected between 2005 and 2008, the JSY was only operational in the study areas from 2008 onwards and its impact is unlikely to be reflected here. The Indian urban area included in our study spanned informal settlements (slums) with a wealth of public and private providers.[30] Mothers with Below Poverty Line cards in such areas are eligible for JSY and can receive Rs 500 towards the costs of delivering in a health facility.[31] Nepal began a safe delivery incentive scheme in 2005 and free deliveries have been available in government facilities since 2009 through the *Aama Surakshya* programme.[32] Dhanusha district, from which data for this study come, has one zonal tertiary hospital and a private medical college hospital equipped for c-sections, and a variety of small public and private health facilities without comprehensive obstetric care.

Data collection

All data were collected using surveillance systems to monitor births and deaths prospectively.[21-25] In all study locations, a female community-based key informant reported births and deaths in her area, which covered a population ranging from 250 to 350. A trained interviewer then verified these reports and paid the informant an incentive for each correct identification. In Bangladesh, rural and urban India, the interviewer administered a structured questionnaire to all eligible mothers around six weeks after delivery; in Nepal, all births in the study area were registered, and interviews were conducted on all births in small clusters and on a random sample of 10 births per month in the larger clusters. In each study location, mothers were interviewed using a questionnaire to collect information about events in the antenatal, delivery and postnatal periods. In each trial and in this subsequent study, participants were women of reproductive age (15-49) who delivered in the study area during the data collection period, and who consented to be interviewed six weeks after delivery, and their infants.

Study sample

The initial sample for this analysis included 46,393 births in Mumbai, rural India, rural Bangladesh, or rural Nepal, of whom 17,565 (38%) were delivered in health care facilities. We excluded the 2348 births occurring outside Mumbai, because we collected limited information on delivery location for these. Additionally, 223 deliveries had missing data on form of delivery (caesarean or vaginal), 602 had missing or implausible values for mother's age, and 98 were missing information on other maternal characteristics (educational attainment, household assets, number of prior pregnancies, number of antenatal visits, severe problems in pregnancy or delivery). 143 births had missing or incomplete information on place of delivery. The final analytic sample included 45,327 births (97.7% of the initial sample).

For peer review only - http://bmjopen.bmj.com/site/about/guidelines.xhtml

For peer review only

Measures used

The primary outcome in these analyses was caesarean section delivery, identified by self-report from the mother or another household member around six weeks after giving birth. The main covariate of interest was the type of delivery facility, coded as public (e.g. district hospitals), private (individual private clinics or hospitals), or NGO/charitable (e.g. Christian missionary hospitals in rural India; BRAC clinics in Bangladesh). We grouped private and NGO/charitable facilities together due to the small number of births in NGO/charitable facilities (6% of institutional deliveries in Bangladesh, 7% in rural India, and 0 in urban India and rural Nepal; data on request). Additional covariates in the models included measures of the characteristics of the pregnancy and delivery, maternal socio-demographic characteristics, and the location in which the delivery occurred. We created an indicator variable to identify women experiencing serious problems during pregnancy or delivery, with women reporting symptoms of pre-eclampsia (blurred vision or swelling of face and hands), symptoms of eclampsia (fits or seizures during pregnancy or delivery), hæmorrhage during delivery, or labour lasting more than 24 hours considered to have serious complications. Other characteristics of the pregnancy and delivery considered in the analysis included full utilisation of antenatal care, number of prior pregnancies, and multiple pregnancy. Full utilization of antenatal care was defined as four or more visits to antenatal care, with at least one visit to a skilled provider. Number of pregnancies was entered as an ordered categorical variable, with categories of one (first), two, three, or four or more pregnancies.

Maternal socio-demographic characteristics included in the models were: mother's age at delivery, her educational attainment, and household assets. Mother's age was entered into the model as a categorical measure in 10-year groups. Educational attainment was entered as a categorical variable using the following categories: no formal education, primary education, secondary education, or bachelor's degree or higher. To develop an asset index, we used polychoric factor analysis on data on common assets and amenities found in the mother's household, and grouped the resulting factor scores into quartiles.[33] Assets and amenities included electricity, radio or cassette player, electric fan (Bangladesh and India only), television, refrigerator (Bangladesh and India only), telephone (Bangladesh and Nepal only), generator (India only), and bicycle. All models were additionally adjusted for location (Bangladesh, India, Nepal), and year of interview in three-year groups. The data were collected in a stratified, cluster-sampled survey, and we accounted for survey design in the analysis using a fixed effect for stratum and a random effect for cluster.

Statistical analysis

We used frequencies to describe caesarean section rates by delivery location at each site. We used the Generalized Linear Latent And Mixed Models (GLLAMM) procedure in Stata 13.1, with adaptive

For peer review only - http://bmjopen.bmj.com/site/about/guidelines.xhtml

quadrature for binary outcomes, to estimate the crude association between type of delivery facility and caesarean section.[34 35] We identified other maternal, pregnancy, and delivery characteristics potentially associated with caesarean section using existing literature, especially studies resulting from the WHO multi-country surveys, and entered these in adjusted models to explore how they modified the association between type of delivery facility and caesarean section, and their individual association with caesarean section. Some South Asian and Latin American studies have detected a strong association between maternal education and caesarean delivery. [7 36 37] We fitted models including indicator variables for each group of mothers by education and type of delivery facility to explore differences in the strength of association between caesarean delivery and private facility by mother's education. To account for the sampling procedure used in rural Nepal, models were adjusted using pweights (probability of selection within cluster); these weights were rescaled to reflect the total number of institutional deliveries.

Ethical approval

The trials that provided data for this study received ethical approval from the following committees: the ethics committee of the Diabetic Association of Bangladesh (Perinatal Care Project, Bangladesh Diabetes Somity, BADAS); an independent ethics committee in Jamshedpur, Jharkhand, India; the Nepal Health Research Council; the Municipal Corporation of Greater Mumbai and the Independent Ethics Committee for Research on Human Subjects (Mumbai, India); the ethics committee of the Institute of Child Health, University College London.

RESULTS

Table 2 describes the characteristics of institutional births and caesarean deliveries by location. We analysed data for 45,327 births: 21,560 in rural Bangladesh, 8,541 in rural India, 10,236 in urban India, and 4,931 in rural Nepal. The proportion of women delivering in health facilities varied widely between locations, from 90% in urban India to 21% in rural India There were also large variations in the proportion of women delivering in private/charitable facilities rather than public facilities, with the highest (77%) in rural India and the lowest (10%) in rural Nepal. The proportion of women giving birth by caesarean section in private rather than public facilities also varied widely between settings. In Bangladesh, only 21% of women delivered in a health facility, around half of them in the private/charitable sector, but 73% of private facility births were by caesarean section. In rural Nepal, 30% of the 162 private facility births were by caesarean section. In informal settlements of Mumbai, 15% of public facility deliveries were caesareans, compared with 18% of private facility deliveries. In rural India, caesarean sections were more commonly performed in public health facilities than private or charitable facilities (15% vs. 5%). **Supplemental table 1** describes the characteristics of institutional deliveries (caesarean or non-caesarean) according to type of delivery facility (private or public), maternal

For peer review only - http://bmjopen.bmj.com/site/about/guidelines.xhtml

For peer review only

socio-economic and socio-demographic characteristics, pregnancy and delivery characteristics, and year of delivery.

Table 3 shows crude and adjusted measures of association between type of delivery facility (private or public) and caesarean section for each location. Delivering in a private health facility was associated with an increased odds of caesarean section in all but one location (rural India). The relative odds of a caesarean in a private facility were greatest in Bangladesh (OR: 6.82, 95% CI: 5.96, 7.81), followed by Nepal (OR: 2.42, 95% CI: 1.48, 3.94) and urban India (OR 1.36, 95% CI: 1.21, 1.52). These associations persisted and were only mildly attenuated when adjusted for maternal characteristics, pregnancy and delivery characteristics, and year of delivery (Bangladesh AOR: 5.91, 95% CI: 5.15, 6.78; Nepal AOR: 2.37, 95% CI: 1.62, 3.44; urban India AOR: 1.22, 95% CI: 1.09, 1.38).

Women who had four or more antenatal check-ups were significantly more likely to have a caesarean delivery in rural Bangladesh and Nepal, with positive but non-significant trends in all other locations. Parity was not associated with caesarean delivery in any location. Having a serious health complication during pregnancy and delivery was associated with caesarean delivery in all locations except rural Bangladesh, where we observed a negative association (AOR: 0.87, 95% CI: 0.76, 1.00). Multiple birth was associated with an increased odds of caesarean section only in urban India and in rural Nepal. Higher maternal age was only associated with an increased odds of caesarean section in urban India. Maternal education was only associated with an increased odds of caesarean delivery in rural Bangladesh and urban India, with mothers with secondary education or Bachelor's degrees having higher odds (AOR: 1.44, 95% CI: 1.13, 1.84 and AOR 2.44, 95% CI: 1.52, 3.92 for Bangladesh and AOR: 1.22, 95% CI: 1.04, 1.42 and 1.62, 95% CI: 1.30, 2.02 for urban India, respectively). There were significant positive associations only in rural Bangladesh, with small increases in odds of caesarean section for unit increase in wealth quartile (AOR comparing wealthiest to poorest group in Bangladesh: 1.36, 95% CI: 1.09,1.70), and in urban India, for the wealthiest quartile (AOR: 1.50, 95% CI: 1.27, 1.78).

We found interactive associations between maternal education and private facility delivery in two of four sites, with including an interaction improving model fit in two of the four analyses -(p=0.021 in rural Bangladesh and p<0.001 in urban India; **table 4** and supplemental Table 2). In Bangladesh, there was a pronounced positive educational gradient in caesarean delivery within those who delivered in private facilities, as well as a clear positive association between private facility delivery and caesarean delivery. Women with bachelor's degrees delivering in private facilities had 11 times greater odds of delivering by caesarean (AOR 11.09, 95% CI: 6.28, 19.57) than did women with no formal education delivering in public facilities. Women with no formal education delivering in private facilities had nearly seven times the odds of caesarean compared with the reference group (AOR 6.94, 95% CI: 4.64, 10.39). A similar

For peer review only - http://bmjopen.bmj.com/site/about/guidelines.xhtml

positive gradient was seen among women delivering in public facilities, with women with bachelor's education having four times greater odds of caesarean delivery in public facilities than women with no formal education (AOR 4.55, 95% CI: 2.22, 9.33). There was also an educational gradient among women delivering in private facilities in urban India. Educated mothers delivering in private facilities were more likely to deliver by caesarean than were mothers in other groups (AOR 2.10; 95% CI: 1.61, 2.75). However, there was no apparent gradient by educational attainment among women delivering in public facilities.

DISCUSSION

Our analysis of data from over 45,000 births confirms the findings of other studies identifying differences in caesarean rates between public and private facilities, and suggests that, even in underserved areas in South Asia, caesareans without medical indication are of concern. In three of four locations, rates of caesarean section were higher in private/charitable facilities than in public facilities. The findings from Bangladesh are particularly noteworthy as they show much greater odds of caesarean section in private facilities, concurring with previous analyses.[37] This was also the only location where serious complications in pregnancy and delivery were not associated with caesarean delivery, suggesting that obstetric surgery was performed over and above clinical need.

Our findings confirm the results of earlier studies of the prevalence of caesarean delivery in South Asia, and indicate that high rates can be found in underserved rural areas. In India, a recent analysis of 2010-11 Annual Health Survey (AHS) data from 284 districts in nine States, including Jharkhand, found that the median caesarean section rate in the private sector was 28%, compared with 5% in the public sector.[38] There appear to be strong financial incentives for surgical procedures in the private sector.[39] In a rural, largely indigenous part of eastern India where more mothers delivered in private/charitable facilities than in public facilities, more caesarean sections were performed in the public sector. In this particular setting, our field experience suggests that only women with serious complications would go to a facility and have a caesarean section, especially as JSY was not yet available in the study areas at the time of data collection. Such women are likely to have experienced multiple referrals from either ill-equipped public or private facilities not wanting to take the risk of admission. It is however possible that preference for the private sector has changed in Jharkhand since the advent of JSY, and further disaggregated analyses of Annual Health Survey data would allow a more contemporary exploration of State-level variations in caesarean section rates between public and private sectors.

In Mumbai slums with a high uptake of institutional births and a 60/40 split in favour of the public sector for delivery care, we found no difference in caesarean section rates between the two sectors, which is

For peer review only - http://bmjopen.bmj.com/site/about/guidelines.xhtml

somewhat surprising given the high rates of caesareans observed in the private sector in nine other states of India (excluding Maharashtra). At least two scenarios may be relevant in this setting. First, public sector hospitals are able to provide caesarean section, while smaller private maternity homes may not be able to. Second, the costs of caesarean section borne by private sector providers are higher in Mumbai, and families may be unwilling to bear these additional costs. This provides motivation for private providers not to provide caesareans, and makes it likely that women experiencing complications move into the public sector. The variety of facility options in an urban environment means that mothers are able to choose between different types of facilities, while in rural areas only one provider may be accessible.

Analyses using the most recent Bangladesh DHS found that, in 2011, three in five facility births were delivered by caesarean section. This reflects a historical trend: in 2001-2003, nearly half of deliveries in private facilities in Bangladesh were already by caesarean section.[18] Our data from rural, socio-economically disadvantaged communities in three districts of Bangladesh confirm these population-level findings, but also suggest that high caesarean section rates in private facilities are not merely an issue for wealthy urban mothers. Although some research from Bangladesh suggests that mothers may have a preference for caesarean delivery because of fear of labour pain or a desire to select an auspicious date for the birth, other studies also highlight women's fears of caesarean section and their distrust of health providers who recommend them, mainly because of the high costs associated with the procedure.[37 40 41] A qualitative study involving twenty women who had experienced obstetric complications in Matlab in 2008-9 found that most of the fourteen women who had undergone caesareans had spent over Tk 14,999 (US$ 217) on the procedure, which was approximately one-third of GDP per capita at the time.[41] The lack of association between caesareans and complications in pregnancy or delivery and multiple births, coupled with the high financial incentives given to providers for performing caesarean sections and the requirement for junior doctors to 'practise' their surgical skills, further suggest that obstetric surgery is being used over and above clinical need. A possible explanation for the significant interactive associations between maternal education and caesarean delivery in both public and private facilities in Bangladesh is that well-educated women may be delivering in more expensive or highly rated institutions, which may in turn be more likely to do caesarean sections for financial reasons and if they act as training centres for junior doctors.

In our sample of institutional deliveries from rural Nepal, 16% of facility births were by caesarean, which is higher than the national average: the 2011 Nepal DHS found an overall national caesarean section rate of 5%, with sections more commonly performed for births to highly educated mothers (13%) and mothers in the highest wealth quintile (14%).[26] It is possible that women with complications are more likely to deliver in facilities, and also that, as in Bangladesh, private providers are motivated by financial

For peer review only - http://bmjopen.bmj.com/site/about/guidelines.xhtml

For peer review only

incentives to conduct caesareans more frequently than strictly necessary. Women who can afford to seek care in private facilities may also be more willing or able to pay for caesarean sections, and providers may conduct more of them to increase their income.

A 2010 analysis of DHS data examining the role of the private sector in maternity care in 16 countries found evidence of a trend towards privatisation in delivery care between the 1990s and mid-2000s, but with strong differences between countries, which might reflect the heterogeneous nature of this sector both between and within countries. [42] This DHS study highlighted the need for more context-specific data on the nature of the private sector in low and middle-income countries, and its role in maternity care. Further research might focus on understanding the motivations and experiences of women undergoing caesarean sections in private facilities in South Asian settings, pathways for switching between public and private sectors in the event of obstetric complications, a more comprehensive tally of the financial incentives (official or non-official) that motivate private providers to carry out caesareans in each setting, and the consequences of increased caesarean sections in the private sector for maternal and neonatal health outcomes.

In our study, locations with higher prevalence of caesarean deliveries also showed a positive educational gradient even after adjusting for wealth measured by household assets. Moreover, there was an interactive association between education and type of facility, with highly educated women particularly likely to receive caesarean deliveries in private facilities in Bangladesh and urban India. Literature exploring the determinants of caesarean delivery emphasi~~s~~zes that multiple influences drive a woman's decision to deliver by caesarean. Profit considerations at the facility level may prompt some providers to urge women to receive unnecessary procedures, while women themselves may prefer caesareans for cultural reasons, fear of painful deliveries, or because they believe it to be safer. [36 43-45] Within the client-provider interaction, providers may be more likely to acquiesce to a request for caesarean from a highly educated woman; and such women may be more likely to accept advice from a provider. [19 46] Our findings lend support to the hypothesis that, while provider-level factors are probably partially responsible for the rapid increase in caesarean deliveries, it is also necessary to consider women's own preferences and decision-making processes and how they are shaped by social and cultural factors.

Strengths and limitations

The strengths of the study were its large sample size and focus on underserved communities, which are a priority for public health interventions in South Asia. It had five main limitations. It was not a nationally representative study, and districts or clusters were sampled purposively from previous cRCTs. This limits the generalisability of our findings to geographical settings outside the study areas. The study was cross-sectional, and therefore only able to suggest associations rather than causal relationships. The data also

For peer review only

For peer review only - http://bmjopen.bmj.com/site/about/guidelines.xhtml

did not include key predictors of caesarean section such as breech presentation, and whether the previous delivery was by caesarean, which limits the completeness of our analysis of determinants. In addition, levels of serious complications in pregnancy and delivery varied considerably between locations and were often higher than expected, suggesting potential over-reporting and limited reliability as an indicator of complications. Finally, some variables had small denominators, and others had high levels of missing data (for example, maternal age in rural India).

Conclusions

Our study found that delivering in a private health facility was associated with an increased odds of caesarean section in three of four South Asian locations, and that the associations persisted after adjustments for maternal, pregnancy and delivery characteristics, and year of delivery. We also found significant interactive associations between maternal education and caesarean delivery in Bangladesh (both private and public facilities) and urban India (private facilities only). These results lend support to the hypothesis that increased caesarean section rates in these three South Asian countries may in part be driven by the private sector, but also suggest that, in some settings such as Bangladesh and urban India, demand from more educated mothers may play a part. These findings call for greater, local understanding of the role of private providers in maternity care, together with careful examination of the consequences of increased caesarean sections in the private sector for maternal and neonatal health outcomes.

Competing interests

None to declare

Contributors

MN and AP had the original idea for the study. MN designed the data analysis strategy in discussion with AP. MN, GA, AS, CS and NS prepared the data. MN performed the analysis and prepared the tables. NN, PT, NSM, DO, KA, DM and AC are responsible for the trials. AP wrote the first draft of the paper and all authors read and commented on the draft. All authors read and approved the final manuscript.

For peer review only

Funding

MN and NS are funded through a Wellcome Trust Strategic Award (085417MA/Z/08/Z). In Bangladesh, the Perinatal Care Project trial (covering KA and AK) was registered with trial number ISRCTN54792066 and has received funding from the Big Lottery Fund and the Wellcome Trust. In Eastern India, the Ekjut trial (covering NN and PT) was registered with trial number ISRCTN21817853 and funded by the Health Foundation. The Dhanusha (Nepal) trial (covering DSM, NS, BPS, and CS) was registered with trial number ISRCTN87820538 and has received funding from: the UBS Optimus Foundation; USAID; DfID and the Wellcome Trust. The City Initiative for Newborn Health trial led by SNEHA (Mumbai, India) was registered with trial number ISRCTN96256793 and has received funding from the ICICI Foundation for Inclusive Growth and the Wellcome Trust. GA, NSM, and DO are currently funded by The Wellcome Trust (091561/Z/10/Z). The funders played no part in the development of the article or the decision to submit it for publication.

Provenance and peer review

Not commissioned; externally peer reviewed.

For peer review only

REFERENCES:

1. World Health Organisation and UNICEF. Countdown 2015: Building a future for women and Children, the 2012 Report. http://countdown2015mnch.org/documents/2012Report/2012-Complete.pdf [Accessed 9th November 2013]
2. Campbell OM, Graham WJ. Strategies for reducing maternal mortality: getting on with what works. *The Lancet* 2006; 368: 1284-1299.
3. Ronsmans C, De Brouwere V, Dubourg D, *et al*.. Measuring the need for life-saving obstetric surgery in developing countries. *BJOG* 2004; 111: 1027-30.
4. World Health Organization. Appropriate technology for birth. *The Lancet* 1985; 2: 436-7.
5. Betrán AP, Merialdi M, Lauer JA, *et al*. Rates of caesarean section: analysis of global, regional and national estimates. *Paediatric and Perinatal Epidemiology* 2007; 21: 98-113.
6. Cavallaro FL, Cresswell JA, França GVA, *et al*. Trends in Caesarean delivery by country and wealth quintile: cross-sectional surveys in southern Asia and sub-Saharan Africa. *WHO Bulletin* 2013; 91: 897-972.
7. Souza JP, Gülmezoglu A, Lumbiganon P, *et al*. WHO Global Survey on Maternal and Perinatal Health Research Group. Caesarean section without medical indications is associated with an increased risk of adverse short-term maternal outcomes: the 2004–2008 WHO Global Survey on Maternal and Perinatal Health. *BMC Medicine* 2010; 8:71.
8. Kyu HH, Shannon HS, Georgiades K, Boyle MH. Caesarian delivery and neonatal mortality rates in

46 low- and middle-income countries: a propensity score matching and meta-analysis of demographic health survey data. *International Journal of Epidemiology* 2013; 1-11.

9. Gibbons L, Belizán JM, Lauer JA, *et al*. The global numbers and costs of additionally needed and unnecessary caesarean sections performed per year: overuse as a barrier to universal coverage. World Health Report (2010), Background paper. Geneva: World Health Organization.
10. Mitra SN, Nawab Ali M, Islam S, *et al*. Bangladesh Demographic and Health Survey, 1993-1994. Calverton, Maryland: National Institute of Population Research and Training (NIPORT), Mitra and Associates, and Macro International Inc, 1994.
11. National Institute of Population Research and Training (NIPORT), Mitra and Associates, and ICF International. Bangladesh Demographic and Health Survey 2011. Dhaka, Bangladesh and Calverton, Maryland, USA: NIPORT, Mitra and Associates, and ICF International, 2013.
12. International Institute for Population Sciences (IIPS) and Macro International. National Family Health Survey (NFHS 1), 1992–93. India, Mumbai: IIPS, 1995.
13. International Institute for Population Sciences (IIPS), Macro International. National Family Health Survey (NFHS 3), 2005–06; India: Volume I. Mumbai: IIPS, 2007.
14. Pradhan A, Aryal RH, Regmi G, *et al*. Nepal Family Health Survey 1996. Kathmandu, Nepal and Calverton, Maryland: Ministry of Health [Nepal], New ERA, and Macro International Inc, 1997.
15. Ministry of Health and Population (MOHP) [Nepal], New ERA, and ICF International Inc. Nepal Demographic and Health Survey 2011. Kathmandu, Nepal: Ministry of Health and Population, New ERA, and ICF International, Calverton, Maryland, 2012.
16. Ajeet S, Nandkishore K. The boom in unnecessary caesarean surgeries is jeopardizing women's health. *Health Care Women International* 2013; 34: 513-521.
17. Sreevidya S, Sathiyasekaran BW. High caesarean rates in Madras (India): a population-based cross-sectional study. *BJOG* 2003; 110: 106-111.
18. Koblinksi M, Anwar I, Mridha MK, *et al*. Reducing maternal mortality and improving maternal health: Bangladesh and MDG 5. *J Health Popul Nutr* 2008; 26: 280-294.
19. Leone T, Padmadas SS, Matthews Z. Community factors influencing rising caesarean section rates in developing countries: an analysis of six countries. *Soc Sci Med* 2008; 67: 1236-46.
20. Mazzoni A, Althabe A, Liu NH, *et al*. Women's preference for caesarean section: a systematic review and meta-analysis of observational studies. *BJOG* 2011; 118: 391-399.
21. Tripathy P, Nair N, Barnett S, et al. Effect of a participatory intervention with women's groups on birth outcomes and maternal depression in Jharkhand and Orissa, India: A cluster-randomised controlled trial. *Lancet* 2010; 375: 1182–1192.
22. Azad K, Barnett S, Banerjee B, *et al*. Effect of scaling up women's groups on birth outcomes in three rural districts in Bangladesh: a cluster-randomised controlled trial. *Lancet* 2010; 375: 1193–1202.

For peer review only - http://bmjopen.bmj.com/site/about/guidelines.xhtml

23. Fottrell E, Azad K, Kuddus A, *et al*. The effect of increased coverage of participatory women's groups on neonatal mortality in Bangladesh: a cluster-randomised trial. *JAMA Pediatrics* 2013; 167: 816-825.

24. Shah More N, Bapat U, Das S, *et al*. Cluster-randomised controlled trial of community mobilisation in Mumbai slums to improve care during pregnancy, delivery, postpartum and for the newborn. *Trials* 2008; 9:7.

25. Shrestha BP, Bhandari B, Manandhar DS, *et al*. Community interventions to reduce child mortality in Dhanusha, Nepal: study protocol for a cluster randomized controlled trial. *Trials* 2011; 12: 136.

26. Prost A, Colbourn T, Seward N, *et al*. Women's groups practising participatory learning and action to improve maternal and newborn health in low-resource settings: a systematic review and meta-analysis. *Lancet* 2013; 381: 1736–46.

27. Schmidt J-0, Ensor T, Hossain A, Khan S. Vouchers as demand side financing instruments for health care: A review of the Bangladesh maternal voucher scheme. *Health Policy* 2010; 96: 98–107.

28. Ahmed S, Khan MM. A maternal health voucher scheme: what have we learned from the demand-side financing scheme in Bangladesh? *Health Policy & Planning* 2011; 6:25–32.

29. Lim SS, Dandona L, Hoisington JA, *et al*. India's Janani Suraksha Yojana, a conditional cash transfer programme to increase births in health facilities: an impact evaluation**.** *Lancet* 2010; 375: 209-23.

30. Shah More N, Alcock G, Bapat U, *et al*. Tracing pathways from antenatal to delivery care for women in Mumbai, India: cross-sectional study of maternity in low-income areas. International Health 2009; 1: 71–77.

31. Government of India, Ministry of Health & Family Welfare. Janani Suraksha Yojana: Guidelines for Implementation, 2006. http://www.mohfw.nic.in/NRHM/./guidelines/JSY_guidelines_09_06.pdf

32. Witter S, Khadka S, Nath H, Tiwari S. The national free delivery policy in Nepal: early evidence of its effects on health facilities. *Health Policy and Planning* 2011; 26: ii84–ii91.

33. Holgado-Tello FP,Chacón–Moscoso S, Barbero-Garcia I, Vila-Abad E. Polychoric versus Pearson correlations in exploratory and confirmatory factor analysis of ordinal variables. *Quality & Quantity* 2010; 44 (1): 153-166.

34. Rabe-Hesketh S, Skrondal A, Pickles A. Maximum likelihood estimation of limited and discrete dependent variable models with nested random effects. *Journal of Econometrics* 2005; 128: 301-323.

35. StataCorp. 2013. *Stata Statistical Software: Release 13*. College Station, TX: StataCorp LP.

36. Potter J, Berquó E, Perpétuo I, *et al*. Unwanted Caesarean Sections among Public and Private

For peer review only - http://bmjopen.bmj.com/site/about/guidelines.xhtml

Patients in Brazil: Prospective Study. *BMJ* 2001; 323: 1155-8.

37. Kamal SMM. Preference for institutional delivery and caesarean sections in Bangladesh. *J Health Popul Nutr* 2013; 31: 96-109.
38. Randive B, Diwan V, De Costa A. India's Conditional Cash Transfer Programme (the JSY) to Promote Institutional Birth: Is There an Association between Institutional Birth Proportion and Maternal Mortality? *PLoS ONE* 2013; 8(6): e67452. doi:10.1371/journal.pone.0067452
39. Chatterjee S, Laxminarayan R. Costs of surgical procedures in Indian hospitals. *BMJ Open* 2013; 3: e002844.
40. Parkhurst JO, Rahman SA. Life saving or money wasting? Perceptions of caesarean sections among users of services in Rural Bangladesh. *Health Policy* 2007; 80: 392-401.
41. Khan R, Blum LS, Sultana M, et al. An Examination of Women Experiencing Obstetric Complications Requiring Emergency Care: Perceptions and Sociocultural Consequences of Caesarean Sections in Bangladesh. *J Health Popul Nutr 2012*; 30: 159-171.
42. Pomeroy A, Koblinksy M, Alva S. Private Delivery Care in Developing Countries: Trends and Determinants. DHS Working document 2010; No.76. http://dhsprogram.com/pubs/pdf/WP76/WP76.pdf [Accessed 3rd March 2014].
43. Sufang G, Padmadas SS, Fengmin Z et al. Delivery settings and caesarean section rates in China. *Bull WHO* 2007; 85: 733-820.
44. Lo JC. Patients' attitudes vs. physicians' determination: implications for cesarean sections. *Soc Sci Med* 2003; 57: 91-6.
45. McCourt C, Weaver J, Statham H, *et al.* Elective cesarean section and decision making: a critical review of the literature. *Birth* 2007; 34: 65-79.
46. Ghetti C, Chan BKS, Guise J-M. Physicians' responses to patient-requested caesarean delivery. *Birth* 2004; 31: 280-4.

For peer review only

For peer review only - http://bmjopen.bmj.com/site/about/guidelines.xhtml

Table 1: Characteristics of studies and populations

**Study (Country)**	**Bangladesh (rural)**	**India (rural)**	**Nepal (rural)**	**India (urban)**
**Location**	Three districts: Bogra, Maulvibazaar and Faridpur	Three districts of Jharkhand and Odisha: Keonjhar, West Singhbhum and Saraikela	Dhanusha district (Terai)	Mumbai slums
**Period**	2005 – 2011	2005-2008	2008-2011	2006-2009
**Estimated population**	532,900	114,000	240,000	283,000
**Cluster characteristics**	Villages making up a union	8-10 villages with residents classified as Scheduled Tribe or Other Backward Class	Village Development Committee	Slum areas in six municipal wards of Mumbai
**Method of cluster identification**	Purposive sampling of three districts and clusters within districts	Purposive sampling of three districts and clusters within districts	Random sampling of 60 clusters from a list of 79 suitable clusters in one district	92 clusters in six municipal wards identified using municipal documents, surveys, discussions with key informants, and site visits. Random selection of 48 clusters for randomized allocation.
**Clusters, n**	9	18	30	24
**Cluster and individual follow-up**	All clusters followed up<br>Interviews completed after 82% of identified births in control areas in Phase 1, and 99% of births in Phase 2	All clusters followed up<br>Interviews completed after 98% of identified births	All clusters followed up	All clusters followed up<br>Interviews completed after 83% of identified births
**Maternal mortality ratio**	254.3	668.1	Unknown	206.2
**Health facilities available in control areas**	Public facilities: District Hospitals; Maternal and Child Welfare Centres; Upazilla Health Complexes. Private facilities: small-to-medium-size clinics; BRAC (NGO) facilities where deliveries do not take place; larger private hospitals with and without Comprehensive emergency obstetric care (CEmOC) facilities.	Public facilities: District Hospitals; Primary Health Centres (PHCs) in which deliveries can notionally take place but that are not usually equipped for CEmOC; Community Health Centres (CHCs) acting as referral centres for PHCs, covering a population of around 80,000 with EmOC facilities; district hospitals. Private and charitable facilities: medium-sized missionary hospitals with EmOC facilities.	Public facilities: three Primary Health Care Centres, three Health Posts and 24 Sub-Health Posts, none of which are equipped for CEmOC. These health facilities refer to the public Zonal Tertiary Hospital and various private providers in the district headquarters and nearby medical college, which have facilities for caesarean sections.	Public facilities: Municipal tertiary hospitals, general hospitals, and maternity homes. Private facilities: specialty hospitals, general hospitals, and maternity homes.

For peer review only - http://bmjopen.bmj.com/site/about/guidelines.xhtml

Table 2: Characteristics of institutional births and caesarean deliveries by location

	Births	Institutional births						Caesarean section births			
		All		Public facility		Private/charitable facility		Public facility		Private/charitable facility	
	(n)	(n)	(% all births)	(n)	(% institutional births)	(n)	(% institutional births)	(n)	(% deliveries in public facilities)	(n)	(% deliveries in private facilities)
**Bangladesh (rural)**	21,560	4,592	21%	2,053	45%	2,539	55%	589	29%	1,852	73%
**India (rural)**	8,541	1,816	21%	421	23%	1,395	77%	64	15%	66	5%
**India (urban)**	10,236	9,259	90%	5,489	59%	3,770	41%	811	15%	697	18%
**Nepal (rural)**	4,931	1,586	32%	1,424	90%	162	10%	201	14%	48	30%

* Nepal numbers weighted using women's probability of selection within each cluster, scaled to total number of institutional births. (Unweighted Nepal totals: 4,990 births and 1,586 institutional births)

For peer review only

Table 3: Mutually adjusted associations of type of facility of delivery and other selected determinants with caesarean section delivery, by location

	Bangladesh (rural) ORs (95% CIs)		India (rural) ORs (95% CIs)		India (urban) ORs (95% CIs)		Nepal (rural)* ORs (95% CIs)	
	Crude	Adjusted	Crude	Adjusted	Crude	Adjusted	Crude	Adjusted
**Health facility characteristics**								
Public health facility (ref)								
Private health facility	6.82 (5.96,7.81)	5.91 (5.15,6.78)	0.39 (0.25,0.61)	0.46 (0.29,0.73)	1.36 (1.21,1.52)	1.22 (1.09,1.38)	2.42 (1.48,3.94)	2.37 (1.62,3.44)
**Pregnancy and delivery characteristics**								
4+ antenatal care visits (fewer or none = ref)**		1.46 (1.26,1.69)		1.49 (0.96,2.32)		1.06 (0.93,1.21)		1.92 (1.43,2.58)
Birth order								
1 (ref)								
2		0.95 (0.80,1.13)		1.25 (0.80,1.95)		0.65 (0.56,0.75)		1.41 (1.02,1.94)
3		1.19 (0.94,1.52)		0.36 (0.15,0.87)		0.60 (0.50,0.71)		0.58 (0.37,0.92)
4+		1.17 (0.90,1.53)		0.36 (0.15,0.85)		0.39 (0.32,0.48)		1.00 (0.54,1.84)
Serious complications in pregnancy/delivery*** (no complications=ref)		0.87 (0.76,1.00)		1.77 (1.17,2.67)		1.71 (1.39,2.11)		4.87 (2.51,9.47)
Multiple birth (ref=single)		0.93 (0.65,1.32)		1.57 (0.52,4.75)		3.01 (2.14,4.23)		3.42 (1.77,6.61)
**Maternal characteristics**								
Age (years)								
15-24 (ref)								
25-34		1.12 (0.94,1.34)		1.81 (1.14,2.86)		1.45 (1.27,1.66)		1.52 (0.63,1.94)
35+		1.10 (0.77,1.58)		2.11 (0.63,7.07)		1.79 (1.27,2.53)		1.55 (0.55,1.47)

For peer review only

For peer review only - http://bmjopen.bmj.com/site/about/guidelines.xhtml

	Bangladesh (rural) ORs (95% CIs)		India (rural) ORs (95% CIs)		India (urban) ORs (95% CIs)		Nepal (rural)* ORs (95% CIs)	
	Crude	Adjusted	Crude	Adjusted	Crude	Adjusted	Crude	Adjusted
Maternal education****								
No formal education (ref)								
Primary education		1.07		0.98		1.13		0.90
		(0.83,1.38)		(0.39,2.47)		(0.85,1.49)		(0.59,1.39)
Secondary education		1.44		1.11		1.22		1.37
		(1.13,1.84)		(0.68,1.80)		(1.04,1.42)		(0.97,1.94)
Bachelor's degree or higher		2.44		1.20		1.62		-
		(1.52,3.92)		(0.43,3.31)		(1.30,2.02)		-
Household wealth quintile								
1st (poorest, ref)								
2nd		1.31		1.61		1.14		1.11
		(1.06,1.63)		(0.69,3.75)		(0.77,1.68)		(0.63,1.94)
3rd		1.41		1.36		1.13		0.90
		(1.10,1.82)		(0.58,3.21)		(0.95,1.33)		(0.55,1.47)
4th (wealthiest)		1.36		2.16		1.50		0.99
		(1.09,1.70)		(0.87,5.33)		(1.27,1.78)		(0.52,1.90)
Year of delivery								
2004-2006 (ref: BD, IN)								-
2007-2009 (ref: NP rural)		0.90		0.90		1.14		-
		(0.74,1.10)		(0.61,1.33)		(1.09,1.38)		-
2010-2012		1.15		-		-		1.29
		(0.94,1.41)		-		-		(0.91,1.82)
N	4,592	4,592	1,816	1,816	9,259	9,259	1,586	1,586

* Nepal numbers weighted using women's probability of selection within each cluster, scaled to total number of institutional births.

** At least one visit with skilled provider

***Includes: symptoms of eclampsia (fits, seizures, convulsions, or unconsciousness during pregnancy or delivery); reduced or no fetal movement; labour lasting more than 24 hours.

****For the Nepal data, two respondents with bachelor's degrees (2 respondent total, 1 delivering in institution) were combined with respondents with secondary education.

All analyses additionally adjusted for survey design using fixed effect of stratum and random effect of cluster.

For peer review only

Table 4: Interactive associations between type of delivery facility, maternal education, and caesarean delivery, by location

	Bangladesh (rural)		India (rural)		India (urban)		Nepal (rural)*	
	(n)	AOR, 95% CI	(n)	AOR, 95% CI	(n)	AOR, 95% CI	(unweighted n)	AOR, 95% CI
Public facility - no maternal education (ref)	202	-	109	-	1,396	-	822	-
Public facility - primary education	448	1.07 (0.76,1.50)	17	0.92 (0.18,4.77)	357	0.88 (0.61,1.27)	196	1.04 (0.65,1.65)
Public facility - secondary education	202	1.63 (1.18,2.24)	604	1.09 (0.52,2.26)	920	1.1 (0.91,1.33)	413	1.45 (1.04,2.02)
Public facility - bachelor's degree	448	4.58 (2.24,9.38)	100	1.55 (0.41,5.89)	180	1.06 (0.76,1.47)	-	- -
Private facility - no education	332	7.24 (4.82,10.87)	109	0.46 (0.21,1.01)	1396	0.91 (0.70,1.19)	100	2.83 (1.73,4.61)
Private facility - primary education	578	7.58 (5.36,10.71)	17	0.47 (0.14,1.58)	357	1.52 (0.99,2.33)	16	0.86 (0.14,5.21)
Private facility - secondary education	1,102	9.03 (6.57,12.41)	274	0.52 (0.24,1.12)	3,382	1.3 (1.06,1.60)	39	3.01 (1.71,5.30)
Private facility - bachelor's degree	41	11.24 (6.37,19.85)	21	0.4 (0.08,2.00)	354	2.1 (1.61,2.75)	-	- -
N	3,353		1,251		8,342		1586	
p-value**	0.021		0.792		0.001		0.394	

All results adjusted for number of antenatal care visits, parity, medical indication for caesarean, multiple birth, maternal age (10-yr group), household assets, stratum, and cluster (random effect).
* Nepal numbers weighted using women's probability of selection within each cluster, scaled to total number of institutional births.

** p-value from -2 log-likelihood test comparing nested models with and without interaction terms

For peer review only - http://bmjopen.bmj.com/site/about/guidelines.xhtml

"Prevalence and determinants of caesarean section in private and public health facilities in underserved South Asian communities: cross-sectional analysis of data from Bangladesh, India, and Nepal"

Supplemental tables

Supplemental Table 1: Characteristics of institutional deliveries by characteristics of the mother, the delivery facility, and location

For peer review only

	Bangladesh (rural)				India (rural)				India (urban)				Nepal (rural)			
	Not caesarean	Caesarean	Total		Not caesarean	Caesarean	Total		Not caesarean	Caesarean	Total		Not caesarean	Caesarean	Total	
	(%)	(%)	(n)	(%)	(%)	(%)	(n)	(%)	(%)	(%)	(n)	(%)	(%)	(%)	(n)	(%)
**Health facility characteristics**																
Public health facility	68.1	24.1	2053	44.7	21.2	49.2	421	23.2	60.4	53.8	5489	59.3	91.5	80.7	1424	89.8
Private/charitable health facility	31.9	75.9	2539	55.3	78.8	50.8	1395	76.9	39.6	46.2	3770	40.7	8.5	19.3	162	10.2
**Pregnancy and delivery characteristics**																
4+ antenatal care visits*	25.2	38.8	1491	67.5	47.1	56.2	867	52.3	73.4	74.2	6810	26.4	41.3	53.6	686	43.2
Birth order																
1	47.1	47.4	2170	47.3	51.6	55.4	942	51.9	29.7	41.6	2932	31.7	50.1	46.7	786	49.6
2	25.9	26.1	1194	26	23	32.3	430	23.7	27.9	26.1	2554	27.6	22.5	27.1	368	23.2
3	12.4	13.8	603	13.1	11.7	5.4	204	11.2	19.4	17.6	1769	19.1	14.9	10.3	225	14.2
4+	14.6	12.7	625	13.6	13.7	6.9	240	13.2	23	14.7	2004	21.6	12.5	15.9	207	13
Serious complications in pregnancy/delivery**	53.4	43.5	2212	48.2	29.2	44.6	550	30.3	6.1	9.2	608	6.6	21.2	38.5	379	23.9
Multiple birth	3.6	3.9	173	3.8	2.1	3.1	40	2.2	1.4	3.6	163	1.8	2.2	7.8	48	3.1
**Maternal socio-demographic characteristics**																
Maternal age (years)																
15-24	59.8	55.8	2650	57.7	63.6	60	1150	63.3	53.1	51.5	4894	52.9	71.6	66.3	1123	70.8
25-34	34.4	39.5	1705	37.1	32.7	36.9	600	33	43.6	45.3	4060	43.8	26.4	31	431	27.2
35+	5.8	4.6	237	5.2	3.7	3.1	66	3.6	3.3	3.2	305	3.3	1.9	2.7	33	2.1
Maternal education																
No formal education	15.2	8.3	531	11.6	40.1	28.5	713	39.3	26.3	18.5	2316	25	58.4	57.2	924	58.2
Primary education	27.3	17.9	1025	22.3	6.6	4.6	117	6.4	6	4.9	537	5.8	13.6	10.8	209	13.2
Secondary education	55.7	68.6	2872	62.5	50.2	62.3	927	51	59.6	61.9	5550	59.9	28	32	453	28.6
Bachelor's degree or higher***	1.7	5.2	164	3.6	3.1	4.6	59	3.2	8.2	14.7	856	9.2	-	-	-	-

Household wealth quintile																
1st (poorest)	23	10.4	749	16.3	12.6	5.4	220	12.1	24.1	17.2	2125	23	14.2	13.4	223	14.1
2nd	32.4	31.5	1465	31.9	30	31.5	547	30.1	3	2.3	268	2.9	22	22.4	350	22.1
3rd	14	15.2	673	14.7	39.9	32.3	715	39.4	35.9	31.3	3254	35.1	33.9	30.5	529	33.4
4th (wealthiest)	30.5	42.9	1705	37.1	17.4	30.8	334	18.4	37	49.3	3612	39	29.9	33.6	483	30.5
**Year of delivery**																
2004-2006	19.2	15.2	782	17	36.7	40.8	672	37	46.4	42.2	4237	45.8	-	-	-	-
2007-2009	44.7	38.7	1907	41.5	63.3	59.2	1144	63	53.6	57.8	5022	54.2	45.1	45.7	717	45.2
2010-2012	36.1	46.1	1903	41.4	-	-	-	-	-	-	-	-	54.9	54.3	869	54.8
Total (n)	2151	2441	4592	100	1686	130	4592	100	7751	1508	9259	100	734	135	869	100

* Nepal numbers weighted using women's probability of selection within each cluster, scaled to total number of institutional births.

** At least one visit with skilled provider

***Includes: symptoms of pre-eclampsia (swollen face or limbs or blurred vision [not asked in Nepal]), eclampsia (fits, seizures, convulsions, or unconsciousness during pregnancy or delivery); haemorrhaging during delivery, labour lasting more than 24 hours.

****For the Nepal data, secondary and bachelor's (2 respondent total, 1 delivering in institution) were combined.

For peer review only

Supplemental Table 2: Interactive associations between caesarean delivery and mother's educational attainment and place of delivery by location

	Bangladesh (rural) OR, 95% CI	India (rural) OR, 95% CI	India (urban) OR, 95% CI	Nepal (rural) OR, 95% CI
Main associations				
Private delivery facility (public = ref)	7.24	0.47	0.91	2.83
	(4.82,10.87)	(0.21,1.02)	(0.70,1.19)	(1.43,2.56)
Mother's educational attainment (no education = ref)				
Primary education	1.07	0.91	0.88	1.04
	(0.76,1.50)	(0.18,4.75)	(0.61,1.27)	(0.65,1.65)
Secondary education	1.63	1.09	1.1	1.45
	(1.18,2.24)	(0.52,2.27)	(0.91,1.33)	(1.04,2.02)
Bachelor's or higher	4.59	1.55	1.06	-
	(2.24,9.40)	(0.41,5.88)	(0.76,1.47)	-
Interactive associations				
Primary education * private delivery	0.98	1.11	1.9	0.29
	(0.60,1.61)	(0.15,8.12)	(1.07,3.37)	(0.04,1.92)
Secondary education * private delivery	0.77	1.03	1.31	0.74
	(0.49,1.19)	(0.42,2.51)	(0.97,1.77)	(0.31,1.73)
Bachelor's or higher * private delivery	0.34	0.55	2.18	-
	(0.14,0.83)	(0.08,3.93)	(1.42,3.35)	-
N	4590	1812	9259	1586

For peer review only

STROBE 2007 (v4) checklist of items to be included in reports of observational studies in epidemiology*
Checklist for cohort, case-control, and cross-sectional studies (combined)

Section/Topic	Item #	Recommendation	Reported on page #
**Title and abstract**	1	(*a*) Indicate the study's design with a commonly used term in the title or the abstract	1
		(*b*) Provide in the abstract an informative and balanced summary of what was done and what was found	2
**Introduction**			
Background/rationale	2	Explain the scientific background and rationale for the investigation being reported	4
Objectives	3	State specific objectives, including any pre-specified hypotheses	4
**Methods**			
Study design	4	Present key elements of study design early in the paper	1, 5-6
Setting	5	Describe the setting, locations, and relevant dates, including periods of recruitment, exposure, follow-up, and data collection	5 and Table 1
Participants	6	(*a*) *Cohort study*—Give the eligibility criteria, and the sources and methods of selection of participants. Describe methods of follow-up<br>*Case-control study*—Give the eligibility criteria, and the sources and methods of case ascertainment and control selection. Give the rationale for the choice of cases and controls<br>*Cross-sectional study*—Give the eligibility criteria, and the sources and methods of selection of participants	6
		(*b*) *Cohort study*—For matched studies, give matching criteria and number of exposed and unexposed<br>*Case-control study*—For matched studies, give matching criteria and the number of controls per case	
Variables	7	Clearly define all outcomes, exposures, predictors, potential confounders, and effect modifiers. Give diagnostic criteria, if applicable	7
Data sources/ measurement	8*	For each variable of interest, give sources of data and details of methods of assessment (measurement). Describe comparability of assessment methods if there is more than one group	7
Bias	9	Describe any efforts to address potential sources of bias	7-8
Study size	10	Explain how the study size was arrived at	6
Quantitative variables	11	Explain how quantitative variables were handled in the analyses. If applicable, describe which groupings were chosen and why	7
Statistical methods	12	(*a*) Describe all statistical methods, including those used to control for confounding	7-8
		(*b*) Describe any methods used to examine subgroups and interactions	Table 4
		(*c*) Explain how missing data were addressed	7
		(*d*) *Cohort study*—If applicable, explain how loss to follow-up was addressed<br>*Case-control study*—If applicable, explain how matching of cases and controls was addressed	

For peer review only

		Cross-sectional study—If applicable, describe analytical methods taking account of sampling strategy	
		(*e*) Describe any sensitivity analyses	N/A
**Results**			
Participants	13*	(a) Report numbers of individuals at each stage of study—eg numbers potentially eligible, examined for eligibility, confirmed eligible, included in the study, completing follow-up, and analysed	7, 8
		(b) Give reasons for non-participation at each stage	7, 8
		(c) Consider use of a flow diagram	Not used
Descriptive data	14*	(a) Give characteristics of study participants (eg demographic, clinical, social) and information on exposures and potential confounders	Supplemental Table 1
		(b) Indicate number of participants with missing data for each variable of interest	
		(c) *Cohort study*—Summarise follow-up time (eg, average and total amount)	
Outcome data	15*	*Cohort study*—Report numbers of outcome events or summary measures over time	
		Case-control study—Report numbers in each exposure category, or summary measures of exposure	
		Cross-sectional study—Report numbers of outcome events or summary measures	Table 3
Main results	16	(*a*) Give unadjusted estimates and, if applicable, confounder-adjusted estimates and their precision (eg, 95% confidence interval). Make clear which confounders were adjusted for and why they were included	Table 3
		(*b*) Report category boundaries when continuous variables were categorized	
		(*c*) If relevant, consider translating estimates of relative risk into absolute risk for a meaningful time period	
Other analyses	17	Report other analyses done—eg analyses of subgroups and interactions, and sensitivity analyses	Table 4
**Discussion**			
Key results	18	Summarise key results with reference to study objectives	10
Limitations	19	Discuss limitations of the study, taking into account sources of potential bias or imprecision. Discuss both direction and magnitude of any potential bias	12
Interpretation	20	Give a cautious overall interpretation of results considering objectives, limitations, multiplicity of analyses, results from similar studies, and other relevant evidence	10-1
Generalisability	21	Discuss the generalisability (external validity) of the study results	12
**Other information**			
Funding	22	Give the source of funding and the role of the funders for the present study and, if applicable, for the original study on which the present article is based	13

*Give information separately for cases and controls in case-control studies and, if applicable, for exposed and unexposed groups in cohort and cross-sectional studies.

Note: An Explanation and Elaboration article discusses each checklist item and gives methodological background and published examples of transparent reporting. The STROBE checklist is best used in conjunction with this article (freely available on the Web sites of PLoS Medicine at http://www.plosmedicine.org/, Annals of Internal Medicine at http://www.annals.org/, and Epidemiology at http://www.epidem.com/). Information on the STROBE Initiative is available at www.strobe-statement.org.

For peer review only

BMJ Open

Prevalence and determinants of caesarean section in private and public health facilities in underserved South Asian communities: cross-sectional analysis of data from Bangladesh, India, and Nepal

Journal:	*BMJ Open*
Manuscript ID:	bmjopen-2014-005982.R3
Article Type:	Research
Date Submitted by the Author:	04-Dec-2014

Complete List of Authors:	Neuman, Melissa; UCL Institute for Global Health,<br>Alcock, Glyn; UCL Institute for Global Health,<br>Azad, Kishwar; Diabetic Association of Bangladesh, Perinatal Care Project<br>Kuddus, Abdul; Diabetic Association of Bangladesh, Perinatal Care Project<br>Osrin, David; UCL Institute for Global Health,<br>Shah More, Neena; Society for Nutrition, Education and Health Action (SNEHA),<br>Nair, Nirmala; Ekjut,<br>Tripathy, Prasanta; Ekjut,<br>Sikorski, Catherine; UCL Institute for Global Health,<br>Saville, Naomi; UCL Institute for Global Health,<br>Sen, Aman; Mother and Infant Research Activities (MIRA),<br>Colbourn, Timothy; UCL Institute for Global Health,<br>Houweling, Tanja; Erasmus MC University Medical Center, Department of Public Health<br>Seward, Nadine; UCL Institute for Global Health,<br>Manandhar, Dharma; Mother and Infant Research Activities (MIRA),<br>Shrestha, Bhim; Mother and Infant Research Activities (MIRA),<br>Costello, Anthony; UCL Institute for Global Health,<br>Prost, Audrey; UCL Institute for Global Health,

**Primary Subject Heading**:	Global health
Secondary Subject Heading:	Epidemiology, Obstetrics and gynaecology
Keywords:	Epidemiology < TROPICAL MEDICINE, OBSTETRICS, PUBLIC HEALTH

SCHOLARONE™
Manuscripts

Prevalence and determinants of caesarean section in private and public health facilities in underserved South Asian communities: cross-sectional analysis of data from Bangladesh, India, and Nepal

Melissa Neuman[1], Glyn Alcock[1], Kishwar Azad[4], Abdul Kuddus[4], David Osrin[1], Neena Shah More[3], Nirmala Nair[5], Prasanta Tripathy[5], Catherine Sikorski[1], Naomi Saville[1], Aman Sen[2], Tim Colbourn[1], Tanja AJ Houweling[6], Nadine Seward[1], Dharma S Manandhar[2], Bhim P Shrestha[2], Anthony Costello[1] and Audrey Prost[1]

Author affiliations:

[1] Institute for Global Health, University College London, 30 Guilford Street, London WC1N 1EH, UK

[2] Mother and Infant Research Activities (MIRA), YB Bhavan, Thapathali, GPO Box 921, Kathmandu, Nepal

[3] Society for Nutrition, Education and Health Action (SNEHA), Urban Health Centre, Shahunagar, Dharavi, Mumbai 400017, Maharashtra, India

[4] Perinatal Care Project, Diabetic Association of Bangladesh, 122 Kazi Nazrul Islam Avenue, Dhaka 1000, Bangladesh

[5] Ekjut, Plot 556B, Potka, Chakradharpur, West Singhbhum, Jharkhand, India

[6] Department of Public Health, Erasmus MC University Medical Center Rotterdam, P.O. Box 2040, 3000 CA Rotterdam, The Netherlands

Proposed running head: Caesarean sections in private vs. public health facilities in South Asia

Word count: 4393

Figures and tables: 0 figures and 4 tables

For peer review only

For peer review only - http://bmjopen.bmj.com/site/about/guidelines.xhtml

ABSTRACT (Max: 300 words; Current: 300)

Objectives: To describe the prevalence and determinants of births by caesarean section in private and public health facilities in underserved communities in South Asia.

Design: Cross-sectional study.

Setting: 81 community-based geographical clusters in four locations in Bangladesh, India and Nepal (three rural, one urban).

Participants: 45,327 births occurring in the study areas between 2005 and 2012.

Outcome measures: proportion of caesarean section delivery by location and type of facility; determinants of caesarean section delivery by location.

Results: Institutional delivery rates varied widely between settings, from 21% in rural India to 90% in urban India. The proportion of private and charitable facility births delivered by caesarean section was 73% in Bangladesh, 30% in rural Nepal, 18% in urban India, and 5% in rural India. The odds of caesarean section were greater in private and charitable health facilities than in public facilities in three of four study locations, even when adjusted for pregnancy and delivery characteristics, maternal characteristics, and year of delivery (Bangladesh AOR: 5.91, 95% CI: 5.15-6.78; Nepal AOR: 2.37, 95% CI: 1.62-3.44; urban India AOR: 1.22, 95% CI: 1.09-1.38). We found that highly educated women were particularly likely to deliver by caesarean in private facilities in both urban India (AOR 2.10; 95% CI: 1.61, 2.75) and rural Bangladesh (AOR 11.09, 95% CI: 6.28, 19.57).

Conclusions:

Our results lend support to the hypothesis that increased caesarean section rates in these South Asian countries may be driven in part by the private sector. They also suggest that preferences for caesarean delivery may be higher among highly educated women, and that individual- and provider-level factors interact in driving caesarean rates higher. Rates of caesarean section in the private sector, and their maternal and neonatal health outcomes, require close monitoring.

For peer review only

For peer review only - http://bmjopen.bmj.com/site/about/guidelines.xhtml

Article summary

Article focus

- Caesarean sections are on the rise in South Asia. Research suggests that private health facilities may be contributing to this by conducting caesareans over and above clinical need.
- Literature from other settings indicates that increases in caesarean sections are shaped by both supply and demand pressures: providers often have financial incentives to intervene surgically, and women of higher socio-economic status are also more likely to want caesareans.
- This study examined the prevalence and determinants of caesarean sections in private and charitable facilities compared with public health facilities for over 45,000 births in underserved communities of rural Nepal, rural Bangladesh, rural and urban India.

Key messages

- Caesarean sections were more common in private than in public health facilities in three of four study locations (urban India, rural Nepal, and rural Bangladesh).
- We found that highly educated women delivering in private facilities were particularly likely to deliver by caesarean in Bangladesh and urban India.
- Future research should monitor caesarean section rates in the private sector in South Asia and subsequent maternal and neonatal health outcomes

Strengths and limitations

- This study had a large sample size and focused on underserved communities, which are a priority for public health interventions in South Asia.
- Our data were not nationally representative, which limits the generalisability of our findings. Other limitations included small denominators and missing data for some variables. We did not have data on some known predictors of caesarean section, which would have enhanced the completeness of our determinants analysis.

For peer review only

For peer review only - http://bmjopen.bmj.com/site/about/guidelines.xhtml

INTRODUCTION

Access to comprehensive emergency obstetric care, including caesarean section, is key to preventing the estimated 287,000 maternal and 2.9 million neonatal deaths that occur worldwide every year.[1 2] Although debate continues about how to quantify the need for life-saving obstetric surgery, a 1985 World Health Organization (WHO) report suggested that the optimal population range for caesarean section rates is between 5% and 15%, and this endures as a reference.[3 4] Caesarean section rates are increasing worldwide, albeit unequally: a recent analysis of Demographic and Health Survey (DHS) data in 26 South Asian and sub-Saharan African countries found that rates were highest among the 'urban rich' in all countries, and lowest among the 'rural poor' in 18.[5] In all countries, fewer than 5% of mothers in the poorest wealth quintile delivered by caesarean.[6]

Caesarean sections conducted without clinical need can have adverse consequences for mothers and children. A 2008 WHO survey of 373 facilities across 24 countries found that unnecessary caesareans were associated with an increased risk of maternal mortality and serious outcomes for mothers and newborn infants, compared with spontaneous vaginal delivery.[7] Recent ecological analyses also highlighted strong associations between caesarean delivery and increased neonatal mortality in countries with low and medium caesarean section rates.[8] Unnecessary caesareans lead to considerable costs for families and health systems: an estimated 6.2 million unnecessary procedures were performed in 2008, costing approximately US$ 2.32 billion. [9]

Several South Asian countries have recorded substantial increases in caesarean section rates over the past decade. In Bangladesh, rates rose from 2% (2000) to 17% (2011); in India, from 3% (1992) to 11 % (2006); and in Nepal, from 1% (2000) to 5% (2011).[10-5] Several studies in these countries have raised concerns about high caesarean rates in private facilities, and a recent DHS analysis speculated that national increases in caesarean section rates in South Asian countries could be driven in part by higher rates among deliveries in private sector facilities[.6 16-18] Literature from other settings indicates that increases in caesarean sections are shaped by both supply and demand pressures: providers often have financial incentives to intervene surgically, and women of higher socio-economic status are also more likely to desire caesareans.[19 20]

There are few large, recent community-based studies from South Asia quantifying differences in caesarean section rates between public and private facilities. Such studies are necessary in order to examine whether increases in caesareans in these settings are indeed likely to be driven by the private sector, demand from wealthier and more educated mothers, or a combination of the two. We conducted a cross-sectional analysis of data from Bangladesh, India, and Nepal to explore the prevalence and determinants of caesarean section delivery by type of facility and maternal characteristics.

For peer review only - http://bmjopen.bmj.com/site/about/guidelines.xhtml

METHODS

Study populations

We used data collected through vital events surveillance systems established during four cluster-randomised controlled trials conducted between 2005 and 2011. The trials were done with communities that can be considered socio-economically disadvantaged: in Bangladesh and Nepal, they took place in four rural, underserved districts (Bogra, Maulvibazaar, and Faridpur in Bangladesh, and the Terai district of Dhanusha, Nepal); in rural India, most participants were from Scheduled Tribes in two states of eastern India (Jharkhand and Odisha); in urban India, data came from slum communities in Mumbai. **Table 1** describes the characteristics of each study and its population, including the background maternal mortality ratio and types of facilities present in the study areas. The original trials were designed to evaluate the impact of participatory women's groups on maternal and neonatal health outcomes.[21-25] We used data from the control areas of these trials only, because the women's group intervention led to changes in mortality and practices in several locations.[26]

Health system contexts

All three study countries have experienced substantial increases in institutional delivery rates over the last two decades: births in health facilities increased from 4% (1993) to 29% (2011) in Bangladesh; from 26% (1992-3) to 47% (2007-8) in India; and from 8% (1996) to 35% (2011) in Nepal.[10-15] All three countries have implemented incentive schemes to promote institutional delivery, though these have varying coverage. In 2010, Bangladesh's pilot maternity voucher scheme reached an estimated 10.4 million people across 31 sub-districts, around 7% of the country's population.[27 28] Mothers receive a cash incentive for antenatal care and delivery in a public or private facility, or at home with a skilled birth attendant. Both government and private facility staff also receive cash incentives, including 3000 Bangladeshi Taka (US $38.5) for a caesarean section and 300 Taka for a normal delivery.[28] This maternity incentive scheme was operational in two of the three districts (Faridpur and Maulvibazaar) covered by our study. All three districts had public facilities including District Hospitals, Maternal and Child Welfare Centres, and Upazilla Health Complexes. Private facilities included a number of small-to-medium-size clinics, BRAC (NGO) facilities, and larger private hospitals.

In India, the *Janani Suraksha Yojana* (JSY) maternity incentive scheme entitles women in rural areas of high focus states, including those in this study, to 1400 Indian Rupees ($22.4) after delivering in a government or accredited private health facility. Local community health volunteers called Accredited Social Health Activists (ASHAs) also receive 600 Indian Rupees ($9.6) for identifying pregnant women and helping them get to a health facility.[29] Although the rural Indian data included in this study were collected between 2005 and 2008, the JSY was only operational in the study areas from 2008 onwards

For peer review only - http://bmjopen.bmj.com/site/about/guidelines.xhtml

and its impact is unlikely to be reflected here. The Indian urban area included in our study spanned informal settlements (slums) with a wealth of public and private providers.[30] Mothers with Below Poverty Line cards in such areas are eligible for JSY and can receive Rs 500 towards the costs of delivering in a health facility.[31] Nepal began a safe delivery incentive scheme in 2005 and free deliveries have been available in government facilities since 2009 through the *Aama Surakshya* programme.[32] Dhanusha district, from which data for this study come, has one zonal tertiary hospital and a private medical college hospital equipped for c-sections, and a variety of small public and private health facilities without comprehensive obstetric care.

Data collection

All data were collected using surveillance systems to monitor births and deaths prospectively.[21-25] In all study locations, a female community-based key informant reported births and deaths in her area, which covered a population ranging from 250 to 350. A trained interviewer then verified these reports and paid the informant an incentive for each correct identification. In Bangladesh, rural and urban India, the interviewer administered a structured questionnaire to all eligible mothers around six weeks after delivery; in Nepal, all births in the study area were registered, and interviews were conducted on all births in small clusters and on a random sample of 10 births per month in the larger clusters. In each study location, mothers were interviewed using a questionnaire to collect information about events in the antenatal, delivery and postnatal periods. In each trial and in this subsequent study, participants were women of reproductive age (15-49) who delivered in the study area during the data collection period, and who consented to be interviewed six weeks after delivery, and their infants.

Study sample

The initial sample for this analysis included 46,393 births in Mumbai, rural India, rural Bangladesh, or rural Nepal, of whom 17,565 (38%) were delivered in health care facilities. We excluded the 2348 births occurring outside Mumbai, because we collected limited information on delivery location for these. Additionally, 223 deliveries had missing data on form of delivery (caesarean or vaginal), 602 had missing or implausible values for mother's age, and 98 were missing information on other maternal characteristics (educational attainment, household assets, number of prior pregnancies, number of antenatal visits, severe problems in pregnancy or delivery). 143 births had missing or incomplete information on place of delivery. The final analytic sample included 45,327 births (97.7% of the initial sample).

Measures used

For peer review only - http://bmjopen.bmj.com/site/about/guidelines.xhtml

The primary outcome in these analyses was caesarean section delivery, identified by self-report from the mother or another household member around six weeks after giving birth. The main covariate of interest was the type of delivery facility, coded as public (e.g. district hospitals), private (individual private clinics or hospitals), or NGO/charitable (e.g. Christian missionary hospitals in rural India; BRAC clinics in Bangladesh). We grouped private and NGO/charitable facilities together due to the small number of births in NGO/charitable facilities (6% of institutional deliveries in Bangladesh, 7% in rural India, and 0 in urban India and rural Nepal; data on request). Additional covariates in the models included measures of the characteristics of the pregnancy and delivery, maternal socio-demographic characteristics, and the location in which the delivery occurred. We created an indicator variable to identify women experiencing serious problems during pregnancy or delivery, with women reporting symptoms of pre-eclampsia (blurred vision or swelling of face and hands), symptoms of eclampsia (fits or seizures during pregnancy or delivery), hæmorrhage during delivery, or labour lasting more than 24 hours considered to have serious complications. Other characteristics of the pregnancy and delivery considered in the analysis included full utilisation of antenatal care, number of prior pregnancies, and multiple pregnancy. Full utilization of antenatal care was defined as four or more visits to antenatal care, with at least one visit to a skilled provider. Number of pregnancies was entered as an ordered categorical variable, with categories of one (first), two, three, or four or more pregnancies.

Maternal socio-demographic characteristics included in the models were: mother's age at delivery, her educational attainment, and household assets. Mother's age was entered into the model as a categorical measure in 10-year groups. Educational attainment was entered as a categorical variable using the following categories: no formal education, primary education, secondary education, or bachelor's degree or higher. To develop an asset index, we used polychoric factor analysis on data on common assets and amenities found in the mother's household, and grouped the resulting factor scores into quartiles.[33] Assets and amenities included electricity, radio or cassette player, electric fan (Bangladesh and India only), television, refrigerator (Bangladesh and India only), telephone (Bangladesh and Nepal only), generator (India only), and bicycle. All models were additionally adjusted for location (Bangladesh, India, Nepal), and year of interview in three-year groups. The data were collected in a stratified, cluster-sampled survey, and we accounted for survey design in the analysis using a fixed effect for stratum and a random effect for cluster.

Statistical analysis

We used frequencies to describe caesarean section rates by delivery location at each site. We used the Generalized Linear Latent And Mixed Models (GLLAMM) procedure in Stata 13.1, with adaptive quadrature for binary outcomes, to estimate the crude association between type of delivery facility and caesarean section.[34 35] We identified other maternal, pregnancy, and delivery characteristics potentially

For peer review only - http://bmjopen.bmj.com/site/about/guidelines.xhtml

associated with caesarean section using existing literature, especially studies resulting from the WHO multi-country surveys, and entered these in adjusted models to explore how they modified the association between type of delivery facility and caesarean section, and their individual association with caesarean section. Some South Asian and Latin American studies have detected a strong association between maternal education and caesarean delivery. [7 36 37] We fitted models including indicator variables for each group of mothers by education and type of delivery facility to explore differences in the strength of association between caesarean delivery and private facility by mother's education. To account for the sampling procedure used in rural Nepal, models were adjusted using pweights (probability of selection within cluster); these weights were rescaled to reflect the total number of institutional deliveries.

Ethical approval

The trials that provided data for this study received ethical approval from the following committees: the ethics committee of the Diabetic Association of Bangladesh (Perinatal Care Project, Bangladesh Diabetes Somity, BADAS); an independent ethics committee in Jamshedpur, Jharkhand, India; the Nepal Health Research Council; the Municipal Corporation of Greater Mumbai and the Independent Ethics Committee for Research on Human Subjects (Mumbai, India); the ethics committee of the Institute of Child Health, University College London.

RESULTS

Table 2 describes the characteristics of institutional births and caesarean deliveries by location. We analysed data for 45,327 births: 21,560 in rural Bangladesh, 8,541 in rural India, 10,236 in urban India, and 4,931 in rural Nepal. The proportion of women delivering in health facilities varied widely between locations, from 90% in urban India to 21% in rural India There were also large variations in the proportion of women delivering in private/charitable facilities rather than public facilities, with the highest (77%) in rural India and the lowest (10%) in rural Nepal. The proportion of women giving birth by caesarean section in private rather than public facilities also varied widely between settings. In Bangladesh, only 21% of women delivered in a health facility, around half of them in the private/charitable sector, but 73% of private facility births were by caesarean section. In rural Nepal, 30% of the 162 private facility births were by caesarean section. In informal settlements of Mumbai, 15% of public facility deliveries were caesareans, compared with 18% of private facility deliveries. In rural India, caesarean sections were more commonly performed in public health facilities than private or charitable facilities (15% vs. 5%). **Supplemental table 1** describes the characteristics of institutional deliveries (caesarean or non-caesarean) according to type of delivery facility (private or public), maternal socio-economic and socio-demographic characteristics, pregnancy and delivery characteristics, and year of delivery.

For peer review only - http://bmjopen.bmj.com/site/about/guidelines.xhtml

Table 3 shows crude and adjusted measures of association between type of delivery facility (private or public) and caesarean section for each location. Delivering in a private health facility was associated with an increased odds of caesarean section in all but one location (rural India). The relative odds of a caesarean in a private facility were greatest in Bangladesh (OR: 6.82, 95% CI: 5.96, 7.81), followed by Nepal (OR: 2.42, 95% CI: 1.48, 3.94) and urban India (OR 1.36, 95% CI: 1.21, 1.52). These associations persisted and were only mildly attenuated when adjusted for maternal characteristics, pregnancy and delivery characteristics, and year of delivery (Bangladesh AOR: 5.91, 95% CI: 5.15, 6.78; Nepal AOR: 2.37, 95% CI: 1.62, 3.44; urban India AOR: 1.22, 95% CI: 1.09, 1.38).

Women who had four or more antenatal check-ups were significantly more likely to have a caesarean delivery in rural Bangladesh and Nepal, with positive but non-significant trends in all other locations. Parity was not associated with caesarean delivery in any location. Having a serious health complication during pregnancy and delivery was associated with caesarean delivery in all locations except rural Bangladesh, where we observed a negative association (AOR: 0.87, 95% CI: 0.76, 1.00). Multiple birth was associated with an increased odds of caesarean section only in urban India and in rural Nepal. Higher maternal age was only associated with an increased odds of caesarean section in urban India. Maternal education was only associated with an increased odds of caesarean delivery in rural Bangladesh and urban India, with mothers with secondary education or Bachelor's degrees having higher odds (AOR: 1.44, 95% CI: 1.13, 1.84 and AOR 2.44, 95% CI: 1.52, 3.92 for Bangladesh and AOR: 1.22, 95% CI: 1.04, 1.42 and 1.62, 95% CI: 1.30, 2.02 for urban India, respectively). There were significant positive associations only in rural Bangladesh, with small increases in odds of caesarean section for unit increase in wealth quartile (AOR comparing wealthiest to poorest group in Bangladesh: 1.36, 95% CI: 1.09,1.70), and in urban India, for the wealthiest quartile (AOR: 1.50, 95% CI: 1.27, 1.78).

We found interactive associations between maternal education and private facility delivery in two of four sites, with including an interaction improving model fit in two of the four analyses (p=0.021 in rural Bangladesh and p<0.001 in urban India; **table 4 and supplemental Table 2**). In Bangladesh, there was a pronounced positive educational gradient in caesarean delivery within those who delivered in private facilities, as well as a positive association between private facility delivery and caesarean delivery. Women with bachelor's degrees delivering in private facilities had 11 times greater odds of delivering by caesarean (AOR 11.09, 95% CI: 6.28, 19.57) than did women with no formal education delivering in public facilities. Women with no formal education delivering in private facilities had nearly seven times the odds of caesarean compared with the reference group (AOR 6.94, 95% CI: 4.64, 10.39). However, the interaction between educational attainment was negative, suggesting that women holding bachelor's degrees are somewhat less likely than women with secondary degrees to deliver with caesarean

For peer review only - http://bmjopen.bmj.com/site/about/guidelines.xhtml

(**supplemental table 2**). A positive gradient was seen among women delivering in public facilities, with women with bachelor's education having four times greater odds of caesarean delivery in public facilities than women with no formal education (AOR 4.55, 95% CI: 2.22, 9.33). There was also an educational gradient among women delivering in private facilities in urban India. Educated mothers delivering in private facilities were more likely to deliver by caesarean than were mothers in other groups (AOR 2.10; 95% CI: 1.61, 2.75). However, there was no apparent gradient by educational attainment among women delivering in public facilities.

DISCUSSION

Our analysis of data from over 45,000 births confirms the findings of other studies identifying differences in caesarean rates between public and private facilities, and suggests that, even in underserved areas in South Asia, caesareans without medical indication are of concern. In three of four locations, rates of caesarean section were higher in private/charitable facilities than in public facilities. The findings from Bangladesh are particularly noteworthy as they show much greater odds of caesarean section in private facilities, concurring with previous analyses.[37] This was also the only location where serious complications in pregnancy and delivery were not associated with caesarean delivery, suggesting that obstetric surgery was performed over and above clinical need.

Our findings confirm the results of earlier studies of the prevalence of caesarean delivery in South Asia, and indicate that high rates can be found in underserved rural areas. In India, a recent analysis of 2010-11 Annual Health Survey (AHS) data from 284 districts in nine States, including Jharkhand, found that the median caesarean section rate in the private sector was 28%, compared with 5% in the public sector.[38] There appear to be strong financial incentives for surgical procedures in the private sector.[39] In a rural, largely indigenous part of eastern India where more mothers delivered in private/charitable facilities than in public facilities, more caesarean sections were performed in the public sector. In this particular setting, our field experience suggests that only women with serious complications would go to a facility and have a caesarean section, especially as JSY was not yet available in the study areas at the time of data collection. Such women are likely to have experienced multiple referrals from either ill-equipped public or private facilities not wanting to take the risk of admission. It is however possible that preference for the private sector has changed in Jharkhand since the advent of JSY, and further disaggregated analyses of Annual Health Survey data would allow a more contemporary exploration of State-level variations in caesarean section rates between public and private sectors.

In Mumbai slums with a high uptake of institutional births and a 60/40 split in favour of the public sector for delivery care, we found no difference in caesarean section rates between the two sectors, which is

For peer review only - http://bmjopen.bmj.com/site/about/guidelines.xhtml

somewhat surprising given the high rates of caesareans observed in the private sector in nine other states of India (excluding Maharashtra). At least two scenarios may be relevant in this setting. First, public sector hospitals are able to provide caesarean section, while smaller private maternity homes may not be able to. Second, the costs of caesarean section borne by private sector providers are higher in Mumbai, and families may be unwilling to bear these additional costs. This provides motivation for private providers not to provide caesareans, and makes it likely that women experiencing complications move into the public sector. The variety of facility options in an urban environment means that mothers are able to choose between different types of facilities, while in rural areas only one provider may be accessible.

Analyses using the most recent Bangladesh DHS found that, in 2011, three in five facility births were delivered by caesarean section. This reflects a historical trend: in 2001-2003, nearly half of deliveries in private facilities in Bangladesh were already by caesarean section.[18] Our data from rural, socio-economically disadvantaged communities in three districts of Bangladesh confirm these population-level findings, but also suggest that high caesarean section rates in private facilities are not merely an issue for wealthy urban mothers. Although some research from Bangladesh suggests that mothers may have a preference for caesarean delivery because of fear of labour pain or a desire to select an auspicious date for the birth, other studies also highlight women's fears of caesarean section and their distrust of health providers who recommend them, mainly because of the high costs associated with the procedure.[37 40 41] A qualitative study involving twenty women who had experienced obstetric complications in Matlab in 2008-9 found that most of the fourteen women who had undergone caesareans had spent over Tk 14,999 (US$ 217) on the procedure, which was approximately one-third of GDP per capita at the time.[41] The lack of association between caesareans and complications in pregnancy or delivery and multiple births, coupled with the high financial incentives given to providers for performing caesarean sections and the requirement for junior doctors to 'practise' their surgical skills, further suggest that obstetric surgery is being used over and above clinical need. A possible explanation for the significant interactive associations between maternal education and caesarean delivery in both public and private facilities in Bangladesh is that well-educated women may be delivering in more expensive or highly rated institutions, which may in turn be more likely to do caesarean sections for financial reasons and if they act as training centres for junior doctors.

In our sample of institutional deliveries from rural Nepal, 16% of facility births were by caesarean, which is higher than the national average: the 2011 Nepal DHS found an overall national caesarean section rate of 5%, with sections more commonly performed for births to highly educated mothers (13%) and mothers in the highest wealth quintile (14%).[26] It is possible that women with complications are more likely to deliver in facilities, and also that, as in Bangladesh, private providers are motivated by financial

For peer review only - http://bmjopen.bmj.com/site/about/guidelines.xhtml

incentives to conduct caesareans more frequently than strictly necessary. Women who can afford to seek care in private facilities may also be more willing or able to pay for caesarean sections, and providers may conduct more of them to increase their income.

A 2010 analysis of DHS data examining the role of the private sector in maternity care in 16 countries found evidence of a trend towards privatisation in delivery care between the 1990s and mid-2000s, but with strong differences between countries, which might reflect the heterogeneous nature of this sector both between and within countries. [42] This DHS study highlighted the need for more context-specific data on the nature of the private sector in low and middle-income countries, and its role in maternity care. Further research might focus on understanding the motivations and experiences of women undergoing caesarean sections in private facilities in South Asian settings, pathways for switching between public and private sectors in the event of obstetric complications, a more comprehensive tally of the financial incentives (official or non-official) that motivate private providers to carry out caesareans in each setting, and the consequences of increased caesarean sections in the private sector for maternal and neonatal health outcomes.

In our study, locations with higher prevalence of caesarean deliveries also showed a positive educational gradient even after adjusting for wealth measured by household assets. Moreover, there was an interactive association between education and type of facility, with highly educated women particularly likely to receive caesarean deliveries in private facilities in urban India. Literature exploring the determinants of caesarean delivery emphasizes that multiple influences drive a woman's decision to deliver by caesarean. Profit considerations at the facility level may prompt some providers to urge women to receive unnecessary procedures, while women themselves may prefer caesareans for cultural reasons, fear of painful deliveries, or because they believe it to be safer. [36 43-45] Within the client-provider interaction, providers may be more likely to acquiesce to a request for caesarean from a highly educated woman; and such women may be more likely to accept advice from a provider. [19 46] Our findings lend support to the hypothesis that, while provider-level factors are probably partially responsible for the rapid increase in caesarean deliveries, it is also necessary to consider women's own preferences and decision-making processes and how they are shaped by social and cultural factors.

Strengths and limitations

The strengths of the study were its large sample size and focus on underserved communities, which are a priority for public health interventions in South Asia. It had five main limitations. It was not a nationally representative study, and districts or clusters were sampled purposively from previous cRCTs. This limits the generalisability of our findings to geographical settings outside the study areas. The study was cross-sectional, and therefore only able to suggest associations rather than causal relationships. The data also

For peer review only - http://bmjopen.bmj.com/site/about/guidelines.xhtml

did not include key predictors of caesarean section such as breech presentation, and whether the previous delivery was by caesarean, which limits the completeness of our analysis of determinants. In addition, levels of serious complications in pregnancy and delivery varied considerably between locations and were often higher than expected, suggesting potential over-reporting and limited reliability as an indicator of complications. Finally, some variables had small denominators, and others had high levels of missing data (for example, maternal age in rural India).

Conclusions

Our study found that delivering in a private health facility was associated with an increased odds of caesarean section in three of four South Asian locations, and that the associations persisted after adjustments for maternal, pregnancy and delivery characteristics, and year of delivery. We also found significant interactive associations between maternal education and caesarean delivery in Bangladesh (both private and public facilities) and urban India (private facilities only). These results lend support to the hypothesis that increased caesarean section rates in these three South Asian countries may in part be driven by the private sector, but also suggest that, in some settings such as Bangladesh and urban India, demand from more educated mothers may play a part. These findings call for greater, local understanding of the role of private providers in maternity care, together with careful examination of the consequences of increased caesarean sections in the private sector for maternal and neonatal health outcomes.

Competing interests

None to declare

Contributors

MN and AP had the original idea for the study. MN designed the data analysis strategy in discussion with AP. MN, GA, AS, CS and NS prepared the data. MN performed the analysis and prepared the tables. NN, PT, NSM, DO, KA, DM and AC are responsible for the trials. AP wrote the first draft of the paper and all authors read and commented on the draft. All authors read and approved the final manuscript.

Data sharing

Data used in the analyses for this study are available upon request, subject to completion of a data sharing agreement. Please contact audrey.prost@ucl.ac.uk for further information.

For peer review only - http://bmjopen.bmj.com/site/about/guidelines.xhtml

Funding

MN and NS are funded through a Wellcome Trust Strategic Award (085417MA/Z/08/Z). In Bangladesh, the Perinatal Care Project trial (covering KA and AK) was registered with trial number ISRCTN54792066 and has received funding from the Big Lottery Fund and the Wellcome Trust. In Eastern India, the Ekjut trial (covering NN and PT) was registered with trial number ISRCTN21817853 and funded by the Health Foundation. The Dhanusha (Nepal) trial (covering DSM, NS, BPS, and CS) was registered with trial number ISRCTN87820538 and has received funding from: the UBS Optimus Foundation; USAID; DfID and the Wellcome Trust. The City Initiative for Newborn Health trial led by SNEHA (Mumbai, India) was registered with trial number ISRCTN96256793 and has received funding from the ICICI Foundation for Inclusive Growth and the Wellcome Trust. GA, NSM, and DO are currently funded by The Wellcome Trust (091561/Z/10/Z). The funders played no part in the development of the article or the decision to submit it for publication.

Provenance and peer review

Not commissioned; externally peer reviewed.

REFERENCES:

1. World Health Organisation and UNICEF. Countdown 2015: Building a future for women and Children, the 2012 Report. http://countdown2015mnch.org/documents/2012Report/2012-Complete.pdf [Accessed 9th November 2013]
2. Campbell OM, Graham WJ. Strategies for reducing maternal mortality: getting on with what works. *The Lancet* 2006; 368: 1284-1299.
3. Ronsmans C, De Brouwere V, Dubourg D, *et al*.. Measuring the need for life-saving obstetric surgery in developing countries. *BJOG* 2004; 111: 1027-30.
4. World Health Organization. Appropriate technology for birth. *The Lancet* 1985; 2: 436-7.
5. Betrán AP, Merialdi M, Lauer JA, *et al*. Rates of caesarean section: analysis of global, regional and national estimates. *Paediatric and Perinatal Epidemiology* 2007; 21: 98-113.
6. Cavallaro FL, Cresswell JA, França GVA, *et al*. Trends in Caesarean delivery by country and wealth quintile: cross-sectional surveys in southern Asia and sub-Saharan Africa. *WHO Bulletin* 2013; 91: 897-972.
7. Souza JP, Gülmezoglu A, Lumbiganon P, *et al*. WHO Global Survey on Maternal and Perinatal Health Research Group. Caesarean section without medical indications is associated with an increased risk of adverse short-term maternal outcomes: the 2004–2008 WHO Global Survey on Maternal and Perinatal Health. *BMC Medicine* 2010; 8:71.
8. Kyu HH, Shannon HS, Georgiades K, Boyle MH. Caesarian delivery and neonatal mortality rates in

For peer review only - http://bmjopen.bmj.com/site/about/guidelines.xhtml

46 low- and middle-income countries: a propensity score matching and meta-analysis of demographic health survey data. *International Journal of Epidemiology* 2013; 1-11.

9. Gibbons L, Belizán JM, Lauer JA, *et al*. The global numbers and costs of additionally needed and unnecessary caesarean sections performed per year: overuse as a barrier to universal coverage. World Health Report (2010), Background paper. Geneva: World Health Organization.
10. Mitra SN, Nawab Ali M, Islam S, *et al*. Bangladesh Demographic and Health Survey, 1993-1994. Calverton, Maryland: National Institute of Population Research and Training (NIPORT), Mitra and Associates, and Macro International Inc, 1994.
11. National Institute of Population Research and Training (NIPORT), Mitra and Associates, and ICF International. Bangladesh Demographic and Health Survey 2011. Dhaka, Bangladesh and Calverton, Maryland, USA: NIPORT, Mitra and Associates, and ICF International, 2013.
12. International Institute for Population Sciences (IIPS) and Macro International. National Family Health Survey (NFHS 1), 1992–93. India, Mumbai: IIPS, 1995.
13. International Institute for Population Sciences (IIPS), Macro International. National Family Health Survey (NFHS 3), 2005–06; India: Volume I. Mumbai: IIPS, 2007.
14. Pradhan A, Aryal RH, Regmi G, *et al*. Nepal Family Health Survey 1996. Kathmandu, Nepal and Calverton, Maryland: Ministry of Health [Nepal], New ERA, and Macro International Inc, 1997.
15. Ministry of Health and Population (MOHP) [Nepal], New ERA, and ICF International Inc. Nepal Demographic and Health Survey 2011. Kathmandu, Nepal: Ministry of Health and Population, New ERA, and ICF International, Calverton, Maryland, 2012.
16. Ajeet S, Nandkishore K. The boom in unnecessary caesarean surgeries is jeopardizing women's health. *Health Care Women International* 2013; 34: 513-521.
17. Sreevidya S, Sathiyasekaran BW. High caesarean rates in Madras (India): a population-based cross-sectional study. *BJOG* 2003; 110: 106-111.
18. Koblinksi M, Anwar I, Mridha MK, *et al*. Reducing maternal mortality and improving maternal health: Bangladesh and MDG 5. *J Health Popul Nutr* 2008; 26: 280-294.
19. Leone T, Padmadas SS, Matthews Z. Community factors influencing rising caesarean section rates in developing countries: an analysis of six countries. *Soc Sci Med* 2008; 67: 1236-46.
20. Mazzoni A, Althabe A, Liu NH, *et al*. Women's preference for caesarean section: a systematic review and meta-analysis of observational studies. *BJOG* 2011; 118: 391-399.
21. Tripathy P, Nair N, Barnett S, et al. Effect of a participatory intervention with women's groups on birth outcomes and maternal depression in Jharkhand and Orissa, India: A cluster-randomised controlled trial. *Lancet* 2010; 375: 1182–1192.
22. Azad K, Barnett S, Banerjee B, *et al*. Effect of scaling up women's groups on birth outcomes in three rural districts in Bangladesh: a cluster-randomised controlled trial. *Lancet* 2010; 375: 1193–1202.

For peer review only

23. Fottrell E, Azad K, Kuddus A, *et al*. The effect of increased coverage of participatory women's groups on neonatal mortality in Bangladesh: a cluster-randomised trial. *JAMA Pediatrics* 2013; 167: 816-825.

24. Shah More N, Bapat U, Das S, *et al*. Cluster-randomised controlled trial of community mobilisation in Mumbai slums to improve care during pregnancy, delivery, postpartum and for the newborn. *Trials* 2008; 9:7.

25. Shrestha BP, Bhandari B, Manandhar DS, *et al*. Community interventions to reduce child mortality in Dhanusha, Nepal: study protocol for a cluster randomized controlled trial. *Trials* 2011; 12: 136.

26. Prost A, Colbourn T, Seward N, *et al*. Women's groups practising participatory learning and action to improve maternal and newborn health in low-resource settings: a systematic review and meta-analysis. *Lancet* 2013; 381: 1736–46.

27. Schmidt J-0, Ensor T, Hossain A, Khan S. Vouchers as demand side financing instruments for health care: A review of the Bangladesh maternal voucher scheme. *Health Policy* 2010; 96: 98–107.

28. Ahmed S, Khan MM. A maternal health voucher scheme: what have we learned from the demand-side financing scheme in Bangladesh? *Health Policy & Planning* 2011; 6:25–32.

29. Lim SS, Dandona L, Hoisington JA, *et al*. India's Janani Suraksha Yojana, a conditional cash transfer programme to increase births in health facilities: an impact evaluation. *Lancet* 2010; 375: 209-23.

30. Shah More N, Alcock G, Bapat U, *et al*. Tracing pathways from antenatal to delivery care for women in Mumbai, India: cross-sectional study of maternity in low-income areas. International Health 2009; 1: 71–77.

31. Government of India, Ministry of Health & Family Welfare. Janani Suraksha Yojana: Guidelines for Implementation, 2006. http://www.mohfw.nic.in/NRHM/./guidelines/JSY_guidelines_09_06.pdf

32. Witter S, Khadka S, Nath H, Tiwari S. The national free delivery policy in Nepal: early evidence of its effects on health facilities. *Health Policy and Planning* 2011; 26: ii84–ii91.

33. Holgado-Tello FP,Chacón–Moscoso S, Barbero-Garcia I, Vila-Abad E. Polychoric versus Pearson correlations in exploratory and confirmatory factor analysis of ordinal variables. *Quality & Quantity* 2010; 44 (1): 153-166.

34. Rabe-Hesketh S, Skrondal A, Pickles A. Maximum likelihood estimation of limited and discrete dependent variable models with nested random effects. *Journal of Econometrics* 2005; 128: 301-323.

35. StataCorp. 2013. *Stata Statistical Software: Release 13*. College Station, TX: StataCorp LP.

36. Potter J, Berquó E, Perpétuo I, *et al*. Unwanted Caesarean Sections among Public and Private

For peer review only - http://bmjopen.bmj.com/site/about/guidelines.xhtml

Patients in Brazil: Prospective Study. *BMJ* 2001; 323: 1155-8.

37. Kamal SMM. Preference for institutional delivery and caesarean sections in Bangladesh. *J Health Popul Nutr* 2013; 31: 96-109.
38. Randive B, Diwan V, De Costa A. India's Conditional Cash Transfer Programme (the JSY) to Promote Institutional Birth: Is There an Association between Institutional Birth Proportion and Maternal Mortality? *PLoS ONE* 2013; 8(6): e67452. doi:10.1371/journal.pone.0067452
39. Chatterjee S, Laxminarayan R. Costs of surgical procedures in Indian hospitals. *BMJ Open* 2013; 3: e002844.
40. Parkhurst JO, Rahman SA. Life saving or money wasting? Perceptions of caesarean sections among users of services in Rural Bangladesh. *Health Policy* 2007; 80: 392-401.
41. Khan R, Blum LS, Sultana M, et al. An Examination of Women Experiencing Obstetric Complications Requiring Emergency Care: Perceptions and Sociocultural Consequences of Caesarean Sections in Bangladesh. *J Health Popul Nutr 2012*; 30: 159-171.
42. Pomeroy A, Koblinksy M, Alva S. Private Delivery Care in Developing Countries: Trends and Determinants. DHS Working document 2010; No.76. http://dhsprogram.com/pubs/pdf/WP76/WP76.pdf [Accessed 3rd March 2014].
43. Sufang G, Padmadas SS, Fengmin Z et al. Delivery settings and caesarean section rates in China. *Bull WHO* 2007; 85: 733-820.
44. Lo JC. Patients' attitudes vs. physicians' determination: implications for cesarean sections. *Soc Sci Med* 2003; 57: 91-6.
45. McCourt C, Weaver J, Statham H, *et al.* Elective cesarean section and decision making: a critical review of the literature. *Birth* 2007; 34: 65-79.
46. Ghetti C, Chan BKS, Guise J-M. Physicians' responses to patient-requested caesarean delivery. *Birth* 2004; 31: 280-4.

For peer review only

For peer review only - http://bmjopen.bmj.com/site/about/guidelines.xhtml

Table 1: Characteristics of studies and populations

Study (Country)	Bangladesh (rural)	India (rural)	Nepal (rural)	India (urban)
**Location**	Three districts: Bogra, Maulvibazaar and Faridpur	Three districts of Jharkhand and Odisha: Keonjhar, West Singhbhum and Saraikela	Dhanusha district (Terai)	Mumbai slums
**Period**	2005 – 2011	2005-2008	2008-2011	2006-2009
**Estimated population**	532,900	114,000	240,000	283,000
**Cluster characteristics**	Villages making up a union	8-10 villages with residents classified as Scheduled Tribe or Other Backward Class	Village Development Committee	Slum areas in six municipal wards of Mumbai
**Method of cluster identification**	Purposive sampling of three districts and clusters within districts	Purposive sampling of three districts and clusters within districts	Random sampling of 60 clusters from a list of 79 suitable clusters in one district	92 clusters in six municipal wards identified using municipal documents, surveys, discussions with key informants, and site visits. Random selection of 48 clusters for randomized allocation.
**Clusters, n**	9	18	30	24
**Cluster and individual follow-up**	All clusters followed up<br>Interviews completed after 82% of identified births in control areas in Phase 1, and 99% of births in Phase 2	All clusters followed up<br>Interviews completed after 98% of identified births	All clusters followed up	All clusters followed up<br>Interviews completed after 83% of identified births
**Maternal mortality ratio**	254.3	668.1	Unknown	206.2
**Health facilities available in control areas**	Public facilities: District Hospitals; Maternal and Child Welfare Centres; Upazilla Health Complexes. Private facilities: small-to-medium-size clinics; BRAC (NGO) facilities where deliveries do not take place; larger private hospitals with and without Comprehensive emergency obstetric care (CEmOC) facilities.	Public facilities: District Hospitals; Primary Health Centres (PHCs) in which deliveries can notionally take place but that are not usually equipped for CEmOC; Community Health Centres (CHCs) acting as referral centres for PHCs, covering a population of around 80,000 with EmOC facilities; district hospitals. Private and charitable facilities: medium-sized missionary hospitals with EmOC facilities.	Public facilities: three Primary Health Care Centres, three Health Posts and 24 Sub-Health Posts, none of which are equipped for CEmOC. These health facilities refer to the public Zonal Tertiary Hospital and various private providers in the district headquarters and nearby medical college, which have facilities for caesarean sections.	Public facilities: Municipal tertiary hospitals, general hospitals, and maternity homes. Private facilities: specialty hospitals, general hospitals, and maternity homes.

For peer review only - http://bmjopen.bmj.com/site/about/guidelines.xhtml

Table 2: Characteristics of institutional births and caesarean deliveries by location

	Births	Institutional births						Caesarean section births			
		All		Public facility		Private/charitable facility		Public facility		Private/charitable facility	
	(n)	(n)	(% all births)	(n)	(% institutional births)	(n)	(% institutional births)	(n)	(% deliveries in public facilities)	(n)	(% deliveries in private facilities)
**Bangladesh (rural)**	21,560	4,592	21%	2,053	45%	2,539	55%	589	29%	1,852	73%
**India (rural)**	8,541	1,816	21%	421	23%	1,395	77%	64	15%	66	5%
**India (urban)**	10,236	9,259	90%	5,489	59%	3,770	41%	811	15%	697	18%
**Nepal (rural)**	4,931	1,586	32%	1,424	90%	162	10%	201	14%	48	30%

* Nepal numbers weighted using women's probability of selection within each cluster, scaled to total number of institutional births. (Unweighted Nepal totals: 4,990 births and 1,586 institutional births)

For peer review only

Table 3: Mutually adjusted associations of type of facility of delivery and other selected determinants with caesarean section delivery, by location

	Bangladesh (rural) ORs (95% CIs)		India (rural) ORs (95% CIs)		India (urban) ORs (95% CIs)		Nepal (rural)* ORs (95% CIs)	
	Crude	Adjusted	Crude	Adjusted	Crude	Adjusted	Crude	Adjusted
**Health facility characteristics**								
Public health facility (ref)								
Private health facility	6.82 (5.96,7.81)	5.91 (5.15,6.78)	0.39 (0.25,0.61)	0.46 (0.29,0.73)	1.36 (1.21,1.52)	1.22 (1.09,1.38)	2.42 (1.48,3.94)	2.37 (1.62,3.44)
**Pregnancy and delivery characteristics**								
4+ antenatal care visits (fewer or none = ref)**		1.46 (1.26,1.69)		1.49 (0.96,2.32)		1.06 (0.93,1.21)		1.92 (1.43,2.58)
Birth order								
1 (ref)								
2		0.95 (0.80,1.13)		1.25 (0.80,1.95)		0.65 (0.56,0.75)		1.41 (1.02,1.94)
3		1.19 (0.94,1.52)		0.36 (0.15,0.87)		0.60 (0.50,0.71)		0.58 (0.37,0.92)
4+		1.17 (0.90,1.53)		0.36 (0.15,0.85)		0.39 (0.32,0.48)		1.00 (0.54,1.84)
Serious complications in pregnancy/delivery*** (no complications=ref)		0.87 (0.76,1.00)		1.77 (1.17,2.67)		1.71 (1.39,2.11)		4.87 (2.51,9.47)
Multiple birth (ref=single)		0.93 (0.65,1.32)		1.57 (0.52,4.75)		3.01 (2.14,4.23)		3.42 (1.77,6.61)
**Maternal characteristics**								
Age (years)								
15-24 (ref)								
25-34		1.12 (0.94,1.34)		1.81 (1.14,2.86)		1.45 (1.27,1.66)		1.52 (0.63,1.94)
35+		1.10 (0.77,1.58)		2.11 (0.63,7.07)		1.79 (1.27,2.53)		1.55 (0.55,1.47)

For peer review only

For peer review only - http://bmjopen.bmj.com/site/about/guidelines.xhtml

For peer review only

	Bangladesh (rural) ORs (95% CIs)		India (rural) ORs (95% CIs)		India (urban) ORs (95% CIs)		Nepal (rural)* ORs (95% CIs)	
	Crude	Adjusted	Crude	Adjusted	Crude	Adjusted	Crude	Adjusted
Maternal education****								
No formal education (ref)								
Primary education		1.07 (0.83,1.38)		0.98 (0.39,2.47)		1.13 (0.85,1.49)		0.90 (0.59,1.39)
Secondary education		1.44 (1.13,1.84)		1.11 (0.68,1.80)		1.22 (1.04,1.42)		1.37 (0.97,1.94)
Bachelor's degree or higher		2.44 (1.52,3.92)		1.20 (0.43,3.31)		1.62 (1.30,2.02)		- -
Household wealth quintile								
1st (poorest, ref)								
2nd		1.31 (1.06,1.63)		1.61 (0.69,3.75)		1.14 (0.77,1.68)		1.11 (0.63,1.94)
3rd		1.41 (1.10,1.82)		1.36 (0.58,3.21)		1.13 (0.95,1.33)		0.90 (0.55,1.47)
4th (wealthiest)		1.36 (1.09,1.70)		2.16 (0.87,5.33)		1.50 (1.27,1.78)		0.99 (0.52,1.90)
Year of delivery								
2004-2006 (ref: BD, IN)								-
2007-2009 (ref: NP rural)		0.90 (0.74,1.10)		0.90 (0.61,1.33)		1.14 (1.09,1.38)		- -
2010-2012		1.15 (0.94,1.41)		- -		- -		1.29 (0.91,1.82)
N	4,592	4,592	1,816	1,816	9,259	9,259	1,586	1,586

* Nepal numbers weighted using women's probability of selection within each cluster, scaled to total number of institutional births.

** At least one visit with skilled provider

***Includes: symptoms of eclampsia (fits, seizures, convulsions, or unconsciousness during pregnancy or delivery); reduced or no fetal movement; labour lasting more than 24 hours.

****For the Nepal data, two respondents with bachelor's degrees (2 respondent total, 1 delivering in institution) were combined with respondents with secondary education.

All analyses additionally adjusted for survey design using fixed effect of stratum and random effect of cluster.

Table 4: Interactive associations between type of delivery facility, maternal education, and caesarean delivery, by location

	Bangladesh (rural)		India (rural)		India (urban)		Nepal (rural)*	
	(n)	AOR, 95% CI	(n)	AOR, 95% CI	(n)	AOR, 95% CI	(unweighted n)	AOR, 95% CI
Public facility - no maternal education (ref)	202	-	109	-	1,396	-	822	-
Public facility - primary education	448	1.07 (0.76,1.50)	17	0.92 (0.18,4.77)	357	0.88 (0.61,1.27)	196	1.04 (0.65,1.65)
Public facility - secondary education	202	1.63 (1.18,2.24)	604	1.09 (0.52,2.26)	920	1.1 (0.91,1.33)	413	1.45 (1.04,2.02)
Public facility - bachelor's degree	448	4.58 (2.24,9.38)	100	1.55 (0.41,5.89)	180	1.06 (0.76,1.47)	-	- -
Private facility - no education	332	7.24 (4.82,10.87)	109	0.46 (0.21,1.01)	1396	0.91 (0.70,1.19)	100	2.83 (1.73,4.61)
Private facility - primary education	578	7.58 (5.36,10.71)	17	0.47 (0.14,1.58)	357	1.52 (0.99,2.33)	16	0.86 (0.14,5.21)
Private facility - secondary education	1,102	9.03 (6.57,12.41)	274	0.52 (0.24,1.12)	3,382	1.3 (1.06,1.60)	39	3.01 (1.71,5.30)
Private facility - bachelor's degree	41	11.24 (6.37,19.85)	21	0.4 (0.08,2.00)	354	2.1 (1.61,2.75)	-	- -
N	3,353		1,251		8,342		1586	
p-value**	0.021		0.792		0.001		0.394	

All results adjusted for number of antenatal care visits, parity, medical indication for caesarean, multiple birth, maternal age (10-yr group), household assets, stratum, and cluster (random effect).
* Nepal numbers weighted using women's probability of selection within each cluster, scaled to total number of institutional births.

** p-value from -2 log-likelihood test comparing nested models with and without interaction terms

For peer review only

Prevalence and determinants of caesarean section in private and public health facilities in underserved South Asian communities: cross-sectional analysis of data from Bangladesh, India, and Nepal

Melissa Neuman[1], Glyn Alcock[1], Kishwar Azad[4], Abdul Kuddus[4], David Osrin[1], Neena Shah More[3], Nirmala Nair[5], Prasanta Tripathy[5], Catherine Sikorski[1], Naomi Saville[1], Aman Sen[2], Tim Colbourn[1], Tanja AJ Houweling[6], Nadine Seward[1], Dharma S Manandhar[2], Bhim P Shrestha[2], Anthony Costello[1] and Audrey Prost[1]

Author affiliations:

[1] Institute for Global Health, University College London, 30 Guilford Street, London WC1N 1EH, UK

[2] Mother and Infant Research Activities (MIRA), YB Bhavan, Thapathali, GPO Box 921, Kathmandu, Nepal

[3] Society for Nutrition, Education and Health Action (SNEHA), Urban Health Centre, Shahunagar, Dharavi, Mumbai 400017, Maharashtra, India

[4] Perinatal Care Project, Diabetic Association of Bangladesh, 122 Kazi Nazrul Islam Avenue, Dhaka 1000, Bangladesh

[5] Ekjut, Plot 556B, Potka, Chakradharpur, West Singhbhum, Jharkhand, India

[6] Department of Public Health, Erasmus MC University Medical Center Rotterdam, P.O. Box 2040, 3000 CA Rotterdam, The Netherlands

Proposed running head: Caesarean sections in private vs. public health facilities in South Asia

Word count: 4393

Figures and tables: 0 figures and 4 tables

For peer review only - http://bmjopen.bmj.com/site/about/guidelines.xhtml

ABSTRACT (Max: 300 words; Current: 300)

Objectives: To describe the prevalence and determinants of births by caesarean section in private and public health facilities in underserved communities in South Asia.

Design: Cross-sectional study.

Setting: 81 community-based geographical clusters in four locations in Bangladesh, India and Nepal (three rural, one urban).

Participants: 45,327 births occurring in the study areas between 2005 and 2012.

Outcome measures: proportion of caesarean section delivery by location and type of facility; determinants of caesarean section delivery by location.

Results: Institutional delivery rates varied widely between settings, from 21% in rural India to 90% in urban India. The proportion of private and charitable facility births delivered by caesarean section was 73% in Bangladesh, 30% in rural Nepal, 18% in urban India, and 5% in rural India. The odds of caesarean section were greater in private and charitable health facilities than in public facilities in three of four study locations, even when adjusted for pregnancy and delivery characteristics, maternal characteristics, and year of delivery (Bangladesh AOR: 5.91, 95% CI: 5.15-6.78; Nepal AOR: 2.37, 95% CI: 1.62-3.44; urban India AOR: 1.22, 95% CI: 1.09-1.38). We found that ~~significant interactive associations between maternal education and private facility delivery in two of four locations, with~~ highly educated women were particularly likely to deliver by caesarean in private facilities in both urban India (AOR 2.10; 95% CI: 1.61, 2.75) and rural Bangladesh (AOR 11.09, 95% CI: 6.28, 19.57).

Conclusions:

Our results lend support to the hypothesis that increased caesarean section rates in these South Asian countries may be driven in part by the private sector. They also suggest that preferences for caesarean delivery may be higher among highly educated women, and that individual- and provider-level factors interact in driving caesarean rates higher. Rates of caesarean section in the private sector, and their maternal and neonatal health outcomes, require close monitoring.

For peer review only

Article summary

Article focus

- Caesarean sections are on the rise in South Asia. Research suggests that private health facilities may be contributing to this by conducting caesareans over and above clinical need.
- Literature from other settings indicates that increases in caesarean sections are shaped by both supply and demand pressures: providers often have financial incentives to intervene surgically, and women of higher socio-economic status are also more likely to want caesareans.
- This study examined the prevalence and determinants of caesarean sections in private and charitable facilities compared with public health facilities for over 45,000 births in underserved communities of rural Nepal, rural Bangladesh, rural and urban India.

Key messages

- Caesarean sections were more common in private than in public health facilities in three of four study locations (urban India, rural Nepal, and rural Bangladesh).
- We ~~found significant interactive associations between~~ found that highly educated women ~~maternal education and~~ delivering in private facilitiesy ~~delivery on~~ were particularly likely to deliver by caesarean in Bangladesh ~~(both private and public facilities)~~ and urban India ~~(private facilities only)~~.
- Future research should monitor caesarean section rates in the private sector in South Asia and subsequent maternal and neonatal health outcomes

Strengths and limitations

- This study had a large sample size and focused on underserved communities, which are a priority for public health interventions in South Asia.
- Our data were not nationally representative, which limits the generalisability of our findings. Other limitations included small denominators and missing data for some variables. We did not have data on some known predictors of caesarean section, which would have enhanced the completeness of our determinants analysis.

For peer review only - http://bmjopen.bmj.com/site/about/guidelines.xhtml

INTRODUCTION

Access to comprehensive emergency obstetric care, including caesarean section, is key to preventing the estimated 287,000 maternal and 2.9 million neonatal deaths that occur worldwide every year.[1 2] Although debate continues about how to quantify the need for life-saving obstetric surgery, a 1985 World Health Organization (WHO) report suggested that the optimal population range for caesarean section rates is between 5% and 15%, and this endures as a reference.[3 4] Caesarean section rates are increasing worldwide, albeit unequally: a recent analysis of Demographic and Health Survey (DHS) data in 26 South Asian and sub-Saharan African countries found that rates were highest among the 'urban rich' in all countries, and lowest among the 'rural poor' in 18.[5] In all countries, fewer than 5% of mothers in the poorest wealth quintile delivered by caesarean.[6]

Caesarean sections conducted without clinical need can have adverse consequences for mothers and children. A 2008 WHO survey of 373 facilities across 24 countries found that unnecessary caesareans were associated with an increased risk of maternal mortality and serious outcomes for mothers and newborn infants, compared with spontaneous vaginal delivery.[7] Recent ecological analyses also highlighted strong associations between caesarean delivery and increased neonatal mortality in countries with low and medium caesarean section rates.[8] Unnecessary caesareans lead to considerable costs for families and health systems: an estimated 6.2 million unnecessary procedures were performed in 2008, costing approximately US$ 2.32 billion. [9]

Several South Asian countries have recorded substantial increases in caesarean section rates over the past decade. In Bangladesh, rates rose from 2% (2000) to 17% (2011); in India, from 3% (1992) to 11 % (2006); and in Nepal, from 1% (2000) to 5% (2011).[10-5] Several studies in these countries have raised concerns about high caesarean rates in private facilities, and a recent DHS analysis speculated that national increases in caesarean section rates in South Asian countries could be driven in part by higher rates among deliveries in private sector facilities[.6 16-18] Literature from other settings indicates that increases in caesarean sections are shaped by both supply and demand pressures: providers often have financial incentives to intervene surgically, and women of higher socio-economic status are also more likely to desire caesareans.[19 20]

There are few large, recent community-based studies from South Asia quantifying differences in caesarean section rates between public and private facilities. Such studies are necessary in order to examine whether increases in caesareans in these settings are indeed likely to be driven by the private sector, demand from wealthier and more educated mothers, or a combination of the two. We conducted a cross-sectional analysis of data from Bangladesh, India, and Nepal to explore the prevalence and determinants of caesarean section delivery by type of facility and maternal characteristics. ~~location,~~

For peer review only - http://bmjopen.bmj.com/site/about/guidelines.xhtml

and to examine the interactions between maternal education and place of delivery on prevalence of caesarean section.

METHODS

Study populations

We used data collected through vital events surveillance systems established during four cluster-randomised controlled trials conducted between 2005 and 2011. The trials were done with communities that can be considered socio-economically disadvantaged: in Bangladesh and Nepal, they took place in four rural, underserved districts (Bogra, Maulvibazaar, and Faridpur in Bangladesh, and the Terai district of Dhanusha, Nepal); in rural India, most participants were from Scheduled Tribes in two states of eastern India (Jharkhand and Odisha); in urban India, data came from slum communities in Mumbai. **Table 1** describes the characteristics of each study and its population, including the background maternal mortality ratio and types of facilities present in the study areas. The original trials were designed to evaluate the impact of participatory women's groups on maternal and neonatal health outcomes.[21-25] We used data from the control areas of these trials only, because the women's group intervention led to changes in mortality and practices in several locations.[26]

Health system contexts

All three study countries have experienced substantial increases in institutional delivery rates over the last two decades: births in health facilities increased from 4% (1993) to 29% (2011) in Bangladesh; from 26% (1992-3) to 47% (2007-8) in India; and from 8% (1996) to 35% (2011) in Nepal.[10-15] All three countries have implemented incentive schemes to promote institutional delivery, though these have varying coverage. In 2010, Bangladesh's pilot maternity voucher scheme reached an estimated 10.4 million people across 31 sub-districts, around 7% of the country's population.[27 28] Mothers receive a cash incentive for antenatal care and delivery in a public or private facility, or at home with a skilled birth attendant. Both government and private facility staff also receive cash incentives, including 3000 Bangladeshi Taka (US $38.5) for a caesarean section and 300 Taka for a normal delivery.[28] This maternity incentive scheme was operational in two of the three districts (Faridpur and Maulvibazaar) covered by our study. All three districts had public facilities including District Hospitals, Maternal and Child Welfare Centres, and Upazilla Health Complexes. Private facilities included a number of small-to-medium-size clinics, BRAC (NGO) facilities, and larger private hospitals.

In India, the *Janani Suraksha Yojana* (JSY) maternity incentive scheme entitles women in rural areas of high focus states, including those in this study, to 1400 Indian Rupees ($22.4) after delivering in a government or accredited private health facility. Local community health volunteers called Accredited Social Health Activists (ASHAs) also receive 600 Indian Rupees ($9.6) for identifying pregnant women and

For peer review only

helping them get to a health facility.[29] Although the rural Indian data included in this study were collected between 2005 and 2008, the JSY was only operational in the study areas from 2008 onwards and its impact is unlikely to be reflected here. The Indian urban area included in our study spanned informal settlements (slums) with a wealth of public and private providers.[30] Mothers with Below Poverty Line cards in such areas are eligible for JSY and can receive Rs 500 towards the costs of delivering in a health facility.[31] Nepal began a safe delivery incentive scheme in 2005 and free deliveries have been available in government facilities since 2009 through the *Aama Surakshya* programme.[32] Dhanusha district, from which data for this study come, has one zonal tertiary hospital and a private medical college hospital equipped for c-sections, and a variety of small public and private health facilities without comprehensive obstetric care.

Data collection

All data were collected using surveillance systems to monitor births and deaths prospectively.[21-25] In all study locations, a female community-based key informant reported births and deaths in her area, which covered a population ranging from 250 to 350. A trained interviewer then verified these reports and paid the informant an incentive for each correct identification. In Bangladesh, rural and urban India, the interviewer administered a structured questionnaire to all eligible mothers around six weeks after delivery; in Nepal, all births in the study area were registered, and interviews were conducted on all births in small clusters and on a random sample of 10 births per month in the larger clusters. In each study location, mothers were interviewed using a questionnaire to collect information about events in the antenatal, delivery and postnatal periods. In each trial and in this subsequent study, participants were women of reproductive age (15-49) who delivered in the study area during the data collection period, and who consented to be interviewed six weeks after delivery, and their infants.

Study sample

The initial sample for this analysis included 46,393 births in Mumbai, rural India, rural Bangladesh, or rural Nepal, of whom 17,565 (38%) were delivered in health care facilities. We excluded the 2348 births occurring outside Mumbai, because we collected limited information on delivery location for these. Additionally, 223 deliveries had missing data on form of delivery (caesarean or vaginal), 602 had missing or implausible values for mother's age, and 98 were missing information on other maternal characteristics (educational attainment, household assets, number of prior pregnancies, number of antenatal visits, severe problems in pregnancy or delivery). 143 births had missing or incomplete information on place of delivery. The final analytic sample included 45,327 births (97.7% of the initial sample).

For peer review only

Measures used

The primary outcome in these analyses was caesarean section delivery, identified by self-report from the mother or another household member around six weeks after giving birth. The main covariate of interest was the type of delivery facility, coded as public (e.g. district hospitals), private (individual private clinics or hospitals), or NGO/charitable (e.g. Christian missionary hospitals in rural India; BRAC clinics in Bangladesh). We grouped private and NGO/charitable facilities together due to the small number of births in NGO/charitable facilities (6% of institutional deliveries in Bangladesh, 7% in rural India, and 0 in urban India and rural Nepal; data on request). Additional covariates in the models included measures of the characteristics of the pregnancy and delivery, maternal socio-demographic characteristics, and the location in which the delivery occurred. We created an indicator variable to identify women experiencing serious problems during pregnancy or delivery, with women reporting symptoms of pre-eclampsia (blurred vision or swelling of face and hands), symptoms of eclampsia (fits or seizures during pregnancy or delivery), hæmorrhage during delivery, or labour lasting more than 24 hours considered to have serious complications. Other characteristics of the pregnancy and delivery considered in the analysis included full utilisation of antenatal care, number of prior pregnancies, and multiple pregnancy. Full utilization of antenatal care was defined as four or more visits to antenatal care, with at least one visit to a skilled provider. Number of pregnancies was entered as an ordered categorical variable, with categories of one (first), two, three, or four or more pregnancies.

Maternal socio-demographic characteristics included in the models were: mother's age at delivery, her educational attainment, and household assets. Mother's age was entered into the model as a categorical measure in 10-year groups. Educational attainment was entered as a categorical variable using the following categories: no formal education, primary education, secondary education, or bachelor's degree or higher. To develop an asset index, we used polychoric factor analysis on data on common assets and amenities found in the mother's household, and grouped the resulting factor scores into quartiles.[33] Assets and amenities included electricity, radio or cassette player, electric fan (Bangladesh and India only), television, refrigerator (Bangladesh and India only), telephone (Bangladesh and Nepal only), generator (India only), and bicycle. All models were additionally adjusted for location (Bangladesh, India, Nepal), and year of interview in three-year groups. The data were collected in a stratified, cluster-sampled survey, and we accounted for survey design in the analysis using a fixed effect for stratum and a random effect for cluster.

Statistical analysis

We used frequencies to describe caesarean section rates by delivery location at each site. We used the Generalized Linear Latent And Mixed Models (GLLAMM) procedure in Stata 13.1, with adaptive

For peer review only - http://bmjopen.bmj.com/site/about/guidelines.xhtml

quadrature for binary outcomes, to estimate the crude association between type of delivery facility and caesarean section.[34 35] We identified other maternal, pregnancy, and delivery characteristics potentially associated with caesarean section using existing literature, especially studies resulting from the WHO multi-country surveys, and entered these in adjusted models to explore how they modified the association between type of delivery facility and caesarean section, and their individual association with caesarean section. Some South Asian and Latin American studies have detected a strong association between maternal education and caesarean delivery. [7 36 37] We fitted models including indicator variables for each group of mothers by education and type of delivery facility to explore differences in the strength of association between caesarean delivery and private facility by mother's education. To account for the sampling procedure used in rural Nepal, models were adjusted using pweights (probability of selection within cluster); these weights were rescaled to reflect the total number of institutional deliveries.

Ethical approval

The trials that provided data for this study received ethical approval from the following committees: the ethics committee of the Diabetic Association of Bangladesh (Perinatal Care Project, Bangladesh Diabetes Somity, BADAS); an independent ethics committee in Jamshedpur, Jharkhand, India; the Nepal Health Research Council; the Municipal Corporation of Greater Mumbai and the Independent Ethics Committee for Research on Human Subjects (Mumbai, India); the ethics committee of the Institute of Child Health, University College London.

RESULTS

Table 2 describes the characteristics of institutional births and caesarean deliveries by location. We analysed data for 45,327 births: 21,560 in rural Bangladesh, 8,541 in rural India, 10,236 in urban India, and 4,931 in rural Nepal. The proportion of women delivering in health facilities varied widely between locations, from 90% in urban India to 21% in rural India There were also large variations in the proportion of women delivering in private/charitable facilities rather than public facilities, with the highest (77%) in rural India and the lowest (10%) in rural Nepal. The proportion of women giving birth by caesarean section in private rather than public facilities also varied widely between settings. In Bangladesh, only 21% of women delivered in a health facility, around half of them in the private/charitable sector, but 73% of private facility births were by caesarean section. In rural Nepal, 30% of the 162 private facility births were by caesarean section. In informal settlements of Mumbai, 15% of public facility deliveries were caesareans, compared with 18% of private facility deliveries. In rural India, caesarean sections were more commonly performed in public health facilities than private or charitable facilities (15% vs. 5%). **Supplemental table 1** describes the characteristics of institutional deliveries (caesarean or non-caesarean) according to type of delivery facility (private or public), maternal

For peer review only - http://bmjopen.bmj.com/site/about/guidelines.xhtml

socio-economic and socio-demographic characteristics, pregnancy and delivery characteristics, and year of delivery.

Table 3 shows crude and adjusted measures of association between type of delivery facility (private or public) and caesarean section for each location. Delivering in a private health facility was associated with an increased odds of caesarean section in all but one location (rural India). The relative odds of a caesarean in a private facility were greatest in Bangladesh (OR: 6.82, 95% CI: 5.96, 7.81), followed by Nepal (OR: 2.42, 95% CI: 1.48, 3.94) and urban India (OR 1.36, 95% CI: 1.21, 1.52). These associations persisted and were only mildly attenuated when adjusted for maternal characteristics, pregnancy and delivery characteristics, and year of delivery (Bangladesh AOR: 5.91, 95% CI: 5.15, 6.78; Nepal AOR: 2.37, 95% CI: 1.62, 3.44; urban India AOR: 1.22, 95% CI: 1.09, 1.38).

Women who had four or more antenatal check-ups were significantly more likely to have a caesarean delivery in rural Bangladesh and Nepal, with positive but non-significant trends in all other locations. Parity was not associated with caesarean delivery in any location. Having a serious health complication during pregnancy and delivery was associated with caesarean delivery in all locations except rural Bangladesh, where we observed a negative association (AOR: 0.87, 95% CI: 0.76, 1.00). Multiple birth was associated with an increased odds of caesarean section only in urban India and in rural Nepal. Higher maternal age was only associated with an increased odds of caesarean section in urban India. Maternal education was only associated with an increased odds of caesarean delivery in rural Bangladesh and urban India, with mothers with secondary education or Bachelor's degrees having higher odds (AOR: 1.44, 95% CI: 1.13, 1.84 and AOR 2.44, 95% CI: 1.52, 3.92 for Bangladesh and AOR: 1.22, 95% CI: 1.04, 1.42 and 1.62, 95% CI: 1.30, 2.02 for urban India, respectively). There were significant positive associations only in rural Bangladesh, with small increases in odds of caesarean section for unit increase in wealth quartile (AOR comparing wealthiest to poorest group in Bangladesh: 1.36, 95% CI: 1.09,1.70), and in urban India, for the wealthiest quartile (AOR: 1.50, 95% CI: 1.27, 1.78).

We found interactive associations between maternal education and private facility delivery in two of four sites, with including an interaction improving model fit in two of the four analyses (p=0.021 in rural Bangladesh and p<0.001 in urban India; **table 4 and supplemental Table 2**). In Bangladesh, there was a pronounced positive educational gradient in caesarean delivery within those who delivered in private facilities, as well as a ~~clear~~ positive association between private facility delivery and caesarean delivery. Women with bachelor's degrees delivering in private facilities had 11 times greater odds of delivering by caesarean (AOR 11.09, 95% CI: 6.28, 19.57) than did women with no formal education delivering in public facilities. Women with no formal education delivering in private facilities had nearly seven times the odds of caesarean compared with the reference group (AOR 6.94, 95% CI: 4.64, 10.39). However, the

For peer review only - http://bmjopen.bmj.com/site/about/guidelines.xhtml

interaction between educational attainment was negative, suggesting that women holding bachelor's degrees are somewhat less likely than women with secondary degrees to deliver with caesarean (**supplemental table 2**). A ~~similar~~ positive gradient was seen among women delivering in public facilities, with women with bachelor's education having four times greater odds of caesarean delivery in public facilities than women with no formal education (AOR 4.55, 95% CI: 2.22, 9.33). There was also an educational gradient among women delivering in private facilities in urban India. Educated mothers delivering in private facilities were more likely to deliver by caesarean than were mothers in other groups (AOR 2.10; 95% CI: 1.61, 2.75). However, there was no apparent gradient by educational attainment among women delivering in public facilities.

Formatted: Font: Bold

DISCUSSION

Our analysis of data from over 45,000 births confirms the findings of other studies identifying differences in caesarean rates between public and private facilities, and suggests that, even in underserved areas in South Asia, caesareans without medical indication are of concern. In three of four locations, rates of caesarean section were higher in private/charitable facilities than in public facilities. The findings from Bangladesh are particularly noteworthy as they show much greater odds of caesarean section in private facilities, concurring with previous analyses.[37] This was also the only location where serious complications in pregnancy and delivery were not associated with caesarean delivery, suggesting that obstetric surgery was performed over and above clinical need.

Our findings confirm the results of earlier studies of the prevalence of caesarean delivery in South Asia, and indicate that high rates can be found in underserved rural areas. In India, a recent analysis of 2010-11 Annual Health Survey (AHS) data from 284 districts in nine States, including Jharkhand, found that the median caesarean section rate in the private sector was 28%, compared with 5% in the public sector.[38] There appear to be strong financial incentives for surgical procedures in the private sector.[39] In a rural, largely indigenous part of eastern India where more mothers delivered in private/charitable facilities than in public facilities, more caesarean sections were performed in the public sector. In this particular setting, our field experience suggests that only women with serious complications would go to a facility and have a caesarean section, especially as JSY was not yet available in the study areas at the time of data collection. Such women are likely to have experienced multiple referrals from either ill-equipped public or private facilities not wanting to take the risk of admission. It is however possible that preference for the private sector has changed in Jharkhand since the advent of JSY, and further disaggregated analyses of Annual Health Survey data would allow a more contemporary exploration of State-level variations in caesarean section rates between public and private sectors.

For peer review only - http://bmjopen.bmj.com/site/about/guidelines.xhtml

In Mumbai slums with a high uptake of institutional births and a 60/40 split in favour of the public sector for delivery care, we found no difference in caesarean section rates between the two sectors, which is somewhat surprising given the high rates of caesareans observed in the private sector in nine other states of India (excluding Maharashtra). At least two scenarios may be relevant in this setting. First, public sector hospitals are able to provide caesarean section, while smaller private maternity homes may not be able to. Second, the costs of caesarean section borne by private sector providers are higher in Mumbai, and families may be unwilling to bear these additional costs. This provides motivation for private providers not to provide caesareans, and makes it likely that women experiencing complications move into the public sector. The variety of facility options in an urban environment means that mothers are able to choose between different types of facilities, while in rural areas only one provider may be accessible.

Analyses using the most recent Bangladesh DHS found that, in 2011, three in five facility births were delivered by caesarean section. This reflects a historical trend: in 2001-2003, nearly half of deliveries in private facilities in Bangladesh were already by caesarean section.[18] Our data from rural, socio-economically disadvantaged communities in three districts of Bangladesh confirm these population-level findings, but also suggest that high caesarean section rates in private facilities are not merely an issue for wealthy urban mothers. Although some research from Bangladesh suggests that mothers may have a preference for caesarean delivery because of fear of labour pain or a desire to select an auspicious date for the birth, other studies also highlight women's fears of caesarean section and their distrust of health providers who recommend them, mainly because of the high costs associated with the procedure.[37 40 41] A qualitative study involving twenty women who had experienced obstetric complications in Matlab in 2008-9 found that most of the fourteen women who had undergone caesareans had spent over Tk 14,999 (US$ 217) on the procedure, which was approximately one-third of GDP per capita at the time.[41] The lack of association between caesareans and complications in pregnancy or delivery and multiple births, coupled with the high financial incentives given to providers for performing caesarean sections and the requirement for junior doctors to 'practise' their surgical skills, further suggest that obstetric surgery is being used over and above clinical need. A possible explanation for the significant interactive associations between maternal education and caesarean delivery in both public and private facilities in Bangladesh is that well-educated women may be delivering in more expensive or highly rated institutions, which may in turn be more likely to do caesarean sections for financial reasons and if they act as training centres for junior doctors.

In our sample of institutional deliveries from rural Nepal, 16% of facility births were by caesarean, which is higher than the national average: the 2011 Nepal DHS found an overall national caesarean section rate of 5%, with sections more commonly performed for births to highly educated mothers (13%) and

For peer review only

mothers in the highest wealth quintile (14%). [26] It is possible that women with complications are more likely to deliver in facilities, and also that, as in Bangladesh, private providers are motivated by financial incentives to conduct caesareans more frequently than strictly necessary. Women who can afford to seek care in private facilities may also be more willing or able to pay for caesarean sections, and providers may conduct more of them to increase their income.

A 2010 analysis of DHS data examining the role of the private sector in maternity care in 16 countries found evidence of a trend towards privatisation in delivery care between the 1990s and mid-2000s, but with strong differences between countries, which might reflect the heterogeneous nature of this sector both between and within countries. [42] This DHS study highlighted the need for more context-specific data on the nature of the private sector in low and middle-income countries, and its role in maternity care. Further research might focus on understanding the motivations and experiences of women undergoing caesarean sections in private facilities in South Asian settings, pathways for switching between public and private sectors in the event of obstetric complications, a more comprehensive tally of the financial incentives (official or non-official) that motivate private providers to carry out caesareans in each setting, and the consequences of increased caesarean sections in the private sector for maternal and neonatal health outcomes.

In our study, locations with higher prevalence of caesarean deliveries also showed a positive educational gradient even after adjusting for wealth measured by household assets. Moreover, there was an interactive association between education and type of facility, with highly educated women particularly likely to receive caesarean deliveries in private facilities in ~~Bangladesh and~~ urban India. Literature exploring the determinants of caesarean delivery emphasizes that multiple influences drive a woman's decision to deliver by caesarean. Profit considerations at the facility level may prompt some providers to urge women to receive unnecessary procedures, while women themselves may prefer caesareans for cultural reasons, fear of painful deliveries, or because they believe it to be safer. [36 43-45] Within the client-provider interaction, providers may be more likely to acquiesce to a request for caesarean from a highly educated woman; and such women may be more likely to accept advice from a provider. [19 46] Our findings lend support to the hypothesis that, while provider-level factors are probably partially responsible for the rapid increase in caesarean deliveries, it is also necessary to consider women's own preferences and decision-making processes and how they are shaped by social and cultural factors.

Strengths and limitations

The strengths of the study were its large sample size and focus on underserved communities, which are a priority for public health interventions in South Asia. It had five main limitations. It was not a nationally representative study, and districts or clusters were sampled purposively from previous cRCTs. This limits

For peer review only - http://bmjopen.bmj.com/site/about/guidelines.xhtml

the generalisability of our findings to geographical settings outside the study areas. The study was cross-sectional, and therefore only able to suggest associations rather than causal relationships. The data also did not include key predictors of caesarean section such as breech presentation, and whether the previous delivery was by caesarean, which limits the completeness of our analysis of determinants. In addition, levels of serious complications in pregnancy and delivery varied considerably between locations and were often higher than expected, suggesting potential over-reporting and limited reliability as an indicator of complications. Finally, some variables had small denominators, and others had high levels of missing data (for example, maternal age in rural India).

Conclusions

Our study found that delivering in a private health facility was associated with an increased odds of caesarean section in three of four South Asian locations, and that the associations persisted after adjustments for maternal, pregnancy and delivery characteristics, and year of delivery. We also found significant interactive associations between maternal education and caesarean delivery in Bangladesh (both private and public facilities) and urban India (private facilities only). These results lend support to the hypothesis that increased caesarean section rates in these three South Asian countries may in part be driven by the private sector, but also suggest that, in some settings such as Bangladesh and urban India, demand from more educated mothers may play a part. These findings call for greater, local understanding of the role of private providers in maternity care, together with careful examination of the consequences of increased caesarean sections in the private sector for maternal and neonatal health outcomes.

Competing interests

None to declare

Contributors

MN and AP had the original idea for the study. MN designed the data analysis strategy in discussion with AP. MN, GA, AS, CS and NS prepared the data. MN performed the analysis and prepared the tables. NN, PT, NSM, DO, KA, DM and AC are responsible for the trials. AP wrote the first draft of the paper and all authors read and commented on the draft. All authors read and approved the final manuscript.

For peer review only

Funding

MN and NS are funded through a Wellcome Trust Strategic Award (085417MA/Z/08/Z). In Bangladesh, the Perinatal Care Project trial (covering KA and AK) was registered with trial number ISRCTN54792066 and has received funding from the Big Lottery Fund and the Wellcome Trust. In Eastern India, the Ekjut trial (covering NN and PT) was registered with trial number ISRCTN21817853 and funded by the Health Foundation. The Dhanusha (Nepal) trial (covering DSM, NS, BPS, and CS) was registered with trial number ISRCTN87820538 and has received funding from: the UBS Optimus Foundation; USAID; DfID and the Wellcome Trust. The City Initiative for Newborn Health trial led by SNEHA (Mumbai, India) was registered with trial number ISRCTN96256793 and has received funding from the ICICI Foundation for Inclusive Growth and the Wellcome Trust. GA, NSM, and DO are currently funded by The Wellcome Trust (091561/Z/10/Z). The funders played no part in the development of the article or the decision to submit it for publication.

Provenance and peer review

Not commissioned; externally peer reviewed.

REFERENCES:

1. World Health Organisation and UNICEF. Countdown 2015: Building a future for women and Children, the 2012 Report. http://countdown2015mnch.org/documents/2012Report/2012-Complete.pdf [Accessed 9th November 2013]
2. Campbell OM, Graham WJ. Strategies for reducing maternal mortality: getting on with what works. *The Lancet* 2006; 368: 1284-1299.
3. Ronsmans C, De Brouwere V, Dubourg D, *et al*.. Measuring the need for life-saving obstetric surgery in developing countries. *BJOG* 2004; 111: 1027-30.
4. World Health Organization. Appropriate technology for birth. *The Lancet* 1985; 2: 436-7.
5. Betrán AP, Merialdi M, Lauer JA, *et al*. Rates of caesarean section: analysis of global, regional and national estimates. *Paediatric and Perinatal Epidemiology* 2007; 21: 98-113.
6. Cavallaro FL, Cresswell JA, França GVA, *et al*. Trends in Caesarean delivery by country and wealth quintile: cross-sectional surveys in southern Asia and sub-Saharan Africa. *WHO Bulletin* 2013; 91: 897-972.
7. Souza JP, Gülmezoglu A, Lumbiganon P, *et al*. WHO Global Survey on Maternal and Perinatal Health Research Group. Caesarean section without medical indications is associated with an increased risk of adverse short-term maternal outcomes: the 2004–2008 WHO Global Survey on Maternal and Perinatal Health. *BMC Medicine* 2010; 8:71.
8. Kyu HH, Shannon HS, Georgiades K, Boyle MH. Caesarian delivery and neonatal mortality rates in

For peer review only - http://bmjopen.bmj.com/site/about/guidelines.xhtml

46 low- and middle-income countries: a propensity score matching and meta-analysis of demographic health survey data. *International Journal of Epidemiology* 2013; 1-11.

9. Gibbons L, Belizán JM, Lauer JA, *et al*. The global numbers and costs of additionally needed and unnecessary caesarean sections performed per year: overuse as a barrier to universal coverage. World Health Report (2010), Background paper. Geneva: World Health Organization.
10. Mitra SN, Nawab Ali M, Islam S, *et al*. Bangladesh Demographic and Health Survey, 1993-1994. Calverton, Maryland: National Institute of Population Research and Training (NIPORT), Mitra and Associates, and Macro International Inc, 1994.
11. National Institute of Population Research and Training (NIPORT), Mitra and Associates, and ICF International. Bangladesh Demographic and Health Survey 2011. Dhaka, Bangladesh and Calverton, Maryland, USA: NIPORT, Mitra and Associates, and ICF International, 2013.
12. International Institute for Population Sciences (IIPS) and Macro International. National Family Health Survey (NFHS 1), 1992–93. India, Mumbai: IIPS, 1995.
13. International Institute for Population Sciences (IIPS), Macro International. National Family Health Survey (NFHS 3), 2005–06; India: Volume I. Mumbai: IIPS, 2007.
14. Pradhan A, Aryal RH, Regmi G, *et al*. Nepal Family Health Survey 1996. Kathmandu, Nepal and Calverton, Maryland: Ministry of Health [Nepal], New ERA, and Macro International Inc, 1997.
15. Ministry of Health and Population (MOHP) [Nepal], New ERA, and ICF International Inc. Nepal Demographic and Health Survey 2011. Kathmandu, Nepal: Ministry of Health and Population, New ERA, and ICF International, Calverton, Maryland, 2012.
16. Ajeet S, Nandkishore K. The boom in unnecessary caesarean surgeries is jeopardizing women's health. *Health Care Women International* 2013; 34: 513-521.
17. Sreevidya S, Sathiyasekaran BW. High caesarean rates in Madras (India): a population-based cross-sectional study. *BJOG* 2003; 110: 106-111.
18. Koblinksi M, Anwar I, Mridha MK, *et al*. Reducing maternal mortality and improving maternal health: Bangladesh and MDG 5. *J Health Popul Nutr* 2008; 26: 280-294.
19. Leone T, Padmadas SS, Matthews Z. Community factors influencing rising caesarean section rates in developing countries: an analysis of six countries. *Soc Sci Med* 2008; 67: 1236-46.
20. Mazzoni A, Althabe A, Liu NH, *et al*. Women's preference for caesarean section: a systematic review and meta-analysis of observational studies. *BJOG* 2011; 118: 391-399.
21. Tripathy P, Nair N, Barnett S, et al. Effect of a participatory intervention with women's groups on birth outcomes and maternal depression in Jharkhand and Orissa, India: A cluster-randomised controlled trial. *Lancet* 2010; 375: 1182–1192.
22. Azad K, Barnett S, Banerjee B, *et al*. Effect of scaling up women's groups on birth outcomes in three rural districts in Bangladesh: a cluster-randomised controlled trial. *Lancet* 2010; 375: 1193–1202.

For peer review only

For peer review only - http://bmjopen.bmj.com/site/about/guidelines.xhtml

23. Fottrell E, Azad K, Kuddus A, *et al*. The effect of increased coverage of participatory women's groups on neonatal mortality in Bangladesh: a cluster-randomised trial. *JAMA Pediatrics* 2013; 167: 816-825.

24. Shah More N, Bapat U, Das S, *et al*. Cluster-randomised controlled trial of community mobilisation in Mumbai slums to improve care during pregnancy, delivery, postpartum and for the newborn. *Trials* 2008; 9:7.

25. Shrestha BP, Bhandari B, Manandhar DS, *et al*. Community interventions to reduce child mortality in Dhanusha, Nepal: study protocol for a cluster randomized controlled trial. *Trials* 2011; 12: 136.

26. Prost A, Colbourn T, Seward N, *et al*. Women's groups practising participatory learning and action to improve maternal and newborn health in low-resource settings: a systematic review and meta-analysis. *Lancet* 2013; 381: 1736–46.

27. Schmidt J-0, Ensor T, Hossain A, Khan S. Vouchers as demand side financing instruments for health care: A review of the Bangladesh maternal voucher scheme. *Health Policy* 2010; 96: 98–107.

28. Ahmed S, Khan MM. A maternal health voucher scheme: what have we learned from the demand-side financing scheme in Bangladesh? *Health Policy & Planning* 2011; 6:25–32.

29. Lim SS, Dandona L, Hoisington JA, *et al*. India's Janani Suraksha Yojana, a conditional cash transfer programme to increase births in health facilities: an impact evaluation**.** *Lancet* 2010; 375: 209-23.

30. Shah More N, Alcock G, Bapat U, *et al*. Tracing pathways from antenatal to delivery care for women in Mumbai, India: cross-sectional study of maternity in low-income areas. International Health 2009; 1: 71–77.

31. Government of India, Ministry of Health & Family Welfare. Janani Suraksha Yojana: Guidelines for Implementation, 2006. http://www.mohfw.nic.in/NRHM/./guidelines/JSY_guidelines_09_06.pdf

32. Witter S, Khadka S, Nath H, Tiwari S. The national free delivery policy in Nepal: early evidence of its effects on health facilities. *Health Policy and Planning* 2011; 26: ii84–ii91.

33. Holgado-Tello FP,Chacón–Moscoso S, Barbero-Garcia I, Vila-Abad E. Polychoric versus Pearson correlations in exploratory and confirmatory factor analysis of ordinal variables. *Quality & Quantity* 2010; 44 (1): 153-166.

34. Rabe-Hesketh S, Skrondal A, Pickles A. Maximum likelihood estimation of limited and discrete dependent variable models with nested random effects. *Journal of Econometrics* 2005; 128: 301-323.

35. StataCorp. 2013. *Stata Statistical Software: Release 13*. College Station, TX: StataCorp LP.

36. Potter J, Berquó E, Perpétuo I, *et al*. Unwanted Caesarean Sections among Public and Private

For peer review only - http://bmjopen.bmj.com/site/about/guidelines.xhtml

Patients in Brazil: Prospective Study. *BMJ* 2001; 323: 1155-8.

37. Kamal SMM. Preference for institutional delivery and caesarean sections in Bangladesh. *J Health Popul Nutr* 2013; 31: 96-109.
38. Randive B, Diwan V, De Costa A. India's Conditional Cash Transfer Programme (the JSY) to Promote Institutional Birth: Is There an Association between Institutional Birth Proportion and Maternal Mortality? *PLoS ONE* 2013; 8(6): e67452. doi:10.1371/journal.pone.0067452
39. Chatterjee S, Laxminarayan R. Costs of surgical procedures in Indian hospitals. *BMJ Open* 2013; 3: e002844.
40. Parkhurst JO, Rahman SA. Life saving or money wasting? Perceptions of caesarean sections among users of services in Rural Bangladesh. *Health Policy* 2007; 80: 392-401.
41. Khan R, Blum LS, Sultana M, et al. An Examination of Women Experiencing Obstetric Complications Requiring Emergency Care: Perceptions and Sociocultural Consequences of Caesarean Sections in Bangladesh. *J Health Popul Nutr 2012*; 30: 159-171.
42. Pomeroy A, Koblinksy M, Alva S. Private Delivery Care in Developing Countries: Trends and Determinants. DHS Working document 2010; No.76. http://dhsprogram.com/pubs/pdf/WP76/WP76.pdf [Accessed 3rd March 2014].
43. Sufang G, Padmadas SS, Fengmin Z et al. Delivery settings and caesarean section rates in China. *Bull WHO* 2007; 85: 733-820.
44. Lo JC. Patients' attitudes vs. physicians' determination: implications for cesarean sections. *Soc Sci Med* 2003; 57: 91-6.
45. McCourt C, Weaver J, Statham H, *et al.* Elective cesarean section and decision making: a critical review of the literature. *Birth* 2007; 34: 65-79.
46. Ghetti C, Chan BKS, Guise J-M. Physicians' responses to patient-requested caesarean delivery. *Birth* 2004; 31: 280-4.

For peer review only - http://bmjopen.bmj.com/site/about/guidelines.xhtml

Table 1: Characteristics of studies and populations

Study (Country)	Bangladesh (rural)	India (rural)	Nepal (rural)	India (urban)
**Location**	Three districts: Bogra, Maulvibazaar and Faridpur	Three districts of Jharkhand and Odisha: Keonjhar, West Singhbhum and Saraikela	Dhanusha district (Terai)	Mumbai slums
**Period**	2005 – 2011	2005-2008	2008-2011	2006-2009
**Estimated population**	532,900	114,000	240,000	283,000
**Cluster characteristics**	Villages making up a union	8-10 villages with residents classified as Scheduled Tribe or Other Backward Class	Village Development Committee	Slum areas in six municipal wards of Mumbai
**Method of cluster identification**	Purposive sampling of three districts and clusters within districts	Purposive sampling of three districts and clusters within districts	Random sampling of 60 clusters from a list of 79 suitable clusters in one district	92 clusters in six municipal wards identified using municipal documents, surveys, discussions with key informants, and site visits. Random selection of 48 clusters for randomized allocation.
**Clusters, n**	9	18	30	24
**Cluster and individual follow-up**	All clusters followed up<br>Interviews completed after 82% of identified births in control areas in Phase 1, and 99% of births in Phase 2	All clusters followed up<br>Interviews completed after 98% of identified births	All clusters followed up	All clusters followed up<br>Interviews completed after 83% of identified births
**Maternal mortality ratio**	254.3	668.1	Unknown	206.2
**Health facilities available in control areas**	Public facilities: District Hospitals; Maternal and Child Welfare Centres; Upazilla Health Complexes. Private facilities: small-to-medium-size clinics; BRAC (NGO) facilities where deliveries do not take place; larger private hospitals with and without Comprehensive emergency obstetric care (CEmOC) facilities.	Public facilities: District Hospitals; Primary Health Centres (PHCs) in which deliveries can notionally take place but that are not usually equipped for CEmOC; Community Health Centres (CHCs) acting as referral centres for PHCs, covering a population of around 80,000 with EmOC facilities; district hospitals. Private and charitable facilities: medium-sized missionary hospitals with EmOC facilities.	Public facilities: three Primary Health Care Centres, three Health Posts and 24 Sub-Health Posts, none of which are equipped for CEmOC. These health facilities refer to the public Zonal Tertiary Hospital and various private providers in the district headquarters and nearby medical college, which have facilities for caesarean sections.	Public facilities: Municipal tertiary hospitals, general hospitals, and maternity homes. Private facilities: specialty hospitals, general hospitals, and maternity homes.

For peer review only

For peer review only - http://bmjopen.bmj.com/site/about/guidelines.xhtml

Table 2: Characteristics of institutional births and caesarean deliveries by location

	Births	Institutional births						Caesarean section births			
		All		Public facility		Private/charitable facility		Public facility		Private/charitable facility	
	(n)	(n)	(% all births)	(n)	(% institutional births)	(n)	(% institutional births)	(n)	(% deliveries in public facilities)	(n)	(% deliveries in private facilities)
**Bangladesh (rural)**	21,560	4,592	21%	2,053	45%	2,539	55%	589	29%	1,852	73%
**India (rural)**	8,541	1,816	21%	421	23%	1,395	77%	64	15%	66	5%
**India (urban)**	10,236	9,259	90%	5,489	59%	3,770	41%	811	15%	697	18%
**Nepal (rural)**	4,931	1,586	32%	1,424	90%	162	10%	201	14%	48	30%

* Nepal numbers weighted using women's probability of selection within each cluster, scaled to total number of institutional births. (Unweighted Nepal totals: 4,990 births and 1,586 institutional births)

For peer review only

Table 3: Mutually adjusted associations of type of facility of delivery and other selected determinants with caesarean section delivery, by location

	Bangladesh (rural) ORs (95% CIs)		India (rural) ORs (95% CIs)		India (urban) ORs (95% CIs)		Nepal (rural)* ORs (95% CIs)	
	Crude	Adjusted	Crude	Adjusted	Crude	Adjusted	Crude	Adjusted
**Health facility characteristics**								
Public health facility (ref)								
Private health facility	6.82 (5.96,7.81)	5.91 (5.15,6.78)	0.39 (0.25,0.61)	0.46 (0.29,0.73)	1.36 (1.21,1.52)	1.22 (1.09,1.38)	2.42 (1.48,3.94)	2.37 (1.62,3.44)
**Pregnancy and delivery characteristics**								
4+ antenatal care visits (fewer or none = ref)**		1.46 (1.26,1.69)		1.49 (0.96,2.32)		1.06 (0.93,1.21)		1.92 (1.43,2.58)
Birth order								
1 (ref)								
2		0.95 (0.80,1.13)		1.25 (0.80,1.95)		0.65 (0.56,0.75)		1.41 (1.02,1.94)
3		1.19 (0.94,1.52)		0.36 (0.15,0.87)		0.60 (0.50,0.71)		0.58 (0.37,0.92)
4+		1.17 (0.90,1.53)		0.36 (0.15,0.85)		0.39 (0.32,0.48)		1.00 (0.54,1.84)
Serious complications in pregnancy/delivery*** (no complications=ref)		0.87 (0.76,1.00)		1.77 (1.17,2.67)		1.71 (1.39,2.11)		4.87 (2.51,9.47)
Multiple birth (ref=single)		0.93 (0.65,1.32)		1.57 (0.52,4.75)		3.01 (2.14,4.23)		3.42 (1.77,6.61)
**Maternal characteristics**								
Age (years)								
15-24 (ref)								
25-34		1.12 (0.94,1.34)		1.81 (1.14,2.86)		1.45 (1.27,1.66)		1.52 (0.63,1.94)
35+		1.10 (0.77,1.58)		2.11 (0.63,7.07)		1.79 (1.27,2.53)		1.55 (0.55,1.47)

For peer review only - http://bmjopen.bmj.com/site/about/guidelines.xhtml

	Bangladesh (rural) ORs (95% CIs)		India (rural) ORs (95% CIs)		India (urban) ORs (95% CIs)		Nepal (rural)* ORs (95% CIs)	
	Crude	Adjusted	Crude	Adjusted	Crude	Adjusted	Crude	Adjusted
Maternal education****								
No formal education (ref)								
Primary education		1.07 (0.83,1.38)		0.98 (0.39,2.47)		1.13 (0.85,1.49)		0.90 (0.59,1.39)
Secondary education		1.44 (1.13,1.84)		1.11 (0.68,1.80)		1.22 (1.04,1.42)		1.37 (0.97,1.94)
Bachelor’s degree or higher		2.44 (1.52,3.92)		1.20 (0.43,3.31)		1.62 (1.30,2.02)		- -
Household wealth quintile								
1st (poorest, ref)								
2nd		1.31 (1.06,1.63)		1.61 (0.69,3.75)		1.14 (0.77,1.68)		1.11 (0.63,1.94)
3rd		1.41 (1.10,1.82)		1.36 (0.58,3.21)		1.13 (0.95,1.33)		0.90 (0.55,1.47)
4th (wealthiest)		1.36 (1.09,1.70)		2.16 (0.87,5.33)		1.50 (1.27,1.78)		0.99 (0.52,1.90)
Year of delivery								
2004-2006 (ref: BD, IN)								-
2007-2009 (ref: NP rural)		0.90 (0.74,1.10)		0.90 (0.61,1.33)		1.14 (1.09,1.38)		- -
2010-2012		1.15 (0.94,1.41)		- -		- -		1.29 (0.91,1.82)
N	4,592	4,592	1,816	1,816	9,259	9,259	1,586	1,586

* Nepal numbers weighted using women's probability of selection within each cluster, scaled to total number of institutional births.

** At least one visit with skilled provider

***Includes: symptoms of eclampsia (fits, seizures, convulsions, or unconsciousness during pregnancy or delivery); reduced or no fetal movement; labour lasting more than 24 hours.

****For the Nepal data, two respondents with bachelor's degrees (2 respondent total, 1 delivering in institution) were combined with respondents with secondary education.

All analyses additionally adjusted for survey design using fixed effect of stratum and random effect of cluster.

For peer review only

Table 4: Interactive associations between type of delivery facility, maternal education, and caesarean delivery, by location

	Bangladesh (rural)		India (rural)		India (urban)		Nepal (rural)*	
	(n)	AOR, 95% CI	(n)	AOR, 95% CI	(n)	AOR, 95% CI	(unweighted n)	AOR, 95% CI
Public facility - no maternal education (ref)	202	-	109	-	1,396	-	822	-
Public facility - primary education	448	1.07 (0.76,1.50)	17	0.92 (0.18,4.77)	357	0.88 (0.61,1.27)	196	1.04 (0.65,1.65)
Public facility - secondary education	202	1.63 (1.18,2.24)	604	1.09 (0.52,2.26)	920	1.1 (0.91,1.33)	413	1.45 (1.04,2.02)
Public facility - bachelor's degree	448	4.58 (2.24,9.38)	100	1.55 (0.41,5.89)	180	1.06 (0.76,1.47)	-	- -
Private facility - no education	332	7.24 (4.82,10.87)	109	0.46 (0.21,1.01)	1396	0.91 (0.70,1.19)	100	2.83 (1.73,4.61)
Private facility - primary education	578	7.58 (5.36,10.71)	17	0.47 (0.14,1.58)	357	1.52 (0.99,2.33)	16	0.86 (0.14,5.21)
Private facility - secondary education	1,102	9.03 (6.57,12.41)	274	0.52 (0.24,1.12)	3,382	1.3 (1.06,1.60)	39	3.01 (1.71,5.30)
Private facility - bachelor's degree	41	11.24 (6.37,19.85)	21	0.4 (0.08,2.00)	354	2.1 (1.61,2.75)	-	- -
N	3,353		1,251		8,342		1586	
p-value**	0.021		0.792		0.001		0.394	

All results adjusted for number of antenatal care visits, parity, medical indication for caesarean, multiple birth, maternal age (10-yr group), household assets, stratum, and cluster (random effect).
* Nepal numbers weighted using women's probability of selection within each cluster, scaled to total number of institutional births.

** p-value from -2 log-likelihood test comparing nested models with and without interaction terms

For peer review only - http://bmjopen.bmj.com/site/about/guidelines.xhtml

"Prevalence and determinants of caesarean section in private and public health facilities in underserved South Asian communities: cross-sectional analysis of data from Bangladesh, India, and Nepal"

Supplemental tables

Supplemental Table 1: Characteristics of institutional deliveries by characteristics of the mother, the delivery facility, and location

For peer review only

	Bangladesh (rural)				India (rural)				India (urban)				Nepal (rural)			
	Not caesarean	Caesarean	Total		Not caesarean	Caesarean	Total		Not caesarean	Caesarean	Total		Not caesarean	Caesarean	Total	
	(%)	(%)	(n)	(%)	(%)	(%)	(n)	(%)	(%)	(%)	(n)	(%)	(%)	(%)	(n)	(%)
**Health facility characteristics**																
Public health facility	68.1	24.1	2053	44.7	21.2	49.2	421	23.2	60.4	53.8	5489	59.3	91.5	80.7	1424	89.8
Private/charitable health facility	31.9	75.9	2539	55.3	78.8	50.8	1395	76.9	39.6	46.2	3770	40.7	8.5	19.3	162	10.2
**Pregnancy and delivery characteristics**																
4+ antenatal care visits*	25.2	38.8	1491	67.5	47.1	56.2	867	52.3	73.4	74.2	6810	26.4	41.3	53.6	686	43.2
Birth order																
1	47.1	47.4	2170	47.3	51.6	55.4	942	51.9	29.7	41.6	2932	31.7	50.1	46.7	786	49.6
2	25.9	26.1	1194	26	23	32.3	430	23.7	27.9	26.1	2554	27.6	22.5	27.1	368	23.2
3	12.4	13.8	603	13.1	11.7	5.4	204	11.2	19.4	17.6	1769	19.1	14.9	10.3	225	14.2
4+	14.6	12.7	625	13.6	13.7	6.9	240	13.2	23	14.7	2004	21.6	12.5	15.9	207	13
Serious complications in pregnancy/delivery**	53.4	43.5	2212	48.2	29.2	44.6	550	30.3	6.1	9.2	608	6.6	21.2	38.5	379	23.9
Multiple birth	3.6	3.9	173	3.8	2.1	3.1	40	2.2	1.4	3.6	163	1.8	2.2	7.8	48	3.1
**Maternal socio-demographic characteristics**																
Maternal age (years)																
15-24	59.8	55.8	2650	57.7	63.6	60	1150	63.3	53.1	51.5	4894	52.9	71.6	66.3	1123	70.8
25-34	34.4	39.5	1705	37.1	32.7	36.9	600	33	43.6	45.3	4060	43.8	26.4	31	431	27.2
35+	5.8	4.6	237	5.2	3.7	3.1	66	3.6	3.3	3.2	305	3.3	1.9	2.7	33	2.1
Maternal education																
No formal education	15.2	8.3	531	11.6	40.1	28.5	713	39.3	26.3	18.5	2316	25	58.4	57.2	924	58.2
Primary education	27.3	17.9	1025	22.3	6.6	4.6	117	6.4	6	4.9	537	5.8	13.6	10.8	209	13.2
Secondary education	55.7	68.6	2872	62.5	50.2	62.3	927	51	59.6	61.9	5550	59.9	28	32	453	28.6
Bachelor's degree or higher***	1.7	5.2	164	3.6	3.1	4.6	59	3.2	8.2	14.7	856	9.2	-	-	-	-

Household wealth quintile																
1st (poorest)	23	10.4	749	16.3	12.6	5.4	220	12.1	24.1	17.2	2125	23	14.2	13.4	223	14.1
2nd	32.4	31.5	1465	31.9	30	31.5	547	30.1	3	2.3	268	2.9	22	22.4	350	22.1
3rd	14	15.2	673	14.7	39.9	32.3	715	39.4	35.9	31.3	3254	35.1	33.9	30.5	529	33.4
4th (wealthiest)	30.5	42.9	1705	37.1	17.4	30.8	334	18.4	37	49.3	3612	39	29.9	33.6	483	30.5
**Year of delivery**																
2004-2006	19.2	15.2	782	17	36.7	40.8	672	37	46.4	42.2	4237	45.8	-	-	-	-
2007-2009	44.7	38.7	1907	41.5	63.3	59.2	1144	63	53.6	57.8	5022	54.2	45.1	45.7	717	45.2
2010-2012	36.1	46.1	1903	41.4	-	-	-	-	-	-	-	-	54.9	54.3	869	54.8
Total (n)	2151	2441	4592	100	1686	130	4592	100	7751	1508	9259	100	734	135	869	100

* Nepal numbers weighted using women's probability of selection within each cluster, scaled to total number of institutional births.

** At least one visit with skilled provider

***Includes: symptoms of pre-eclampsia (swollen face or limbs or blurred vision [not asked in Nepal]), eclampsia (fits, seizures, convulsions, or unconsciousness during pregnancy or delivery); haemorrhaging during delivery, labour lasting more than 24 hours.

****For the Nepal data, secondary and bachelor's (2 respondent total, 1 delivering in institution) were combined.

For peer review only

Supplemental Table 2: Interactive associations between caesarean delivery and mother's educational attainment and place of delivery by location

	**Bangladesh (rural)** OR, 95% CI	**India (rural)** OR, 95% CI	**India (urban)** OR, 95% CI	**Nepal (rural)** OR, 95% CI
Main associations				
Private delivery facility (public = ref)	7.24	0.47	0.91	2.83
	(4.82,10.87)	(0.21,1.02)	(0.70,1.19)	(1.43,2.56)
Mother's educational attainment (no education = ref)				
Primary education	1.07	0.91	0.88	1.04
	(0.76,1.50)	(0.18,4.75)	(0.61,1.27)	(0.65,1.65)
Secondary education	1.63	1.09	1.1	1.45
	(1.18,2.24)	(0.52,2.27)	(0.91,1.33)	(1.04,2.02)
Bachelor's or higher	4.59	1.55	1.06	-
	(2.24,9.40)	(0.41,5.88)	(0.76,1.47)	-
Interactive associations				
Primary education * private delivery	0.98	1.11	1.9	0.29
	(0.60,1.61)	(0.15,8.12)	(1.07,3.37)	(0.04,1.92)
Secondary education * private delivery	0.77	1.03	1.31	0.74
	(0.49,1.19)	(0.42,2.51)	(0.97,1.77)	(0.31,1.73)
Bachelor's or higher * private delivery	0.34	0.55	2.18	-
	(0.14,0.83)	(0.08,3.93)	(1.42,3.35)	-
N	4590	1812	9259	1586

For peer review only

STROBE 2007 (v4) checklist of items to be included in reports of observational studies in epidemiology*
Checklist for cohort, case-control, and cross-sectional studies (combined)

Section/Topic	Item #	Recommendation	Reported on page #
**Title and abstract**	1	(*a*) Indicate the study's design with a commonly used term in the title or the abstract	1
		(*b*) Provide in the abstract an informative and balanced summary of what was done and what was found	2
**Introduction**			
Background/rationale	2	Explain the scientific background and rationale for the investigation being reported	4
Objectives	3	State specific objectives, including any pre-specified hypotheses	4
**Methods**			
Study design	4	Present key elements of study design early in the paper	1, 5-6
Setting	5	Describe the setting, locations, and relevant dates, including periods of recruitment, exposure, follow-up, and data collection	5 and Table 1
Participants	6	(*a*) *Cohort study*—Give the eligibility criteria, and the sources and methods of selection of participants. Describe methods of follow-up<br>*Case-control study*—Give the eligibility criteria, and the sources and methods of case ascertainment and control selection. Give the rationale for the choice of cases and controls<br>*Cross-sectional study*—Give the eligibility criteria, and the sources and methods of selection of participants	6
		(*b*) *Cohort study*—For matched studies, give matching criteria and number of exposed and unexposed<br>*Case-control study*—For matched studies, give matching criteria and the number of controls per case	
Variables	7	Clearly define all outcomes, exposures, predictors, potential confounders, and effect modifiers. Give diagnostic criteria, if applicable	7
Data sources/ measurement	8*	For each variable of interest, give sources of data and details of methods of assessment (measurement). Describe comparability of assessment methods if there is more than one group	7
Bias	9	Describe any efforts to address potential sources of bias	7-8
Study size	10	Explain how the study size was arrived at	6
Quantitative variables	11	Explain how quantitative variables were handled in the analyses. If applicable, describe which groupings were chosen and why	7
Statistical methods	12	(*a*) Describe all statistical methods, including those used to control for confounding	7-8
		(*b*) Describe any methods used to examine subgroups and interactions	Table 4
		(*c*) Explain how missing data were addressed	7
		(*d*) *Cohort study*—If applicable, explain how loss to follow-up was addressed<br>*Case-control study*—If applicable, explain how matching of cases and controls was addressed	

For peer review only

		Cross-sectional study—If applicable, describe analytical methods taking account of sampling strategy	
		(*e*) Describe any sensitivity analyses	N/A
**Results**			
Participants	13*	(a) Report numbers of individuals at each stage of study—eg numbers potentially eligible, examined for eligibility, confirmed eligible, included in the study, completing follow-up, and analysed	7, 8
		(b) Give reasons for non-participation at each stage	7, 8
		(c) Consider use of a flow diagram	Not used
Descriptive data	14*	(a) Give characteristics of study participants (eg demographic, clinical, social) and information on exposures and potential confounders	Supplemental Table 1
		(b) Indicate number of participants with missing data for each variable of interest	
		(c) *Cohort study*—Summarise follow-up time (eg, average and total amount)	
Outcome data	15*	*Cohort study*—Report numbers of outcome events or summary measures over time	
		Case-control study—Report numbers in each exposure category, or summary measures of exposure	
		Cross-sectional study—Report numbers of outcome events or summary measures	Table 3
Main results	16	(*a*) Give unadjusted estimates and, if applicable, confounder-adjusted estimates and their precision (eg, 95% confidence interval). Make clear which confounders were adjusted for and why they were included	Table 3
		(*b*) Report category boundaries when continuous variables were categorized	
		(*c*) If relevant, consider translating estimates of relative risk into absolute risk for a meaningful time period	
Other analyses	17	Report other analyses done—eg analyses of subgroups and interactions, and sensitivity analyses	Table 4
**Discussion**			
Key results	18	Summarise key results with reference to study objectives	10
Limitations	19	Discuss limitations of the study, taking into account sources of potential bias or imprecision. Discuss both direction and magnitude of any potential bias	12
Interpretation	20	Give a cautious overall interpretation of results considering objectives, limitations, multiplicity of analyses, results from similar studies, and other relevant evidence	10-1
Generalisability	21	Discuss the generalisability (external validity) of the study results	12
**Other information**			
Funding	22	Give the source of funding and the role of the funders for the present study and, if applicable, for the original study on which the present article is based	13

*Give information separately for cases and controls in case-control studies and, if applicable, for exposed and unexposed groups in cohort and cross-sectional studies.

Note: An Explanation and Elaboration article discusses each checklist item and gives methodological background and published examples of transparent reporting. The STROBE checklist is best used in conjunction with this article (freely available on the Web sites of PLoS Medicine at http://www.plosmedicine.org/, Annals of Internal Medicine at http://www.annals.org/, and Epidemiology at http://www.epidem.com/). Information on the STROBE Initiative is available at www.strobe-statement.org.

For peer review only
